# MdDSK2a‐Like‐MdMTA Module Functions in Apple Cold Response via Regulating ROS Detoxification and Cell Wall Deposition

**DOI:** 10.1002/advs.202504405

**Published:** 2025-04-25

**Authors:** Nan Hou, Jieqiang He, Chana Bao, Fang Zhi, Xiaoxia Shen, Yu Liu, Chaoshuo Li, Tianle Fan, Xinyue Yang, Baohua Chu, Gege Qin, Zeyuan Liu, Chuang Mei, Bin Tan, Jiancan Feng, Fengwang Ma, Mickael Malnoy, Xuewei Li, Qingmei Guan

**Affiliations:** ^1^ State Key Laboratory of Crop Stress Biology for Arid Areas/Shaanxi Key Laboratory of Apple College of Horticulture Northwest A&F University Yangling Shaanxi 712100 China; ^2^ College of Horticulture Henan Agricultural University 63 Nongye Road Zhengzhou 450002 China; ^3^ The State Key Laboratory of Genetic Improvement and Germplasm Innovation of Crop Resistance in Arid Desert Regions (Preparation) Key Laboratory of Genome Research and Genetic Improvement of Xinjiang Characteristic Fruits and Vegetables Institute of Horticulture Crops Xinjiang Academy of Agricultural Sciences Urumqi 830091 China; ^4^ Research and Innovation Centre Fondazione Edmund Mach Via E. Mach San Michele all'Adige 38010 Italy

**Keywords:** autophagy, cold stress, m^6^A, post‐translation, proteasome

## Abstract

N^6^‐methyladenosine (m^6^A) is the most abundant internal RNA modification in eukaryotic cells. Although the importance of its roles in mRNA metabolism, plant development, and stress responses has been well documented, regulation of its machinery is largely unknown in plants. Here, it is reported that MdMTA positively regulates cold tolerance. Combining MeRIP‐seq and RNA‐seq, it is found that MdMTA regulates the m^6^A and expression levels of cold‐responsive genes under cold stress, including those involved in reactive oxygen species (ROS) detoxification and cell wall deposition. Further analysis reveals that MdMTA promotes ROS scavenging and the deposition of cellulose and hemicellulose by regulating the mRNA stability of the relevant genes under cold conditions. MdDSK2a‐like, a ubiquitin receptor protein, mediates MdMTA degradation by the 26S ubiquitin‐dependent proteasome and autophagy pathways. MdDSK2a‐like negatively regulates cold tolerance by reducing the m^6^A levels of MdMTA target genes. Consistently, MdDSK2a‐like inhibits ROS scavenging and the deposition of cellulose and hemicellulose under cold conditions. Genetic dissection shows that MdDSK2a‐like acts upstream of MdMTA in cold response. The results not only reveal the degradation of MdMTA, but also illustrate the molecular mechanism of the MdDSK2a‐like‐MdMTA module in m^6^A modification and cold response.

## Graphical abstract

Under cold stress, MdDSK2a‐like is degraded via the 26S proteasome pathway, thereby alleviating the degradation of MdMTA induced by the 26S proteasome and autophagy pathways mediated by MdDSK2a‐like. The accumulated MdMTA increases the m^6^A levels of cold‐responsive genes, thereby contributing to increased ROS detoxification, deposition of cell wall, as well as apple cold tolerance.

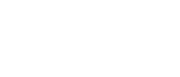

## 1 Introduction

As an environmental factor that has a significant impact on plant growth, productivity, and survival, cold stress limits the geographical distribution of various plants.^[^ ^1^ ^]^ Cold stress includes chilling stress (0–15 °C) that inhibits plant development and freezing stress (<0 °C) that damages the cell membrane.^[^ ^2^ ^]^ Before the onset of winter, plants have the ability to increase their cold tolerance after exposure to a period of low nonfreezing temperatures, a process known as cold acclimation (CA).^[^ ^3^ ^]^ Cold stress induces ROS accumulation, including hydrogen peroxide (H_2_O_2_), hydroxyl radical, and superoxide anion (O_2_^−^), therefore causes secondary stress called oxidative stress.^[^ ^4^ ^]^ To overcome the adverse effects of cold stress, plants have evolved diverse enzymatic and nonenzymatic mechanisms to eliminate ROS. Enzymatic antioxidants include peroxidase (POD), catalase (CAT), superoxide dismutase (SOD), and ascorbate peroxidase (APX),^[^ ^5^ ^]^ while nonenzymatic antioxidants include ascorbate, glutathione, carotenoids, tocopherols, and flavonoids.^[^ ^6^ ^]^ Plant cell wall is the site where ice crystals form during freezing process, and it can prevent damage to the plasma membrane.^[^ ^7^ ^]^ Plant cell wall is composed of pectin, cellulose, and hemicellulose,^[^ ^8^ ^]^ which are often deposited under cold stress and play a prominent role in abiotic stress tolerance.^[^ ^9^, ^10^ ^]^ In addition, plants have evolved complex molecular mechanisms to respond to cold stress, which include transcriptional regulations, post‐transcriptional modifications, and post‐translational modifications.^[^ ^11^, ^12^ ^]^ Apple is one of the most important perennial fruit crops and is vulnerable to freezing temperatures, especially during the spring period of floral development before bloom.^[^ ^13^ ^]^ Compared to traditional breeding, molecular breeding has been proved more feasible because of its shorter breeding period and higher efficiency.^[^ ^14^ ^]^ Therefore, understanding the mechanism of apple freezing tolerance is of importance for molecular breeding.

m^6^A, one of the most abundant post‐transcriptional modifications in eukaryotes, is a dynamically reversible process, which is installed, recognized, and removed by methyltransferases (writers), reader proteins (readers), and demethylases (erasers), respectively.^[^ ^15^, ^16^, ^17^, ^18^ ^]^ It has been demonstrated that the multiprotein writer complex and FIONA1 are responsible for writing m^6^A modifications in plants.^[^ ^19^, ^20^, ^21^, ^22^, ^23^ ^]^ The multiprotein writer complex inclu3des MTA (methyltransferase A), MTB (methyltransferase B), FIP37 (FKBP12 INTERACTING PROTEIN 37 KD ), VIR (VIRILIZER), HAKAI, and HAKAI‐interacting zinc figure protein (HIZ2).^[^ ^24^, ^25^, ^26^, ^27^, ^28^ ^]^ Recently, ALKBH9B (Alpha‐ketoglutarate‐dependent dioxygenase B (AlkB) homolog 9) and ALKBH10B (Alpha‐ketoglutarate‐dependent dioxygenase B (AlkB) homolog 10) have been shown to have demethylase activity in *Arabidopsis thaliana*.^[^ ^29^, ^30^ ^]^ In *Arabidopsis thaliana*, 13 proteins containing the YT521‐B homology (YTH) domain are responsible for binding m^6^A labeled RNAs.^[^ ^31^ ^]^ Previous studies have demonstrated that m^6^A can regulate multiple aspects of mRNA metabolism including mRNA stability, nuclear‐cytoplasmic export, and translation efficiency.^[^ ^32^, ^33^, ^34^ ^]^ In addition, m^6^A modification has been broadly involved in plant growth and development, biotic and abiotic stress responses.^[^ ^20^, ^24^, ^26^, ^27^, ^30^, ^35^, ^36^, ^37^, ^38^ ^]^ However, regulation of m^6^A machinery is so far mainly reported in animals and human cells, including the SUMOylation of METTL3 (methyltransferase‐like 3),^[^ ^39^ ^]^ YTHDF2 ((YT521‐B homology) domain 2),^[^ ^40^ ^]^ and ALKBH5 (Alpha‐ketoglutarate‐dependent dioxygenase B (AlkB) homolog 5),^[^ ^41^ ^]^ the acetylation of METTL3^[^ ^42^ ^]^ and ALKBH5,^[^ ^43^ ^]^ the phosphorylation and ubiquitination of METTL3.^[^ ^44^, ^45^ ^]^ Recently, the ubiquitin ligase STUB1 (STIP1 homology and U‐box‐containing protein 1) has been shown to interact with METTL3 and mediate the elvitegravir‐induced ubiquitination and degradation of METTL3 in human cells.^[^ ^45^ ^]^ In tomato, H_2_O_2_ can induce oxidative modification of the m^6^A RNA demethylase SlALKBH2 (Alpha‐ketoglutarate‐dependent dioxygenase B [AlkB] homolog 2) to form homodimers, promoting the stability of the SlALKBH2 protein, thereby playing a functional role during fruit ripening.^[^ ^46^ ^]^ Although one report suggests that H_2_O_2_ signaling integrates with m^6^A modification to coordinate plant development regulation, the role of m^6^A machinery in plants remains poorly understood.

So far, the importance of plant MTAs in the development of embryos and trichomes, fruit ripening, and abiotic stress response has been well addressed.^[^ ^24^, ^37^, ^47^, ^48^, ^49^, ^50^ ^]^ Our previous work demonstrates that MdMTA positively regulates drought tolerance by regulating the m^6^A levels of genes involved in the lignin metabolic process and oxidative stress.^[^ ^37^ ^]^ MTA promotes cold tolerance through regulating the translation efficiency of cold‐responsive genes in *Arabidopsis thaliana*.^[^ ^48^, ^49^ ^]^ Though the roles of plant MTAs are well described, how MTAs are regulated remains to be elucidated. Whether ubiquitin modification and degradation of MTA exist in plants is largely unclear.

Ubiquitin, a small protein consisting of 76‐amino acids (aa), covalently binds to cellular proteins via an enzyme cascade to mediate protein post‐translational regulation.^[^ ^51^ ^]^ The occurrence of ubiquitination depends on (E1) Ub‐activating enzymes, (E2) Ub‐conjugating enzymes, and (E3) Ub‐protein ligases.^[^ ^52^ ^]^ Meanwhile, the recognition and delivery of ubiquitinated proteins to the proteasome are dependent on ubiquitin‐like (UBL)‐ubiquitin‐associated (UBA) proteins that act as shuttling ubiquitin receptors.^[^ ^53^, ^54^ ^]^ DOMINANT SUPPRESSOR OF KAR 2 (DSK2), a UBL‐UBA protein, targets ubiquitinated proteins for degradation through proteasome and selective autophagy pathways in *Arabidopsis thaliana*.^[^ ^55^ ^]^ Despite explorations of ubiquitin receptor proteins involved in the ubiquitin‐proteasome system (UPS), very few studies on DSK2 have been reported in plants, which makes its function largely unknown in stress response and plant development. In *Arabidopsis thaliana*, DSK2 can not only be phosphorylated by BIN2 (BRASSINOSTEROID‐INSENSITIVE2) to promote its interaction with ATG8 (Autophagy‐Related 8) and target BES1 (BRI1‐EMS‐Suppressor 1) for degradation through the autophagy pathway, but form a complex with SINAT2 (SEVEN IN ABSENTIA OF ARABIDOPSIS2) to carry out BES1 degradation, thereby balancing growth and stress response under starvation and drought stress conditions.^[^ ^55^ ^]^ However, whether DSK2 is involved in the regulation of m^6^A machinery, m^6^A modification, and cold stress remains largely unknown.

In this study, we combined the transcriptome‐wide m^6^A profiling with RNA‐seq analysis and found that MdMTA‐mediated m^6^A modifications alter the expression of genes involved in the ROS scavenging and the deposition of cellulose and hemicellulose by regulating their mRNA stability under cold conditions. Phenotypic analysis showed that MdMTA is a positive regulator of freezing tolerance. We also found that MdMTA interacts with a ubiquitin receptor protein, MdDSK2a‐like, in vitro and in vivo. Interestingly, this interaction could cause the degradation of MdMTA through the 26S proteasome and selective autophagy pathways, and this degradation was alleviated under cold conditions. Further studies revealed that MdDSK2a‐like negatively regulates apple cold hardiness by reducing MdMTA level, thereby regulating the m^6^A levels of its downstream target genes. Overall, our work illustrates the regulation of MdMTA by MdDSK2a‐like and the roles of MdDSK2a‐like‐MdMTA in m^6^A modification and cold response in apple trees.

## 2 Results

### 2.1 m^6^A Methylation is a Common Feature of mRNA in Apple Response to Cold Stress

**Figure 1:**
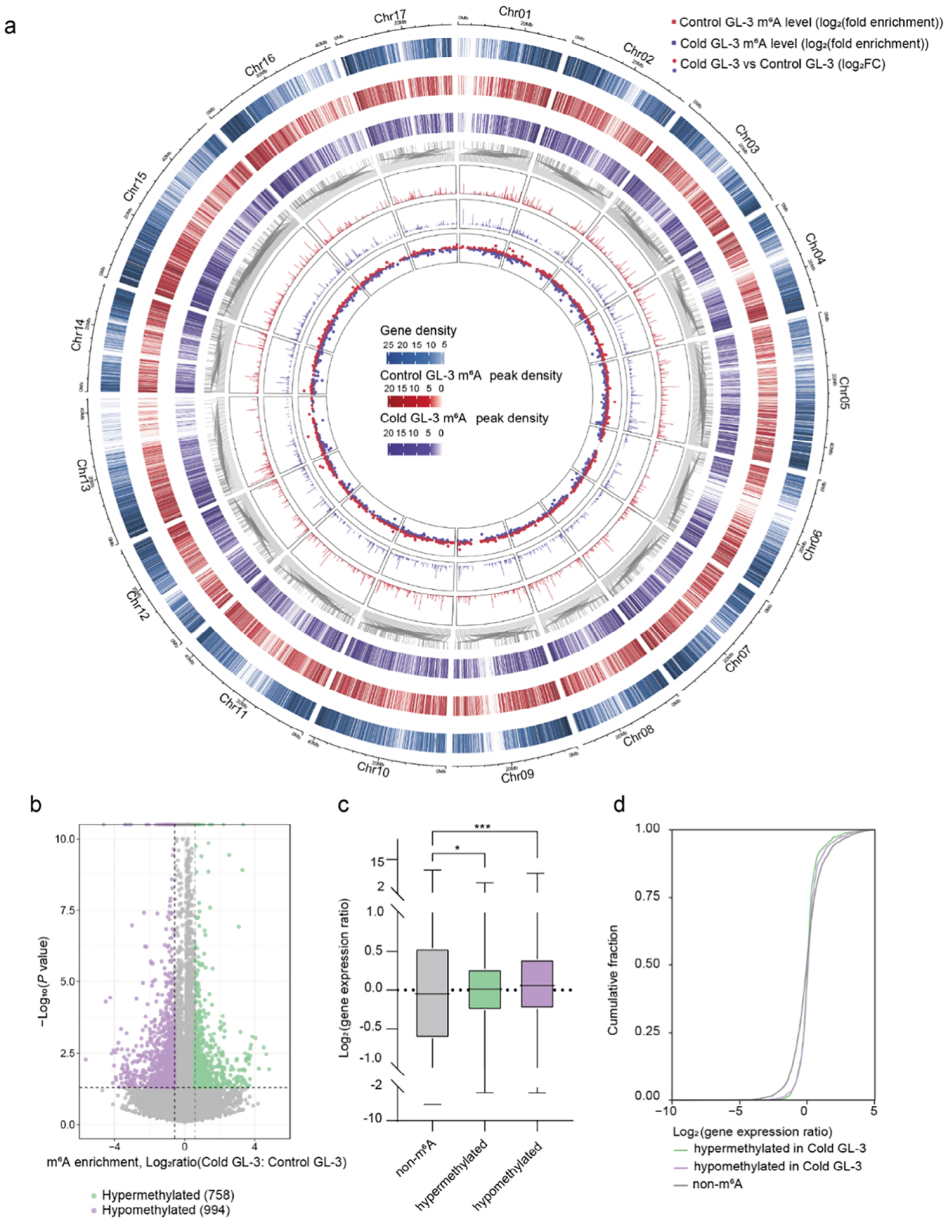
Effect of cold stress on m^6^A RNA methylome and transcriptome. a) Circle diagram illustrating the m^6^A modification pattern of GL‐3 under control and cold conditions. The eight rings from the outside to the inside show the chromosomes (1st), gene density (2nd), m^6^A peak density of GL‐3 under control conditions (3rd), m^6^A peak density of GL‐3 under cold conditions (4th), the position of differential peaks (Cold GL‐3 vs Control GL‐3) (5th), the log_2_(fold enrichment) of differential peaks in Control GL‐3 (6th), Cold GL‐3 (7th), and the log_2_(fold change) of differential peaks (8th). The red dot indicates hypermethylated peaks, and the blue dot indicates hypomethylated peaks in GL‐3 plants under cold conditions compared with control conditions. For estimation of the gene or m^6^A peak density of each 100 kb window, the number of genes or m^6^A peaks within the window was counted. b) Volcano plot showing the hypermethylated peaks (green) and hypomethylated peaks (purple) in GL‐3 plants under cold conditions compared to control conditions. c) Boxplot comparison of expression levels of transcript fragments containing the m^6^A and non‐m^6^A modifications in response to cold stress (*P < 0.05; ***P < 0.001; Wilcoxon test). d) Cumulative distribution of changes in gene expression in (c). Control GL‐3, 2‐month‐old GL‐3 plants were grown at 22 °C. Cold GL‐3, 2‐month‐old GL‐3 plants were treated at 0 °C for 10 h.

To investigate whether m^6^A methylation participates in the response of apple to cold stress, we detected m^6^A levels of GL‐3 plants (wild type) by LC‐MS/MS analysis under control and cold conditions. Result showed that the m^6^A level of GL‐3 after cold treatment was higher than that under control conditions (Figure S1, Supporting Information). To gain a more detailed insight into the m^6^A methylation in response to cold stress, we performed MeRIP‐seq (m^6^A‐seq) using GL‐3 plants. After adaptor trimming and reads filtering, a total of 30–35 million clean reads were obtained for each sample under cold stress, and ≈95% of these reads were uniquely aligned to the latest apple reference genome (Additional file 1: Table S1, Supporting Information). High Pearson correlation coefficients of MeRIP‐seq within biological replicates indicated reliable repeatability of our data (Figure S2, Supporting Information). We then used the 13,100 confident peaks that were consistently identified in all three biological replicates under cold stress for subsequent analysis (Figure S3, Supporting Information). Circos diagram showing gene density, m^6^A peak density, and m^6^A levels indicates that the m^6^A peak density is positively associated with the gene density of GL‐3 under cold stress (**Figure** **1**a).

To verify the accuracy of the MeRIP‐seq results, we randomly selected six genes and performed m^6^A‐immunoprecipitation (IP)‐qPCR analyses. Results showed that the m^6^A levels of *MD01G1085600*, *MD03G1084800, MD05G1351300*, and *MD10G1153500* were down‐regulated, while the m^6^A levels of *MD05G1092900* and *MD15G1096100* were up‐regulated in GL‐3 plants under cold conditions, consistent with the MeRIP‐seq results (Figure S4, Supporting Information).

Compared to GL‐3 grown under control conditions, a total of 758 hypermethylated m^6^A peaks and 994 hypomethylated m^6^A peaks, corresponding to 752 and 989 transcripts, were detected in GL‐3 under cold treatment, respectively (Figure 1b). Considering the accumulating evidence that m^6^A deposition affects mRNA abundance,^[^ ^56^ ^]^ we next performed RNA‐seq analysis (Figure S5, Supporting Information). Compared to GL‐3 under control conditions, 2,812 up‐regulated and 2085 down‐regulated genes were detected in cold‐treated GL‐3. Then we analyzed the gene expression alterations associated with m^6^A‐hypermethylated, m^6^A‐hypomethylated, and nondifferential m^6^A‐modified genes. As shown in Figure 1c, compared to GL‐3 under control conditions, genes in cold‐treated GL‐3 harboring both the m^6^A hypermethylation and hypomethylation exhibited significantly higher expression levels than the nondifferential m^6^A‐modified genes. Cumulative distribution analysis of gene expression alterations among hypermethylated, hypomethylated, and nondifferential m^6^A‐methylated genes showed the same results (Figure 1d). These results suggest that the relationship between m^6^A levels and expression levels is complicated.

### 2.2 MdMTA Regulates the m^6^A Levels and mRNA Stability of Transcripts Involved in ROS Scavenging and Cell Wall Deposition Under Cold Stress

**Figure 2:**
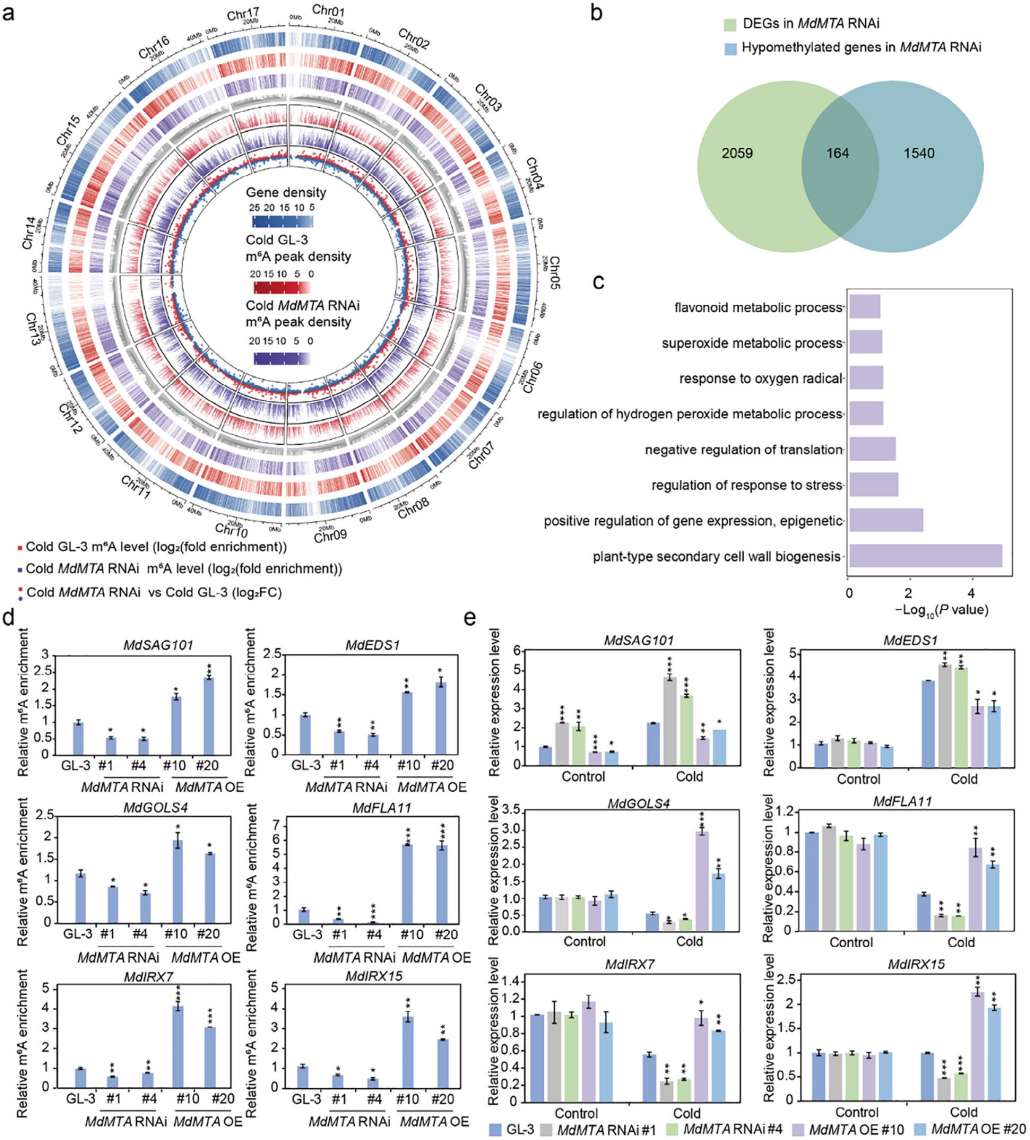
MdMTA regulates m^6^A levels of genes involved in ROS and secondary cell wall biogenesis. a) Circle diagram illustrating the m^6^A modification pattern of GL‐3 and *MdMTA* RNAi plants under cold conditions. The eight rings from the outside to the inside show the chromosomes (1st), gene density (2nd), m^6^A peak density of cold‐treated GL‐3 (3rd), m^6^A peak density of cold treated *MdMTA* RNAi plants (4th), the position of differential peaks (Cold *MdMTA* RNAi versus Cold GL‐3) (5th), the log_2_(fold enrichment) of differential peaks in Cold GL‐3 (6th), Cold *MdMTA* RNAi plants (7th), and the log_2_(fold change) of differential peaks (8th). The red dot indicates hypermethylated peaks, and the blue dot indicates hypomethylated peaks in *MdMTA* RNAi plants compared with GL‐3 plants under cold conditions. To estimate the gene or m^6^A peak density of each 100 kb window, the number of genes or m^6^A peaks in each window was counted. Cold *MdMTA* RNAi, 2‐month‐old *MdMTA* RNAi plants were treated at 0 °C for 10 h. b) Venn diagram showing the intersection of hypomethylated genes and differentially expressed genes (DEGs) in *MdMTA* RNAi plants compared with GL‐3 plants under cold conditions. c) Gene Ontology (GO) enrichment of the overlapped genes from the Venn diagram shown in (b). d) Validation of the m^6^A enrichment under cold conditions by m^6^A‐immunoprecipitation (IP) ‐qPCR. e) Transcript levels of genes determined by qRT‐PCR in GL‐3 and *MdMTA* transgenic plants under control and cold conditions. Two‐month‐old plants treated at 0 °C for 10 h were used for m^6^A‐IP‐qPCR and qRT‐PCR. The asterisks indicate significant differences between the GL‐3 and transgenic lines based on Tukey's test (*P < 0.05; **P < 0.01; ***P < 0.001). The error bars indicate standard deviations (*n*  =  3 in (d) and (e)).

Previously, we found that MdMTA, an apple methyltransferase, is required for m^6^A modification in apple.^[^ ^37^ ^]^ MdMTA accumulated as cold treatment was extended (Figure S6¸ Supporting Information), suggesting that MdMTA can be used to explore the role of m^6^A in apple cold response. We performed MeRIP‐seq and RNA‐seq using GL‐3 and *MdMTA* RNAi (#1) plants that we had previously generated.^[^ ^37^ ^]^ For each treatment, we used three highly replicable independent biological replicates (Figure S2,S5, Supporting Information). From MeRIP‐seq analysis, we obtained 11669 confident peaks encoding 10 005 transcripts in *MdMTA* RNAi plants under cold stress (Figure S7, Supporting Information). To study the potential m^6^A methylation targets of MdMTA under cold stress, we analyzed the differential m^6^A peaks between GL‐3 and *MdMTA* RNAi plants under cold conditions. The Circus diagrams showed that interference with *MdMTA* did not affect peak density, but affected the m^6^A levels of some transcripts under cold conditions (**Figure** **2**a). Under cold stress, a total of 164 hypomethylated transcripts displayed differential expression between *MdMTA* RNAi and GL‐3 plants (Figure 2b). GO (Gene Ontology) enrichment analysis showed that some of these 164 transcripts are involved in cold response‐related pathways, including H_2_O_2_ metabolic process, superoxide metabolic process, and plant‐type secondary cell wall biogenesis (Figure 2c). Among the hypomethylated DEGs (Differentially Expressed Genes) in *MdMTA* RNAi plants under cold stress, homologs of *MdSAG101* (*SENESCENCE‐ASSOCIATED GENE101*) and *MdEDS1* (*ENHANCED DISEASE SUSCEPTIBILITY1*) are negative regulators of cold tolerance by attenuating H_2_O_2_ scavenging,^[^ ^57^ ^]^ while homolog of *MdGOLS4* (*Galactinol synthase 4*) is a positive regulator of cold tolerance by promoting ROS scavenging.^[^ ^58^ ^]^ Homologs of *MdFLA11* (*fasciclin‐like arabinogalactan‐protein 11), MdIRX7* (*IRREGULAR XYLEM 7*), and *MdIRX15* (*IRREGULAR XYLEM 15*) are related to secondary cell wall biogenesis.^[^ ^59^, ^60^, ^61^, ^62^ ^]^ m^6^A‐IP‐qPCR results showed that the m^6^A levels of *MdSAG101, MdEDS1, MdGOLS4, MdFLA11, MdIRX7*, and *MdIRX15* were reduced in *MdMTA* RNAi plants under cold conditions, as compared with GL‐3 plants, which is consistent with the MeRIP‐seq results (Figure 2b and Figure S8, Supporting Information). On the contrary, the m^6^A levels of these genes in *MdMTA* OE plants were higher than those in GL‐3 plants under cold conditions (Figure 2d). Under control conditions, MdMTA did not regulate the m^6^A levels of the above genes (Figure S9, Supporting Information). These results suggest that MdMTA regulates the m^6^A levels of transcripts involved in ROS scavenging and cell wall deposition under cold stress.

m^6^A modification often regulates gene expression in different physiological processes,^[^ ^56^ ^]^ we then analyzed the expression levels of *MdSAG101, MdEDS1, MdGOLS4, MdFLA11, MdIRX7*, and *MdIRX15* in *MdMTA* transgenic plants and GL‐3 plants. Results showed that the expression levels of *MdSAG101* and *MdEDS1* were up‐regulated in *MdMTA* RNAi plants but down‐regulated in *MdMTA* OE plants under cold stress (Figure 2e). The expression levels of *MdGOLS4, MdFLA11, MdIRX7*, and *MdIRX15* were down‐regulated in *MdMTA* RNAi plants but up‐regulated in *MdMTA* OE plants under cold stress (Figure 2e). These results suggest that MdMTA may affect the expression of these genes by regulating their m^6^A levels under cold conditions, and this regulation is not simply in a positive or negative way.

**Figure 3:**
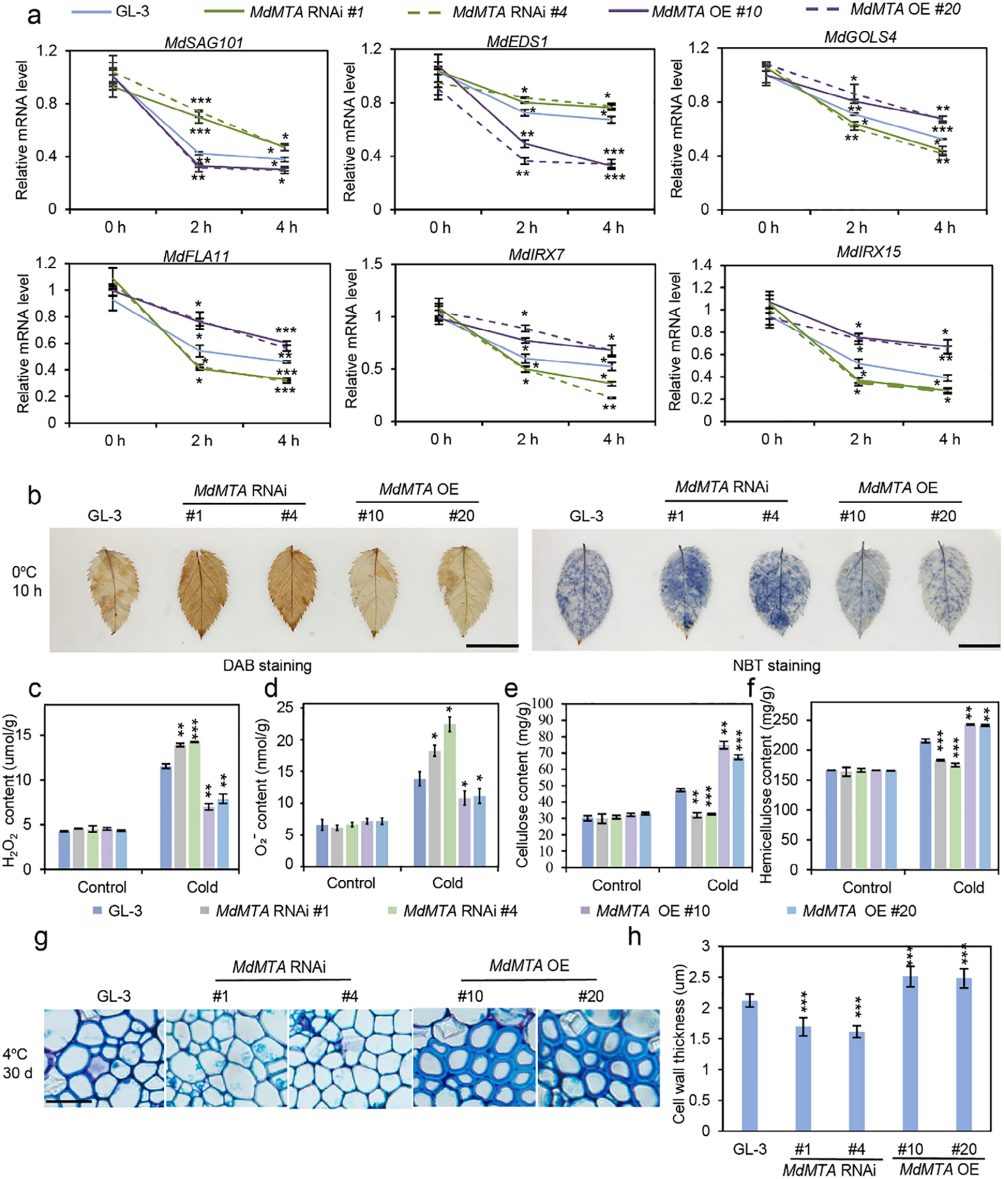
MdMTA regulates the mRNA stability of genes involved in ROS detoxification and deposition of cellulose and hemicellulose. a) The degradation rate of the transcripts involved in ROS detoxification and deposition of cellulose and hemicellulose in GL‐3 and *MdMTA* transgenic plants under cold conditions. Two‐month‐old plants were treated with 10 µm actinomycin D for 0, 2, and 4 h at 0 °C. Samples at different times were collected for qRT‐PCR. b) DAB and NBT staining of GL‐3 and *MdMTA* transgenic plants at 0 °C for 10 h. Bars = 3 cm. c) Hydrogen peroxide (H_2_O_2_) and d) superoxide anion (O_2_^−^) content in GL‐3 and *MdMTA* transgenic plants treated at 0 °C for 10 h. e) Cellulose and f) hemicellulose content in GL‐3 and *MdMTA* transgenic plants treated at 4 °C for 1 month. g) Toluidine blue staining of GL‐3 and *MdMTA* transgenic plants treated at 4 °C for 1 month. Bar = 20 µm. h) Cell wall thickness of apple leaves. Cell wall thickness was measured using ImageJ software. Two‐month‐old plants were used for DAB, NBT, and toluidine blue staining, as well as for the detection of hydrogen peroxide, superoxide anion, cellulose and hemicellulose content. DAB, 3, 3′‐diaminobenzidine; NBT, nitroblue tetrazolium. The asterisks indicate significant differences between the GL‐3 and transgenic lines based on Tukey's test (*P < 0.05; **P < 0.01; ***P < 0.001). The error bars indicate standard deviations (*n* = 3 in (a, c–f); *n* = 7 in (h)).

Current studies have shown that the relationship between m^6^A modification and mRNA stability is intricate.^[^ ^63^, ^64^ ^]^ To study the effect of m^6^A modification on mRNA stability under cold conditions, we used actinomycin D to measure the lifetime of the transcripts shown in Figure 2d,e. Results showed that *MdSAG101* and *MdEDS1* transcripts were degraded more slowly, but *MdGOLS4, MdFLA11, MdIRX7*, and *MdIRX15* transcripts were degraded more quickly in *MdMTA* RNAi plants than in GL‐3 plants under cold conditions (**Figure** **3**a). On the contrary, *MdMTA* OE plants had less stable transcripts of *MdSAG101* and *MdEDS1* but more stable transcripts of *MdGOLS4, MdFLA11, MdIRX7*, and *MdIRX15* under cold stress as compared with GL‐3 plants (Figure 3a). However, MdMTA did not regulate the degradation of these transcripts under control conditions (Figure S10, Supporting Information). Taken together, the above results collectively demonstrate that MdMTA‐mediated m^6^A modification regulates gene expression through affecting the transcripts’ stability.

In *Arabidopsis thaliana*, *sag101*, and *eds1* mutant plants enhance their cold tolerance by increasing *CBFs* (*C‐repeat binding factor*) expression and reducing H_2_O_2_ accumulation.^[^ ^57^ ^]^ In cucumber, overexpression of *GOLS4* can increase the raffinose oligosaccharides (RFO) content and promote ROS scavenging, thus improving the drought and cold tolerance.^[^ ^58^ ^]^ Since MdMTA can regulate the m^6^A levels of *MdSAG101, MdEDS1*, and *MdGOLS4* to regulate their expression levels, we speculated that MdMTA promotes ROS scavenging. 3, 3′‐diaminobenzidine (DAB) and nitroblue tetrazolium (NBT) staining revealed that *MdMTA* RNAi plants accumulated more H_2_O_2_ and O_2_^−^ content than GL‐3 plants, whereas *MdMTA* OE plants had less H_2_O_2_ and O_2_^−^ content under cold conditions (Figure 3b). Under control conditions, no significant difference in H_2_O_2_ and O_2_^−^ content between *MdMTA* transgenic plants and GL‐3 plants was observed (Figure S12a,b, Supporting Information). Consistently, H_2_O_2_ and O_2_^−^ measurements showed that MdMTA positively regulated the scavenging of H_2_O_2_ and O_2_^−^ under cold stress (Figure 3c,d). Additionally, we detected the CAT and POD enzyme activities involved in the antioxidant defense system.^[^ ^65^ ^]^ Under cold stress, the enzyme activities were lower in *MdMTA* RNAi plants but higher in *MdMTA* OE plants, as compared to those in GL‐3 plants (Figure S11, Supporting Information). These data suggest that MdMTA positively regulates apple cold hardiness, at least in part, by promoting ROS scavenging.

Composed of pectin, cellulose, and hemicellulose, plant cell wall plays an important role in abiotic stress tolerance, including cold stress tolerance.^[^ ^9^, ^66^ ^]^ In *Arabidopsis thaliana*, *fla11* has lower cellulose content than the wild type.^[^ ^67^ ^]^ Xylan is the main component of hemicellulose in the secondary cell wall of dicotyledonous plants.^[^ ^68^ ^]^ In *Arabidopsis thaliana, irx7*, and *irx15 irx15l* double mutants have less xylan content than wild type.^[^ ^60^, ^61^ ^]^ Since MdMTA regulated the m^6^A levels and expression levels of *MdFLA11, MdIRX7*, and *MdIRX15*, we therefore detected the content of cellulose and hemicellulose in *MdMTA* transgenic plants. We found that *MdMTA* RNAi plants had lower cellulose and hemicellulose content than GL‐3 plants, whereas *MdMTA* OE plants had higher content under cold conditions (Figure 3e,f). To observe the cell wall deposition, we used toluidine blue to stain apple leaves before and after cold treatment. The results showed that under cold conditions the cell wall of *MdMTA* RNAi plants was thinner than that of GL‐3 plants, while the cell wall of *MdMTA* OE plants was thicker (Figure 3g,h). Under control conditions, the cell wall thickness of *MdMTA* transgenic plants was comparable to that of GL‐3 plants (Figure S12c,d, Supporting Information). These data suggest that MdMTA regulates cell wall deposition under cold conditions, which might contribute to apple cold hardiness.

### 2.3 MdMTA Plays a Positive Role in Apple Cold‐Hardiness

**Figure 4:**
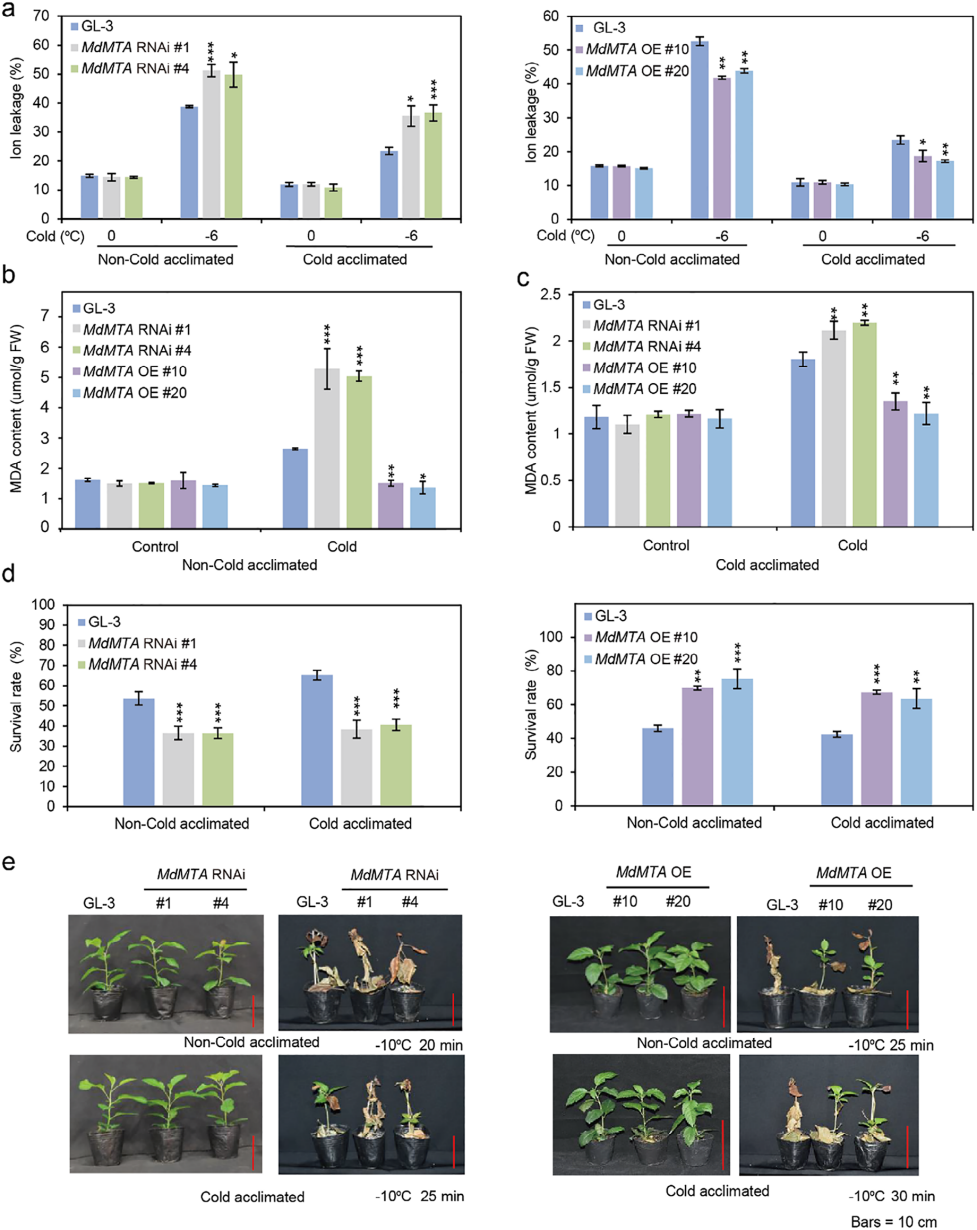
MdMTA promotes cold hardiness in apple. a) Leaf electrolyte leakage detection of GL‐3 and *MdMTA* transgenic plants under cold conditions. b) MDA content of *MdMTA* transgenic plants under non‐cold and c) cold acclimation conditions. Under non‐cold acclimation, 2‐month‐old plants treated at 4 °C for 10 h were used for MDA content detection. Under cold acclimation, 2‐month‐old plants treated at 0 °C for 10 h were used for MDA content detection. d) Survival rates of *MdMTA* transgenic plants before or after cold acclimation. e) Whole‐plant cold tolerance of *MdMTA* transgenic plants shown in (d). Bars = 10 cm. Two‐month‐old plants with or without cold acclimation were treated at −10 °C and then recovered under control conditions for 1 month. Under non‐cold acclimation, *MdMTA* RNAi plants were treated at −10 °C for 20 min, and *MdMTA* OE plants were treated for 25 min. Under cold acclimation, *MdMTA* RNAi plants were treated at −10 °C for 25 min, and *MdMTA* OE plants were treated for 30 min. The asterisks indicate significant differences between the GL‐3 and transgenic lines based on Tukey's test (*P < 0.05; **P < 0.01; ***P < 0.001). The error bars indicate standard deviations (*n*  =  6 in (a); 3 in (b–d)).

We next analyzed the phenotype of *MdMTA* transgenic plants under cold stress. Compared with GL‐3 plants, *MdMTA* RNAi plants had higher electrolyte leakage under freezing stress, whereas *MdMTA* OE plants had lower electrolyte leakage, either with or without cold acclimation (**Figure** **4**a). These results suggest more severe leaf membrane damage of *MdMTA* RNAi plants but more intact leaf membrane of *MdMTA* OE plants under freezing stress, as compared to GL‐3 plants. Besides electrolyte leakage, MDA (Malondialdehyde) content can directly or indirectly indicate the degree of membrane system damage.^[^ ^69^ ^]^ Compared with GL‐3 plants, *MdMTA* RNAi plants had higher MDA content, whereas *MdMTA* OE plants had lower MDA content under non‐cold‐acclimation conditions (Figure 4b). In addition, we also measured the MDA content of *MdMTA* transgenic plants after cold acclimation. Compared with GL‐3 plants, *MdMTA* RNAi plants had higher MDA content, whereas *MdMTA* OE plants had lower MDA content after cold acclimation (Figure 4c). Therefore, non‐cold‐acclimated and cold‐acclimated *MdMTA* transgenic plants displayed a similar pattern in MDA accumulation, as compared to GL‐3 plants. Besides, after freezing stress, fewer *MdMTA* RNAi plants survived compared to GL‐3 plants, either after cold acclimation or without cold acclimation (Figure 4d,e). On the contrary, both cold‐acclimated and non‐cold‐acclimated *MdMTA* OE plants had higher survival rates than GL‐3 plants after freezing stress (Figure 4d,e). The above results suggest that MdMTA positively regulates freezing tolerance in apple.

### 2.4 MdDSK2a‐Like is Involved in the Recognition of MdMTA and Its Degradation through the 26S Proteasome and Autophagy Pathway

**Figure 5:**
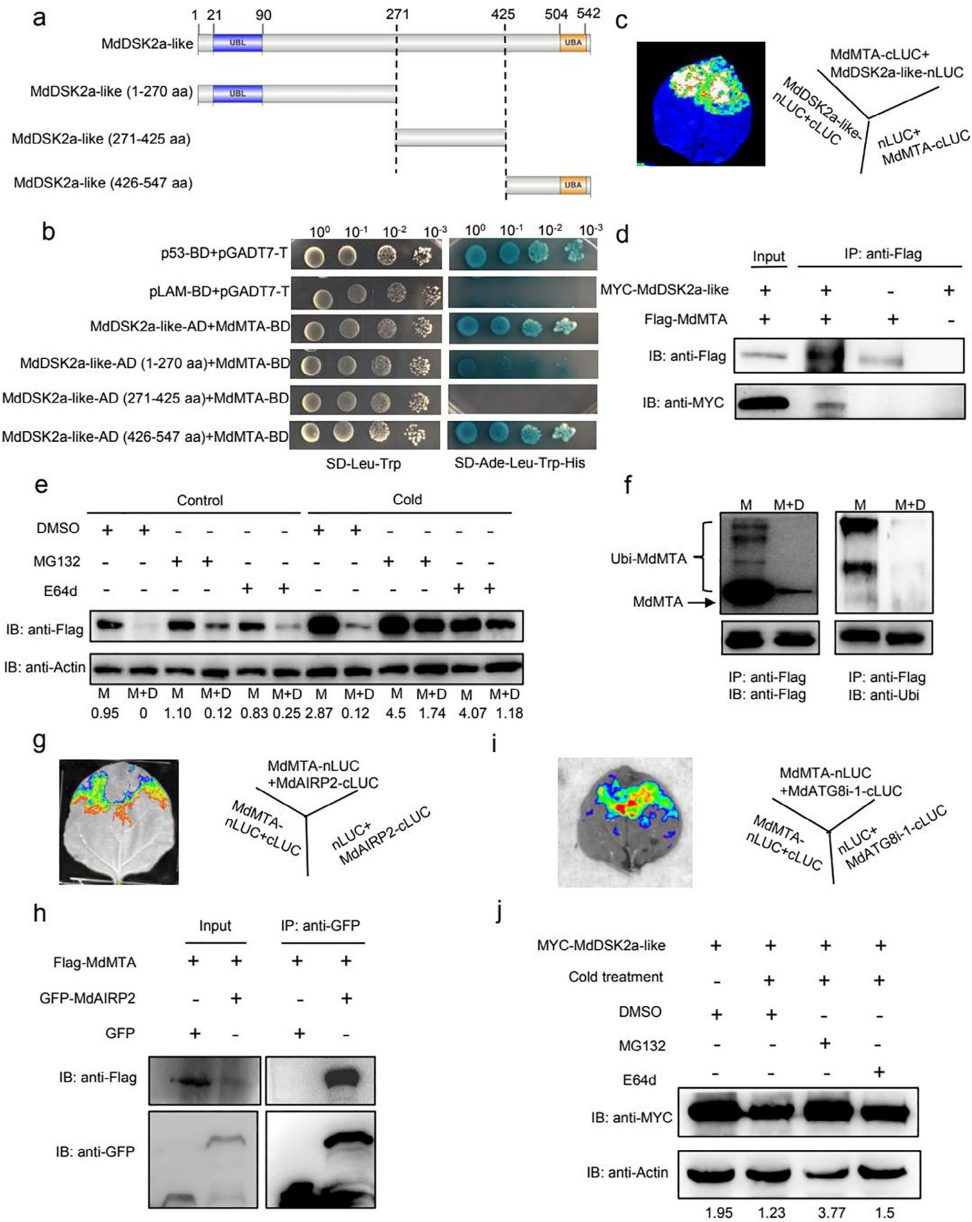
MdMTA interacts with and is degraded by the ubiquitin receptor protein MdDSK2a‐like via the 26S proteasome and autophagy pathways. a) Domain architecture of MdDSK2a‐like protein structure. Blue block denotes UBL domain and orange block denotes UBA domain. b) Interaction of MdMTA with MdDSK2a‐like detected by yeast two‐hybrid. c) Split‐LUC analysis showing the interaction of MdMTA and MdDSK2a‐like. d) Coimmunoprecipitation in *N. benthamiana* showing the association of MdMTA with MdDSK2a‐like. Total proteins were extracted and immunoprecipitation was performed with anti‐Flag overnight. Western blot was performed with anti‐Flag or anti‐MYC antibodies. e) The degradation of MdMTA mediated by MdDSK2a‐like in apple calli under control and cold conditions. *35S:MdDSK2a‐like‐MYC* was transformed stably by *Agrobacterium*‐mediated transformation into the apple wild‐type calli (*MdDSK2a‐like* OE/WT) or transgenic calli overexpressing *MdMTA‐Flag* (*MdDSK2a‐like* OE/*MdMTA* OE). The transgenic calli were cultured with MS liquid medium containing dimethyl sulfoxide (DMSO), 50 µm MG132, or 20 µm E64d for 12 h at 22 and 0 °C, then MdMTA total proteins were analyzed by western blot with anti‐Flag antibody. Actin served as the loading control. MG132, proteasome inhibitor; E64d functions as an inhibitor that impedes autophagy degradation. M: *MdMTA* OE calli; M+D: *MdDSK2a‐like* OE/*MdMTA* OE calli. f) The ubiquitination of MdMTA mediated by MdDSK2a‐like in apple calli. Total proteins were extracted from *MdMTA* OE or *MdDSK2a‐like* OE/*MdMTA* OE transgenic calli and immunoprecipitated with anti‐Flag antibody, then western blot was performed with anti‐Flag or anti‐Ubi (Ubiquitin) antibodies. g) Split‐LUC analysis showing the interaction of MdMTA and MdAIRP2. h) Coimmunoprecipitation in *N. benthamiana* showing the association of MdMTA with MdAIRP2. Total proteins were extracted and immunoprecipitation was performed with anti‐GFP overnight. Western blot was performed with anti‐GFP or anti‐Flag antibodies. i) Split‐LUC analysis showing the interaction of MdMTA and MdATG8i‐1. j) The degradation of MdDSK2a‐like under cold conditions. *MYC‐MdDSK2a‐like* was expressed in *N. benthamiana* leaves, which was then treated at 0 °C for 4 h. Total proteins were extracted, followed by western blot analysis by anti‐MYC or anti‐Actin antibodies.

To further explore the molecular function of MdMTA in apple cold response, we performed yeast two‐hybrid (Y2H) screening using MdMTA as the bait and identified MdDSK2a‐like, a ubiquitin‐binding receptor protein (**Figure** **5**b; and Figure S13, Supporting Information, Table S2, Supporting Information). Tissue‐specific expression analysis showed that the expression level of *MdDSK2a‐like* in stems was significantly higher than that in leaves and roots (Figure S14a, Supporting Information). Subcellular localization analysis showed that MdDSK2a‐like was localized in the plasma membrane, cytoplasm, and nucleus (Figure S14b, Supporting Information). Besides, cold treatment had no effect on the subcellular localization of MdDSK2a‐like (Figure S14b, Supporting Information). Using Y2H, Split‐luciferase (Split‐LUC), and Coimmunoprecipitation (Co‐IP) analysis, we confirmed that MdMTA can interact with MdDSK2a‐like in vitro and in vivo (Figure 5a–d). Using the truncated MdDSK2a‐like protein, we found that MdMTA not only interacted with the UBL domain in the MdDSK2a‐like N‐terminal but also the UBA domain in its C‐terminal (Figure 5a,b). As a transporter protein, DSK2 digests ubiquitinated proteins through proteasome and autophagy pathways.^[^ ^55^ ^]^ Therefore, we examined whether MdDSK2a‐like is involved in MdMTA degradation. First, we obtained transgenic apple calli overexpressing *MdMTA* only (*MdMTA* OE) or overexpressing *MdDSK2a‐like* in *MdMTA* OE transgenic calli (*MdDSK2a‐like* OE*/MdMTA* OE) (Figure S15a–f, Supporting Information), and examined MdMTA protein level in transgenic calli. Results showed that the MdMTA protein level in *MdDSK2a‐like* OE*/MdMTA* OE calli was almost undetectable under control conditions, indicating a thorough degradation of MdMTA by MdDSK2a‐like in apple calli (Figure 5e). However, once proteasome inhibitor MG132 or autophagy inhibitor E64d was added, MdMTA protein level could be partially recovered in the *MdDSK2a‐like* OE*/MdMTA* OE calli under control conditions, indicating that MdDSK2a‐like promotes MdMTA degradation through the 26S proteasome and autophagy pathways (Figure 5e). We also detected the MdMTA degradation targeted by MdDSK2a‐like under cold stress. The results show that MdMTA degradation has a similar trend to that under control conditions; however, the degradation is weakened under cold stress compared with the control conditions, as MdMTA level significantly increased in all the samples we detected (Figure 5e).

Next, we analyzed the ubiquitin level of MdMTA in the *MdMTA* OE and *MdDSK2a‐like* OE*/MdMTA* OE calli. In the presence of MdDSK2a‐like, the ubiquitin level of MdMTA decreased (Figure 5f), suggesting that MdDSK2a‐like accelerated the degradation process of ubiquitinated MdMTA. To explore how MdMTA protein is degraded through the 26S proteasome pathway, we screened E3 ubiquitin‐protein ligases in the Y2H library screening results of MdMTA and identified an E3 ubiquitin protein, MdAIRP2 (ABA‐insensitive RING protein 2) (Table S2, Supporting Information). Unfortunately, MdMTA did not interact with MdAIRP2 in yeast (Figure S13, Supporting Information). Then, we detected the interaction between MdMTA and MdAIRP2 in vivo. Split‐LUC and Co‐IP results showed MdMTA could interact with MdAIRP2 (Figure 5g,h). In *Arabidopsis thaliana*, DSK2 can interact with ATG8a, ATG8e, ATG8f, and ATG8i to degrade BES1 through autophagy pathway.^[^ ^55^ ^]^ In order to clarify how MdDSK2a‐like was involved in targeting MdMTA for degradation through the autophagy pathway, we examined the interactions between MdDSK2a‐like or MdMTA and MdATG8s using AlphaFold.^[^ ^70^ ^]^ Based on AlphaFold predictions, neither MdDSK2a‐like nor MdMTA directly interacted with any MdATG8s (Figure S16, Supporting Information). We also performed Y2H assay to examine the interaction between MdDSK2a‐like or MdMTA and MdATG8s. Unfortunately, the Y2H assay also showed that neither MdDSK2a‐like nor MdMTA directly interacts with MdATG8s (Figure S17,S18a, Supporting Information). Considering that the MdATG8 family members are highly conserved among themselves, we selected MdATG8i‐1 as a representative of MdATG8 family members and examined its interaction with MdDSK2a‐like or MdMTA in vivo. Split‐LUC and Co‐IP assays results suggested that either MdDSK2a‐like or MdMTA could interact with MdATG8i‐1 in vivo (Figure 5i and Figure S18b–d, Supporting Information). These data indicate that MdMTA protein might be degraded by MdAIRP2 through the 26S proteasome pathway and by MdATG8i‐1 through the autophagy pathway.

To understand the reduced degradation of MdMTA targeted by MdDSK2a‐like under cold conditions, we detected the protein level of MdDSK2a‐like under cold stress. These results show that MdDSK2a‐like protein is degraded after cold treatment, and its degradation under cold stress is through the 26S proteasome pathway, which is consistent with the accumulation of MdMTA under cold conditions (Figure 5j).

### 2.5 MdDSK2a‐Like Mediates the m^6^A Levels of mRNA Involved in ROS Scavenging and Cell Wall Deposition

**Figure 6:**
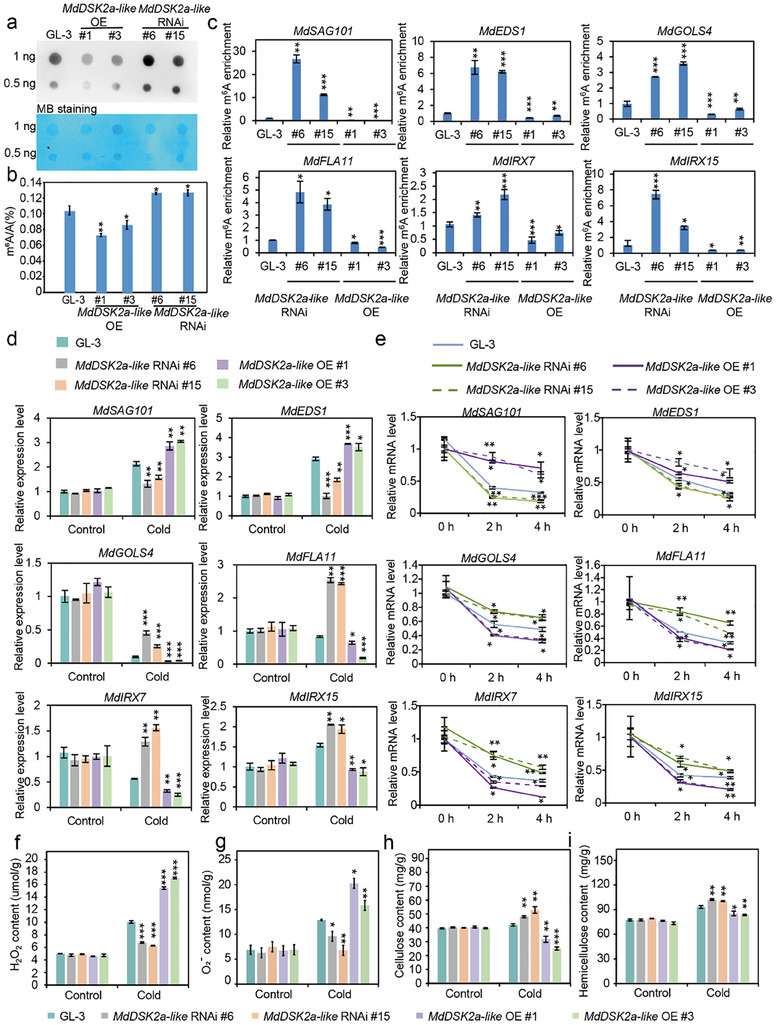
MdDSK2a‐like mediates m^6^A levels and stability of mRNAs involved in ROS detoxification and deposition of cellulose and hemicellulose. a) m^6^A signal in GL‐3 and *MdDSK2a‐like* transgenic plants treated at 0 °C for 10 h. MB staining: Methylene blue staining of RNA. b) m^6^A levels in GL‐3 and *MdDSK2a‐like* transgenic plants detected by LC‐MS/MS. c) m^6^A enrichment validation of cold‐responsive genes involved in ROS scavenging and the deposition of cellulose and hemicellulose in *MdDSK2a‐like* transgenic plants under cold conditions. d) Transcript levels of genes determined by qRT‐PCR in GL‐3 and *MdDSK2a‐like* transgenic plants under control and cold conditions. Two‐month‐old plants treated at 0 °C for 10 h were used for m^6^A level detection, m^6^A‐IP‐qPCR, and qRT‐PCR. e) The degradation rate of the transcripts in GL‐3 and *MdDSK2a‐like* transgenic plants under cold conditions. Two‐month‐old plants were treated with 10 µm actinomycin D at 0 °C for 0, 2, and 4 h. Samples with different treatment times were collected for qRT‐PCR. f) Hydrogen peroxide (H_2_O_2_) and g) superoxide anion (O_2_^−^) content in GL‐3 and *MdDSK2a‐like* transgenic plants treated at 0 °C for 10 h. h) Cellulose and i) hemicellulose content in GL‐3 and *MdDSK2a‐like* transgenic plants under cold conditions. Two‐month‐old plants were treated at 4 °C for 1 month and then used to detect the cellulose and hemicellulose content. The asterisks indicate significant differences between the GL‐3 and transgenic lines based on Tukey's test (*P < 0.05; **P < 0.01; ***P < 0.001). The error bars indicate standard deviations (*n*  =  3 in (b–i)).

Considering the degradation of MdDSK2a‐like on MdMTA, we speculated that MdDSK2a‐like could indirectly regulate the m^6^A level. Therefore, we generated transgenic apple plants that overexpressed or interfered with *MdDSK2a‐like* (Figure S19, Supporting Information), and detected the m^6^A levels of *MdDSK2a‐like* transgenic plants by dot‐blot and LC‐MS assay under control and cold conditions (**Figure** **6**a,b and Figure S20, Supporting Information). The results show that MdDSK2a‐like negatively regulates m^6^A levels under control and cold conditions, indicating that MdDSK2a‐like may regulate m^6^A levels in response to cold stress, and this regulation might be through its degradation of MdMTA.

Since MdMTA can regulate the expression levels and mRNA stability of genes involved in ROS detoxification and cell wall deposition through modulating the m^6^A levels of these transcripts under cold stress, we wondered if MdDSK2a‐like could play a similar role to MdMTA. We first detected the m^6^A levels of mRNA including *MdSAG101, MdEDS1, MdGOLS4, MdFLA11, MdIRX7*, and *MdIRX15* in *MdDSK2a‐like* transgenic plants under control and cold conditions. m^6^A‐IP‐qPCR results showed that their m^6^A levels were significantly increased in *MdDSK2a‐like* RNAi plants but reduced in *MdDSK2a‐like* OE plants under cold stress (Figure 6c), as compared with GL‐3 plants. However, their m^6^A levels were comparable in *MdDSK2a‐like* transgenic plants and GL‐3 plants under control conditions (Figure S21, Supporting Information). We then analyzed the expression of these genes in GL‐3 and *MdDSK2a‐like* transgenic plants in response to cold stress. qRT‐PCR analysis suggested that the expression levels of *MdSAG101* and *MdEDS1*, two negative regulators of cold tolerance, were down‐regulated in *MdDSK2a‐like* RNAi plants but up‐regulated in *MdDSK2a‐like* OE plants under cold conditions, as compared with GL‐3 plants. The expression levels of cold positive regulators *MdGOLS4, MdFLA11, MdIRX7*, and *MdIRX15* were up‐regulated in *MdDSK2a‐like* RNAi plants but down‐regulated in *MdDSK2a‐like* OE plants under cold conditions, when compared with GL‐3 plants (Figure 6d). We also examined the mRNA stability of these transcripts in *MdDSK2a‐like* transgenic plants under cold conditions. We found that the mRNA stability of *MdSAG101* and *MdEDS1* transcripts in *MdDSK2a‐like* RNAi plants was lower than that in GL‐3 plants, while that in *MdDSK2a‐like* OE plants was higher. Compared with GL‐3 plants, the mRNA stability of *MdGOLS4, MdFLA11, MdIRX7*, and *MdIRX15* transcripts was higher in *MdDSK2a‐like* RNAi plants, but lower in *MdDSK2a‐like* OE plants (Figure 6e). However, MdDSK2a‐like did not affect the mRNA stability of the above genes under control conditions (Figure S22, Supporting Information). Taken together, these results suggest that MdDSK2a‐like indirectly regulates the m^6^A levels of these genes by degrading MdMTA, which in turn affects their mRNA stability and leads to changes in expression levels under cold conditions.

We next measured the H_2_O_2_, O_2_^−^, cellulose, and hemicellulose content in GL‐3 and *MdDSK2a‐like* transgenic plants under control and cold conditions. Under control conditions, the content of H_2_O_2_ and O_2_^−^ showed no difference in *MdDSK2a‐like* transgenic plants compared with GL‐3 plants. After cold treatment, the content of H_2_O_2_ and O_2_^−^ in *MdDSK2a‐like* RNAi plants were lower than those in GL‐3 plants, but higher in *MdDSK2a‐like* OE plants (Figure 6f,g). DAB and NBT staining results were consistent with the H_2_O_2_ and O_2_^−^ detection results (Figure S23a, Supporting Information). The POD and CAT enzyme activities of *MdDSK2a‐like* transgenic plants showed opposite trend with H_2_O_2_ and O_2_^−^ content under cold stress (Figure S23b,c, Supporting Information). There was no significant difference in cellulose and hemicellulose content between *MdDSK2a‐like* transgenic plants and GL‐3 plants under control conditions; however, the content of cellulose and hemicellulose was higher in *MdDSK2a‐like* RNAi plants but lower in *MdDSK2a‐like* OE plants than GL‐3 plants under cold conditions (Figure 6h,i). Consistently, toluidine blue staining results showed similar pattern with the cellulose and hemicellulose detection results (Figure S23d–f, Supporting Information). These results suggest that MdDSK2a‐like negatively regulates apple cold tolerance by inhibiting ROS scavenging and cell wall deposition.

### 2.6 MdDSK2a‐Like Negatively Regulates Cold Hardiness in Apple

**Figure 7:**
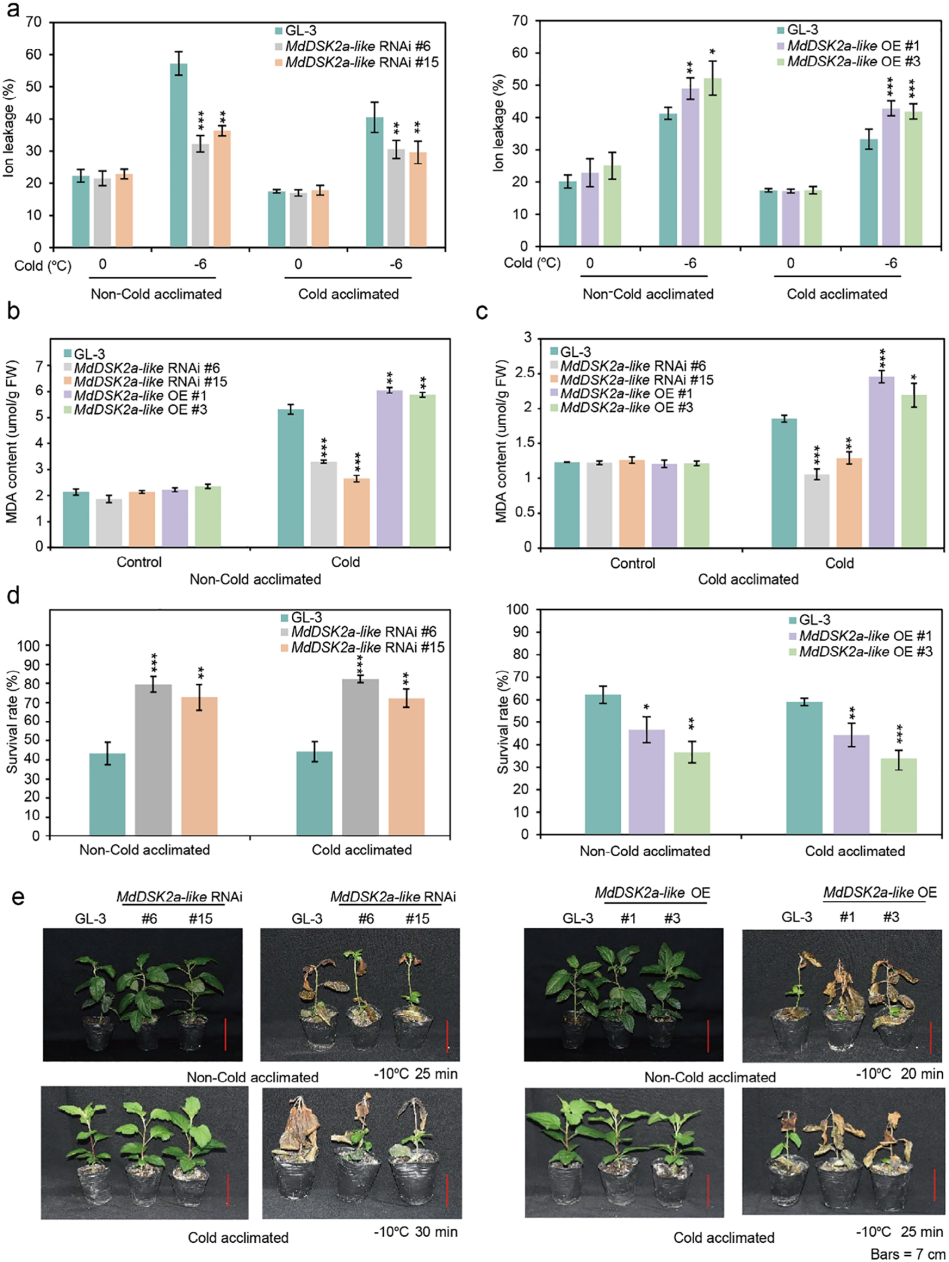
MdDSK2a‐like negatively regulates apple cold hardiness. a) Leaf electrolyte leakage of GL‐3 and *MdDSK2a‐like* transgenic plants under cold stress. b) MDA content of GL‐3 and *MdDSK2a‐like* transgenic plants under non‐cold and c) cold acclimation conditions. Under non‐cold acclimation, 2‐month‐old plants treated at 4 °C for 10 h were used for MDA content detection. Under cold acclimation, 2‐month‐old plants treated at 0 °C for 10 h were used for MDA content detection. d) Survival rates of GL‐3 and *MdDSK2a‐like* transgenic plants before or after cold acclimation. e) Whole‐plant cold tolerance of *MdDSK2a‐like* transgenic plants before or after cold acclimation. Bars = 7 cm. Two‐month‐old plants with or without cold acclimation were treated at −10 °C and then recovered under control conditions for 1 month. Under non‐cold acclimation, *MdDSK2a‐like* RNAi plants were treated at −10 °C for 25 min, and *MdDSK2a‐like* OE plants were treated for 20 min. Under cold acclimation, *MdDSK2a‐like* RNAi plants were treated at −10 °C for 30 min, and *MdDSK2a‐like* OE plants were treated for 25 min. The asterisks indicate significant differences between the GL‐3 and transgenic lines based on Tukey's test (*P < 0.05; **P < 0.01; ***P < 0.001). The error bars indicate standard deviations (*n*  =  6 in (a), 3 in (b–d)).

To study the biological function of MdDSK2a‐like under cold stress, we detected the electrolyte leakage of *MdDSK2a‐like* transgenic plants. The results showed that the electrolyte leakage of *MdDSK2a‐like* RNAi plants under freezing stress was lower than that of GL‐3, whereas *MdDSK2a‐like* OE plants had higher electrolyte leakage with or without cold acclimation (**Figure** **7**a). We also measured MDA content in *MdDSK2a‐like* transgenic plants. *MdDSK2a‐like* RNAi plants had lower MDA content, but *MdDSK2a‐like* OE plants had higher MDA content than GL‐3 plants under non‐cold‐acclimated conditions (Figure 7b). After cold acclimation, compared with GL‐3 plants, *MdDSK2a‐like* RNAi plants had lower MDA content, whereas *MdDSK2a‐like* OE plants had higher MDA (Figure 7c). After freezing treatment (−10 °C for 20 min), *MdDSK2a‐like* OE plants had lower survival rates than GL‐3 plants. In contrast, the survival rates of *MdDSK2a‐like* RNAi plants were higher than those of GL‐3 plants after treatment at −10 °C for 25 min (Figure 7d,e). The survival rates of GL‐3 and *MdDSK2a‐like* transgenic plants after cold acclimation had similar pattern with non‐cold acclimation conditions (Figure 7d,e). The above results suggest that MdDSK2a‐like plays a negative role under cold stress.

We also obtained *MdDSK2a‐like* RNAi calli to further verify the function of MdDSK2a‐like in cold tolerance. Under control conditions, the relative growth of *MdDSK2a‐like* RNAi calli was comparable with that of the wild type (WT) (Figure S24, Supporting Information). Under cold stress, the relative growth of *MdDSK2a‐like* RNAi calli was significantly higher than that of the WT (Figure S24, Supporting Information). These results further illustrate that MdDSK2a‐like negatively regulates cold tolerance in apple.

### 2.7 MdDSK2a‐Like Acts Upstream of MdMTA Under Cold Stress

**Figure 8:**
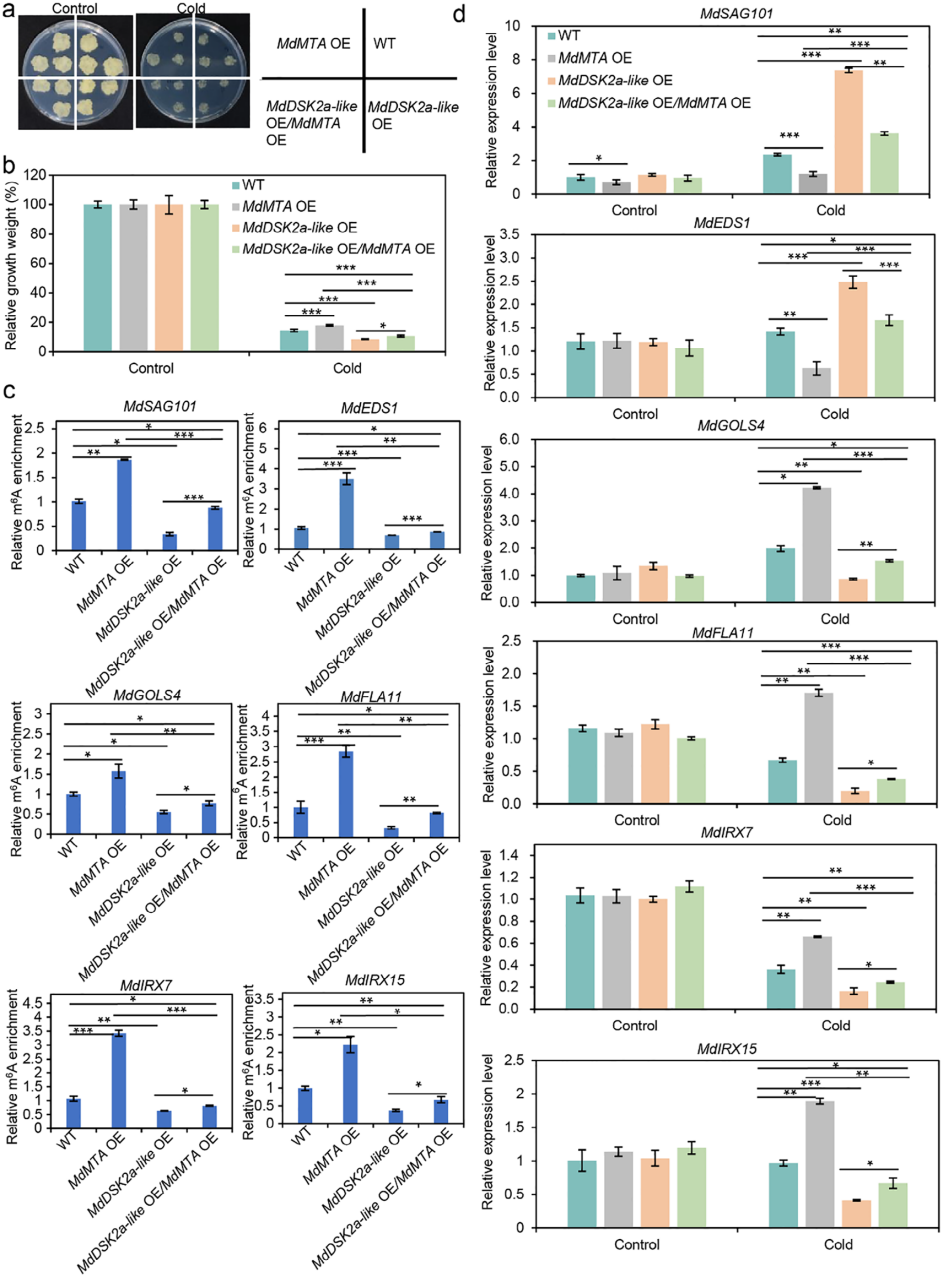
Genetic interaction of MdMTA and MdDSK2a‐like. a) Phenotype of the wild type, *MdDSK2a‐like* OE, *MdMTA* OE, and *MdDSK2a‐like* OE/*MdMTA* OE transgenic calli under control or cold conditions. Control conditions, transgenic calli were cultured at 22 °C for 2 weeks. Cold conditions, transgenic calli were cultured at 22 °C for 1 week and then transferred to 4 °C for additional 1 week. b) Relative growth weight of wild type, *MdDSK2a‐like* OE, *MdMTA* OE, and *MdDSK2a‐like* OE/*MdMTA* OE transgenic calli under control or cold conditions. c) m^6^A levels of mRNAs involved in ROS detoxification and deposition of cellulose and hemicellulose under cold conditions. d) Expression of the genes shown in (c) in wild type, *MdDSK2a‐like* OE, *MdMTA* OE, and *MdDSK2a‐like* OE/*MdMTA* OE transgenic calli under control and cold conditions. Two‐week‐old calli were treated at 0 °C for 10 h and RNA was extracted for m^6^A‐IP‐qPCR and qRT‐PCR. The asterisks indicate significant differences between the wild type and transgenic calli based on Tukey's test (*P < 0.05; **P < 0.01; ***P < 0.001). The error bars indicate standard deviations (*n* = 9 in (b), 3 in (c) and (d)). WT, wild type.

To further investigate the relationship between MdMTA and MdDSK2a‐like under cold stress, we obtained transgenic calli, including *MdMTA* OE, *MdDSK2a‐like* OE, and *MdDSK2a‐like* OE*/MdMTA* OE calli, and analyzed their phenotypes upon cold stress conditions (Figure S15, Supporting Information). Under control conditions, the relative growth was comparable between the transgenic calli and WT (**Figure** **8**a,b). However, *MdMTA* OE calli showed a higher growth rate, and *MdDSK2a‐like* OE calli exhibited a lower growth rate than the WT under cold stress (Figure 8a,b). Moreover, *MdDSK2a‐like* OE*/MdMTA* OE calli had higher relative fresh weight compared to the *MdDSK2a‐like* OE calli but was lower than *MdMTA* OE calli under cold stress, suggesting that MdMTA acts downstream of MdDSK2a‐like in cold stress response (Figure 8a,b).

Next, we examined the m^6^A levels and expression levels of the six MdMTA target genes. Under control conditions, the m^6^A levels of *MdSAG101, MdEDS1, MdGOLS4, MdFLA11, MdIRX7*, and *MdIRX15* showed no significant difference between WT and transgenic calli, indicating that neither MdMTA nor MdDSK2a‐like regulates the m^6^A levels of these genes under control conditions (Figure S25, Supporting Information). Under cold conditions, the m^6^A levels of *MdSAG101, MdEDS1, MdGOLS4, MdFLA11, MdIRX7*, and *MdIRX15* were increased in *MdMTA* OE calli but decreased in *MdDSK2a‐like* OE calli than the WT. Under cold stress, overexpression of *MdDSK2a‐like* in *MdMTA* OE calli decreased the m^6^A levels of these six genes, as compared to those in *MdMTA* OE calli, indicating that MdDSK2a‐like acts upstream of MdMTA in m^6^A regulation (Figure 8c). The expression levels detection further reveal that MdDSK2a‐like acts upstream of MdMTA in response to cold stress (Figure 8d).

On the other hand, we also obtained transgenic apple calli interfering *MdDSK2a‐like* only (*MdDSK2a‐like* RNAi), interfering *MdMTA* only (*MdMTA* anti), or reducing *MdMTA* in *MdDSK2a‐like* RNAi transgenic calli (*MdMTA* anti/*MdDSK2a‐like* RNAi). Under control conditions, the relative growth was comparable between the transgenic calli and WT (Figure S26, Supporting Information). Under cold stress, *MdDSK2a‐like* RNAi calli showed higher growth rate, but *MdMTA* anti calli exhibited lower growth rate than the WT (Figure S26, Supporting Information). The relative growth rate of *MdMTA* anti*/MdDSK2a‐like* RNAi calli was lower than that of WT, which was consistent with that of *MdMTA* anti calli under cold stress, further indicating that MdMTA acts downstream of MdDSK2a‐like in cold stress response (Figure S26, Supporting Information).

### 2.8 The Phylogenetic Analysis of DSK2 Homologs and Identification of MdDSK2a‐Like Functional Domains in Apple Cold Response

**Figure 9:**
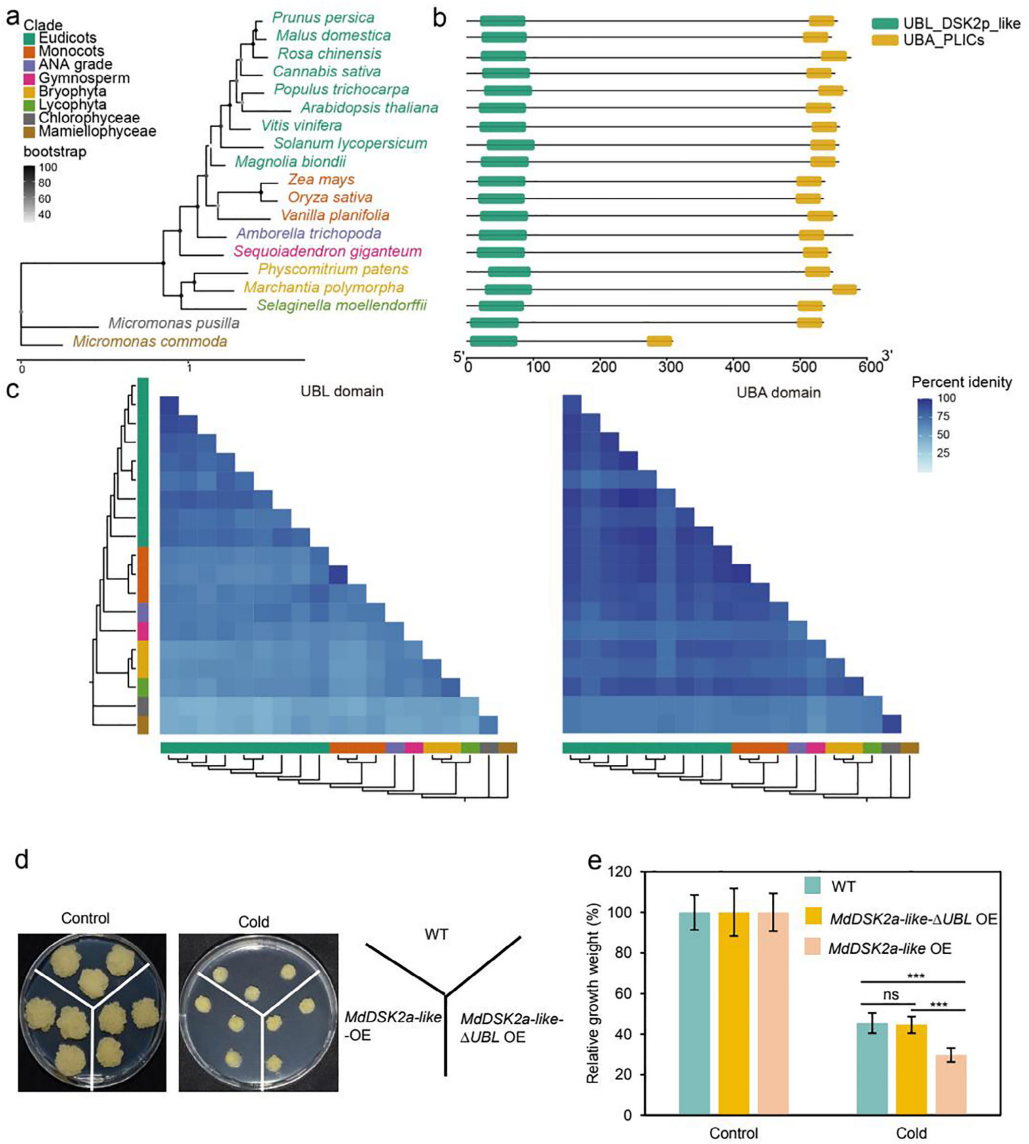
Evolution of the DSK2 family in land plants and green algae. a) Phylogenetic tree of DSK2 family proteins. b) General domain structure in DSK2 family proteins, including UBL and UBA domains. c) Conservation of DSK2 family domains. The heatmap shows percent identity of alignments within UBL and UBA domains, with darker blues indicating higher conservation. The order and color of clade are the same as in (a). d) Phenotype of the wild type, *MdDSK2a‐like* OE, and *MdDSK2a‐like‐∆UBL* OE transgenic calli under control and cold conditions. Control conditions, transgenic calli were cultured at 22 °C for 2 weeks. Cold conditions, transgenic calli were cultured at 22 °C for 1 week and then transferred to 4 °C for additional 1 week. e) Relative growth weight of wild type, *MdDSK2a‐like‐∆UBL* OE, and *MdDSK2a‐like* OE transgenic calli under control and cold conditions. The asterisks indicate significant differences between the WT and transgenic calli based on Tukey's test (***P < 0.001), whereas “ns” indicates not significant (*P* > 0.05). The error bars indicate standard deviations (*n* = 9 in (e)). WT, wild type.

To study the evolution of DSK2, MdDSK2a‐like protein sequence was used to identify the orthologous genes in different plant species. Based on the published plant genome database, a total of 19 species were selected on which to perform comparative analysis, including nine eudicots, three monocots, one basal angiosperm, one gymnosperm, two mosses, one fern, and two algae. The protein sequences from these species were obtained from the Plaza database and used for construction of the maximum likelihood tree.^[^ ^71^ ^]^ As expected, the phylogeny of DSK2 homologs mostly follows species‐level phylogenies (**Figure** **9**a). To shed light on the evolution of DSK2 function, we computed features of the aligned protein sequences. These features are uniform across the DSK2 family, indicating that UBL and UBA domains are essential for DSK2 function (Figure 9b). To study the sequence conservation of UBL and UBA domains, the aligned protein sequences of DSK2 were examined. The result shows that the UBL and UBA domains are more conserved in eudicots than in the other land plants and green algae (Figure 9c). Different from the UBL domains, the UBA domains are well‐conserved in land plants, especially in eudicots (Figure 9c). In comparison, the UBA domains were more conserved than UBL domains in land plants. These results suggest that DSK2 proteins are conserved in land plants, especially its UBA domain, which may be due to the biological necessity.

To study the biological importance of UBA and UBL domains, we generated transgenic calli expressing *35S:MdDSK2a‐like* without the UBA domain (*MdDSK2a‐like‐ΔUBA* OE) and *35S:MdDSK2a‐like* without the UBL domain (*MdDSK2a‐like‐ΔUBL* OE). In yeast, lack of UBA domain leads to the degradation of DSK2 protein.^[^ ^72^ ^]^ To determine the influence of UBA and UBL domains on the protein stability of MdDSK2a‐like protein, we detected protein levels in *MdDSK2a‐like‐ΔUBL* OE, *MdDSK2a‐like‐ΔUBA* OE, and *MdDSK2a‐like* OE calli. Western blot results indicated that both the absence of the UBA and UBL domains led to the degradation of MdDSK2a‐like, with lack of UBA domain resulting in complete degradation of MdDSK2a‐like protein (Figure S27a, Supporting Information). This suggests that both the UBA and UBL domains are required for the stability of MdDSK2a‐like, and thus for its full function in cold tolerance, regulation of m^6^A levels, and modulation of target gene expression levels. As lack of the UBA domain resulted in complete instability of MdDSK2a‐like protein, phenotypes of *MdDSK2a‐like‐ΔUBA* OE should be similar to the wild type. Therefore, we did not examine the phenotypes of *MdDSK2a‐like‐ΔUBA* OE. However, we analyzed the phenotypes of *MdDSK2a‐like‐ΔUBL* OE in response to cold stress (Figure S27b, Supporting Information). Under cold conditions, *MdDSK2a‐like‐ΔUBL* OE calli had a comparable relative growth rate to the wild type but had a higher relative growth rate than the *MdDSK2a‐like* OE calli (Figure 9d,e), indicating that the UBL domain of MdDSK2a‐like is also required for the negative role of MdDSK2a‐like in cold tolerance.

## 3 Discussion

**Figure 10:**
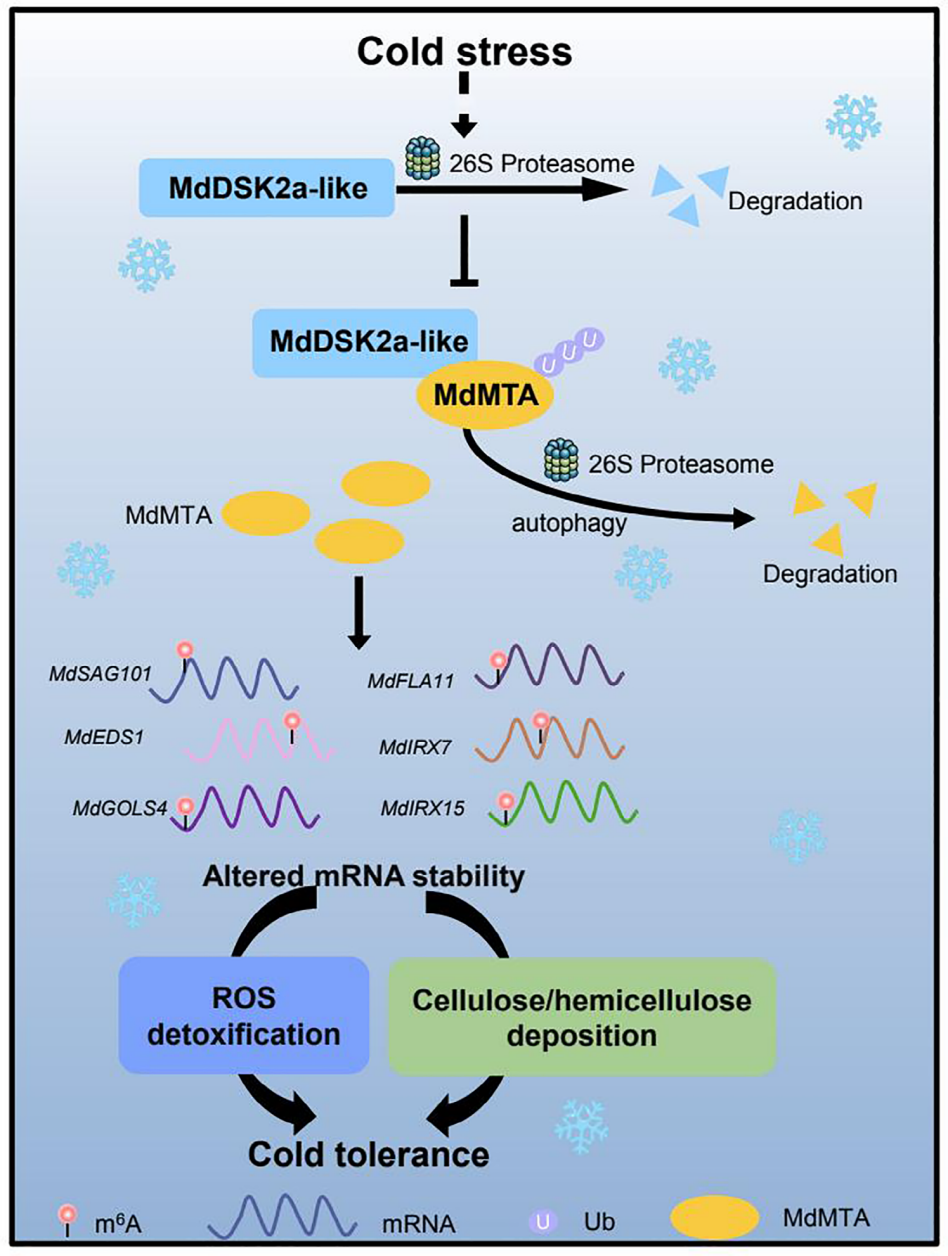
A proposed model of the MdDSK2a‐like‐MdMTA module in response to cold stress in apple trees. Cold stress promotes the degradation of MdDSK2a‐like protein through the 26S proteasome pathway, thereby alleviating the degradation of MdMTA protein induced by the 26S proteasome and autophagy pathways mediated by MdDSK2a‐like protein. The accumulated MdMTA increases the m^6^A levels of cold‐responsive genes involved in ROS detoxification and the deposition of cellulose and hemicellulose, resulting in altered mRNA stability and expression levels of these genes, which contributes to increased ROS detoxification, deposition of cellulose and hemicellulose, as well as apple cold tolerance.

As the most pervasive and highly conserved RNA modification, m^6^A not only regulates plant development and growth, but also regulates plant response to various stresses.^[^ ^20^, ^24^, ^26^, ^27^, ^30^, ^35^, ^36^, ^37^, ^38^ ^]^ Recently, studies on the regulation of m^6^A machinery have been increasing in animal and human cells.^[^ ^39^, ^42^ ^]^ However, studies regarding the regulation of m^6^A machinery including m^6^A methylases in plants are rarely reported. In this study, we for the first time identified that MdDSK2a‐like, a ubiquitin receptor protein, could promote MdMTA degradation through the autophagy and 26S proteasome pathways (Figure 5a–e). Both MdDSK2a‐like and MdMTA could interact with MdATG8i‐1 in vivo, thereby leading to the degradation of MdMTA through the autophagy pathway (Figure 5i and Figure S18b–d, Supporting Information). In addition, MdDSK2a‐like could promote the ubiquitination of MdMTA *in planta*, resulting in the degradation of MdMTA through the 26S ubiquitin‐dependent proteasome pathway (Figure 5e,f), which might be mediated by MdAIRP2 (Figure 5g,h). Moreover, we also explored the molecular mechanism of MdDSK2a‐like‐MdMTA regulatory module in response to apple cold hardiness. Under cold stress, MdDSK2a‐like, a ubiquitin receptor protein, was degraded by the 26S proteasome pathway (Figure 5j), thereby alleviating the degradation of MdMTA mediated by MdDSK2a‐like through 26S proteasome and autophagy pathways. The accumulated MdMTA then increased the m^6^A levels of cold‐responsive genes, including *MdSAG101, MdEDS1, MdGOLS4, MdFLA11, MdIRX7*, and *MdIRX15*, which in turn altered their expression and finally increased ROS scavenging, deposition of cellulose and hemicellulose, as well as apple cold tolerance (**Figure** **10**).

Our results first revealed the ubiquitination modification of m^6^A methyltransferase in plants. In human cells, the ubiquitin ligase STUB1 is shown to interact with and degrade METTL3.^[^ ^45^ ^]^ To explore which E3 ligase is responsible for attaching ubiquitin to MdMTA in apple, we performed Y2H screening of MdMTA and identified an E3 ligase, MdAIRP2. However, MdAIRP2 did not interact with MdMTA in yeast (Figure S13, Supporting Information). We speculate that this lack of interaction is due to the limitations of the Y2H system. Therefore, we analyzed their interaction relationship using Split‐LUC and Co‐IP. Fortunately, MdAIRP2 interacts with MdMTA in vivo (Figure 5g,h). These results suggest that it is possible that the ubiquitination of MdMTA in vivo is mediated by MdAIRP2 protein. However, other E3 ligases might be involved, and this needs further investigation.

AtDSK2a interacts with AtATG8 in vitro and in vivo to target AtBES1 for selective autophagy, and this degradation is dependent on AtBIN2‐mediated phosphorylation of AtDSK2a.^[^ ^55^ ^]^ In order to explore how MdDSK2a‐like degrades MdMTA through the autophagy pathway, we also examined the interaction between MdDSK2a‐like and MdATG8 in apple. Despite the high homology of the DSK2 and ATG8 proteins in apple and *Arabidopsis thaliana*, MdDSK2a‐like only interacts with MdATG8 in vivo but not in vitro (Figures S17 and S18, Supporting Information). Notably, although AtDSK2a and AtDSK2b are highly homologous, AtDSK2b and AtATG8a do not interact in yeast.^[^ ^73^ ^]^ The above results indicate that the relationship between DSK2 and ATG8 is complicated. In addition, the amino acids of apple MdDSK2a‐like where AtDSK2a undergoes phosphorylation modification in *Arabidopsis thaliana* are not identical.^[^ ^55^ ^]^ Therefore, we did not proceed to explore the interactions between phosphorylated MdDSK2a‐like and MdATG8. These results suggest that the mechanism of MdDSK2a‐like‐mediated autophagy degradation in apple may be different from that in *Arabidopsis thaliana*, which requires further in‐depth study.

Under control conditions, MdDSK2a‐like degrades MdMTA, a positive regulator of apple plant height and root development,^[^ ^37^ ^]^ raising a question of the biological function of this degradation. We speculate that there may be several possibilities for this. First, the components of the m^6^A methyltransferase complex can affect each other at the protein level. In *Arabidopsis thaliana*, MTA and MTB affect each other's protein levels, but neither regulates FIP37 protein level.^[^ ^28^ ^]^ In mammalian cells, METTL3 and METTL14 not only affect each other's protein levels, but METTL3 inhibits WTAP (Wilms tumor 1‐associated protein) protein level.^[^ ^74^ ^]^ Therefore, an inter‐regulatory interaction between m^6^A methyltransferases in apple might exist. MdDSK2a‐like degradation of MdMTA under control conditions may alleviate the regulatory effect of MdMTA on other m^6^A methylases to maintain the homeostasis of m^6^A levels. Second, two methyltransferases, MTA and MTB, and several accessory subunits, such as VIR, FIP37, and HAKAI, form a complex responsible for m^6^A deposition in *Arabidopsis thaliana*.^[^ ^24^, ^26^, ^27^, ^75^ ^]^ As a long‐standing view, the individual subunits that form protein complexes are more stable.^[^ ^76^, ^77^ ^]^ Individual subunits that fail to assemble into protein complexes (called “orphan subunits”) are unstable and susceptible to degradation, such as due to the absence of their protein chaperone.^[^ ^78^, ^79^ ^]^ Hence, MdMTA protein degraded by MdDSK2a‐like under control conditions may be the portion which does not form a stable complex, but this needs to be further explored. Third, m^6^A is a double‐edged sword during tumor development,^[^ ^80^ ^]^ suggesting that the precise regulation of m^6^A levels may be essential for normal life activities. Consequently, MdDSK2a‐like degradation of MdMTA may also function to maintain the precise regulation of m^6^A levels in apple to avoid deleterious effects in plant growth and development. Although MdDSK2a‐like degraded MdMTA under control conditions, it did not cause changes in the m^6^A levels of the downstream target genes (Figure S21, Supporting Information). This may be due to the fact that MdMTA did not regulate the m^6^A levels of these genes under control conditions (Figure S9, Supporting Information). However, it is worth noting that MdDSK2a‐like negatively regulates m^6^A levels under control conditions (Figure S20, Supporting Information), indicating that MdDSK2a‐like might regulate the m^6^A levels of genes involved in other pathways through MdMTA, such as growth and development.

Another significant finding of our study is the MdDSK2a‐like‐MdMTA regulatory module response to apple cold hardiness. In *Arabidopsis thaliana*, MTA positively regulates cold tolerance by regulating the translation efficiency of *ACYL‐COA:DIACYLGLYCEROL ACYLTRANSFERASE 1 (DGAT1)* rather than its transcript levels.^[^ ^48^ ^]^ In addition, MTA regulates the ribosome occupancy of cold‐responsive genes to attenuate the effects of cold stress on *Arabidopsis thaliana*.^[^ ^49^ ^]^ Although the relationship between MTA and cold stress has been reported, it has only studied the target genes of MTA. In this study, we not only explained the function of MdMTA in cold response from the target genes of MdMTA, but more importantly, we clarified how the ubiquitination modification of MdMTA responds to cold stress at the post‐translational modification level. We found that cold stress weakens the degradation of MdMTA mediated by MdDSK2a‐like (Figure 5e), and then accumulates more MdMTA for regulating the m^6^A levels of genes involved in ROS scavenging and the deposition of cellulose and hemicellulose to improve apple cold tolerance (Figures 2, 3, and 4). Further study showed that cold stress promotes the degradation of MdDSK2a‐like through the 26S proteasome pathway (Figure 5j). In addition, MdDSK2a‐like negatively regulates apple cold tolerance by regulating the m^6^A levels of MdMTA target genes (Figures 6 and 7). Considering together, our results expand the role of ubiquitination modification of MdMTA in cold response and clarify that MdDSK2a‐like regulates apple cold tolerance by regulating the m^6^A modification of cold responsive genes.

Under cold conditions, *MdDSK2a‐like‐ΔUBL* OE calli showed similar relative fresh weight to the wild type (Figure 9d,e). DSK2 proteins contain an N‐terminal ubiquitin‐like (UBL) domain that mediates their interaction with the proteasome and a C‐terminal ubiquitin‐associated (UBA) domain that can bind both K48 and K63 polyubiquitin chains.^[^ ^81^ ^]^ Therefore, the lack of the UBL domain in MdDSK2a‐like will inhibit the delivery of the ubiquitinated MdMTA to the proteasome pathway for degradation, leading to the observed phenotype of *MdDSK2a‐like‐ΔUBL* OE calli under cold stress.

In summary, our results reveal the degradation regulation of MdMTA by MdDSK2a‐like and explore the molecular mechanisms of MdDSK2a‐like‐MdMTA in response to cold stress, providing the insights into the post‐translational modifications of m^6^A methyltransferase in apple cold response.

## 4 Experimental Section

### Plant Materials and Growing Conditions

The *MdMTA* transgenic plants were obtained previously.^[^ ^37^ ^]^ GL‐3, a progeny of ‘Royal Gala’, was cultured for 1 month on Murashige and Skoog (MS) media supplemented with phytohormones (4.43 g L^−1^ MS salts, 30 g L^−1^ sucrose, 0.3 mg L^−1^ 6‐BA, 0.2 mg L^−1^ IAA, and 7.5 g L^−1^ agar, pH 5.8), and then was used for genetic transformation of apple. Tissue‐cultured plants were rooted on MS media supplemented with phytohormones (4.43 g L^−1^ MS salts, 30 g L^−1^ sucrose, 0.5 mg L^−1^ IBA, 0.5 mg L^−1^ IAA, and 7.5 g L^−1^ agar, pH 5.8). Both tissue‐cultured and soil‐grown plants were grown in long‐day conditions (16 h light/8 h dark) at 22 °C.

‘Orin’ calli grown on MS media (4.43 g L^−1^ MS salts, 30 g L^−1^ sucrose, 0.4 mg L^−1^ 6‐BA, 1.5 mg L^−1^ 2,4‐D, and 7.5 g L^−1^ agar, pH 5.8) under dark conditions were used for genetic transformation work.

For MeRIP‐seq and RNA‐seq, the same batch of 2‐month‐old *MdMTA* RNAi and GL‐3 plants treated at 0 °C for 10 h or grown at 22 °C was used as the plant materials for the cold and control group, respectively. For the control group, MeRIP‐seq and RNA‐seq data of GL‐3 plants have been reported in the previous study.^[^ ^37^ ^]^

### Genetic Transformation Work

To obtain the *MdDSK2a‐like* transgenic plants, 239 bp of the *MdDSK2a‐like* sequence was cloned into the interference vector pK7WIWG2D and the CDS (coding sequence) of *MdDSK2a‐like* was cloned into the pKGMYC vector to generate the overexpression plasmid. The successfully constructed interference and overexpression vectors were transferred into *Agrobacterium* EHA105, and then transformed into GL‐3 plants according to the previous method.^[^ ^82^ ^]^ The primers used are listed in Table S3 (Supporting Information).

To generate *MdDSK2a‐like* or *MdMTA* overexpressing apple calli, the CDS of *MdDSK2a‐like* or *MdMTA* was cloned into the pCAMBIA1300 (hygromycin resistance) or pGWB411 (kanamycin resistance) vector, respectively. The constructed *35S:MdDSK2a‐like* or *35S:MdMTA* was transformed into *Agrobacterium* EHA105, and then introduced into the ‘Orin’ calli, which were used as the wild type.^[^ ^83^ ^]^ *35S:MdDSK2a‐like* (hygromycin resistance) plasmid was transferred into *MdMTA* overexpressing calli to obtain transgenic calli that overexpressed both *MdDSK2a‐like* and *MdMTA*. The primers used are listed in Table S3 (Supporting Information).

To knock down *MdMTA*, a 287 bp antisense fragment of *MdMTA* was cloned into pCAMBIA1300 (hygromycin resistance). The plasmid pK7WIWG2D was used to interfere *MdDSK2a‐like*. Besides, *MdMTA‐*pCAMBIA1300 plasmid was introduced into *MdDSK2a‐like* RNAi calli to generate transgenic calli repressing both *MdDSK2a‐like* and *MdMTA*. The primers used are listed in Table S3 (Supporting Information).

### Cold Stress Treatment

Two‐month‐old plants were used to evaluate freezing tolerance. For cold acclimation, plants were grown in long‐day conditions (16 h light/8 h dark) at 4 °C for 1 week. Fully expanded leaves were selected and perforated into leaf discs of 5 mm diameter. Four leaf discs were placed in a test tube containing 1 mL of deionized water and the test tube was quickly placed on ice. The tubes were placed in the low‐temperature aqua bath cycle instrument (Thermo Fisher PC200‐A40). The freezing regime and the measurement of electrolyte leakage were performed as previously reported.^[^ ^84^ ^]^ Each sample had six biological replicates.

Whole‐plant freezing assays were treated at −10 °C for 20, 25, or 30 min, then were recovered under long‐day conditions (16 h light/8 h dark) at 22 °C for 1 month.

For the cold tolerance assay of apple calli, 1‐week‐old calli were treated at 4 °C for 1 week. The calli grown at 24 °C were used as control.

### MeRIP‐seq and Data Analysis

50 µg of total RNA was extracted from apple leaves by TRIzol Reagent (Invitrogen, cat. NO 15596026), and was used for polyadenylated RNA enrichment by VAHTS mRNA Capture Beads (VAHTS, cat. NO. N401‐01/02). Subsequently, 20 mm ZnCl_2_ was used to fragment polyadenylated RNA into ≈100–200 nucleotide‐long oligonucleotides. 10% of the RNA fragments were saved as non‐immunoprecipitated RNA (Input) and the rest of the RNA fragments were used for m^6^A immunoprecipitation (IP). A specific anti‐m^6^A antibody (Synaptic Systems, 202203) was used for m^6^A immunoprecipitation. The stranded RNA sequencing library was constructed using the KC‐Digital Stranded mRNA Library Prep Kit for Illumina (Catalog No. DR08502, Wuhan Seqhealth Co., Ltd., China) following the manufacturer's instructions. To eliminate duplicate bias introduced in library preparation and sequencing, we introduced Unique Identifier (UMI) sequences.

The quality of raw sequencing reads was first assessed by the FastQC v0.11.9 and MultiQC v1.10.1.^[^ ^85^ ^]^ Trimmomatic v0.39 was used to remove adaptor sequences and low‐quality bases (lower than 25).^[^ ^86^ ^]^ The clean reads were then mapped to the latest apple reference genome (https://iris.angers.inra.fr/gddh13/downloads/GDDH13_1‐1_formatted.fasta.bz2) by HISAT2 v2.1.0 using default parameters.^[^ ^87^ ^]^ BAM conversion, sorting, and indexing were performed using SAMtools v1.9.^[^ ^88^ ^]^ The ‐F “mapping_quality ≥ 20” parameter in Sambamba v1.0 was used to filter out low‐quality alignments, thereby ensuring the reliability of the data.^[^ ^89^ ^]^ Peaks of m^6^A modification were identified using exomePeak v2.16.0 with thresholds of *P* value < 0.05 and log_2_(fold enrichment) > 1.^[^ ^37^, ^90^ ^]^ Venn tools in Intervene v0.5.8 which based on bedtools were used for bed format files intersection and visualization.^[^ ^91^ ^]^ The significant peaks that were consistently called in all independent biological replicates and had a 50% intersection were considered as a common peak. The m⁶A motifs were identified using the findMotifsGenome.pl tool in HOMER v4.10.0.^[^ ^92^ ^]^ The parameters for running findMotifsGenome.pl were set as follows: ‐mcheck all.rna.motifs, ‐mknown known.rna.motifs, ‐rna, ‐mis 0, ‐len 4,6,8,10,12, and ‐known. Differentially methylated peaks were determined using exomePeak v2.16.0 with a threshold of *P* value < 0.05 and |DiffModLog_2_FC| > 0.5. The CMRAnnotation tool in PEA v1.1, bedtools v2.30.0, and deepEA were used to annotate the identified peaks with the apple genome annotation file (https://iris.angers.inra.fr/gddh13/downloads/gene_models_20170612.gff3.bz2).^[^ ^93^, ^94^, ^95^ ^]^ Pearson correlation analysis was performed using multiBamSummary and plotCorrelation in deepTools v3.1.3.^[^ ^96^ ^]^ BAM format files were transferred into bw format files using bamCoverage in deepTools, and visualization of m^6^A peaks was carried out using IGV v2.10.2.^[^ ^96^, ^97^ ^]^

### Dot Blot Assay

Procedure was referenced by previous work.^[^ ^98^ ^]^ In order to remove the secondary structure of RNA, the purified RNA was heated at 94 °C for 5 min, and then placed on the Hybond‐N^+^ membrane for UV cross‐linking. The cross‐linked membrane was washed with TBST buffer (TBS with Tween‐20) for 5 min and then blocked with 5% nonfat milk at room temperature for 1 h. m^6^A antibody (1:1000; Synaptic Systems) was then incubated at 4 °C overnight. After the addition of horseradish peroxidase‐conjugated anti‐rabbit IgG secondary antibody (ABclonal) and incubation, the signals were detected by ECL Western blotting assay kit (Bio‐Rad).

### Quantitative Analysis of m^6^A Level by LC‐MS/MS

One µg of RNA was digested with S1 nuclease, alkaline phosphatase, and phosphodiesterase I at 37 °C until the RNA was completely digested into nucleoside. Then, the mixture was extracted with chloroform and the resulting water layer was analyzed using LC‐ESI‐MS/MS. The sample extracts were analyzed using a UPLC‐ESI‐MS/MS system (UPLC, ExionLC AD; MS, Applied Biosystems 6500 Triple Quadrupole) equipped with an ACQUITY UPLC HSS T3 column (Waters) and then detected by a triple quadrupole‐linear ion trap mass spectrometer (QTRAP) in positive ion mode with scheduled multiple reaction monitoring. Data acquisition was performed using Analyst 1.6.3 software (Sciex). Multiquant 3.0.3 software (Sciex) was used to quantify all metabolites.

### UMI RNA‐seq and Data Analysis

Total RNA was extracted from leaves using the TRIzol reagent, following the manufacturer's protocol (Invitrogen, Carlsbad, CA, USA). Each group contains three independent biological replicates. RNA quality was determined by examining A260/A280 with Nanodrop OneC‐ spectrophotometer (Thermo Fisher Scientific lnc, USA). RNA‐seq was conducted by Seqhealth Technology Co., Ltd (Wuhan, China). Two µg of total RNA were used for stranded RNA‐sequencing library preparation using KC Stranded mRNA Library Prep Kit for Illumina (Catalog NO. DR084022, Wuhan Seqhealth Co., Ltd. China) following the manufacturer's instructions. PCR products corresponding to 200–500 bp were enriched, quantified, and finally sequenced on Illumina Hiseq platform with the PE150 model.

The quality of raw sequencing reads was assessed using FastQC v0.11.9 and MultiQC v1.10.1, followed by trimming of adapter sequences and bases with a Phred score below 25 using fastp v0.23.2.^[^ ^85^ ^]^ Clean reads were aligned to GDDH13 v1.1 reference genome using HISAT2 v2.1.0 with default parameters.^[^ ^87^ ^]^ Reads counting within genes was calculated by HTSeq v0.12.4 with the gene annotation file.^[^ ^99^ ^]^ Length of genes was calculated by GenomicFeatures v1.42.3, and fragments per kilobase of transcript per million fragments mapped (FPKM) values were calculated by TBtools.^[^ ^100^ ^]^ DEGs were identified using DESeq2 v1.30.1 with a threshold of an adjusted *P* value < 0.05 and |log_2_(fold change)| > 0.5.^[^ ^101^ ^]^ GO enrichment analyses were performed using agriGO v2.0 and clusterProfiler v3.18.1.^[^ ^102^, ^103^ ^]^

### m^6^A‐IP‐qPCR

m^6^A‐IP‐qPCR assay was performed as previously reported.^[^ ^104^ ^]^ m^6^A‐IP assay was carried out in the same way as MeRIP‐seq. Briefly, 300 µg of total RNA was extracted from apple leaves by the cetyltrimethylammonium bromide (CTAB) method. Then, mRNAs were isolated using VAHTS mRNA Capture Beads (VAHTS, cat. NO. N401‐01/02) and fragmented into ≈200–300 nucleotide‐long oligonucleotides by 20 mm ZnCl_2_. 10% of mRNAs were used as the input and the rest of mRNAs were incubated with m^6^A antibody and 20 µL of Protein‐A/G Sepharose beads (Sigma, USA) in 500 µL of IP buffer for 4 h at 4 °C. Beads were washed with IP buffer three times and the mRNAs were extracted using TRIzol Reagent, followed by ethanol precipitation. Random hexamers (Vazyme, R223‐01) were used to reverse transcribe input and immunoprecipitated RNA. Relative enrichment of each transcript was determined by quantitative real‐time PCR (qRT‐PCR) and normalized to the input level. Primers used are listed in Table S3 (Supporting Information).

### RNA Extraction and qRT‐PCR Assay

RNA extraction was performed by the CTAB method, and DNA removal was performed using the RNase‐free DNase I (Thermo Scientific, USA). The first‐strand cDNA synthesis was used HiScript II 1st Strand cDNA Synthesis Kit (+gDNA wiper) (Vazyme, R212‐01). qRT‐PCR was performed using Hieff Unicon Universal TaqMan multiplex qPCR master mix (YEASEN, 11211ES03). *MdMDH* was used as a reference gene. Primers used are listed in Table S3 (Supporting Information).

### mRNA Stability Assay

Two‐month‐old plants were treated with 10 µm of actinomycin D and dimethyl sulfoxide (DMSO), respectively. After treatment for 30 min, samples were collected as 0 h. Meanwhile, the control group was treated at room temperature, and the cold treatment group was treated at 0 °C. After 2 and 4 h, samples were collected for gene expression levels analysis. RNAs were extracted by CTAB methods and the mRNA levels of genes were examined by qRT‐PCR as described above. Primers used are listed in Table S3 (Supporting Information).

### Y2H Assay

The CDS region of *MdMTA*, *MdDSK2a‐like*, *MdDSK2a‐like* (1‐270 aa), *MdDSK2a‐like* (271‐425 aa), *MdDSK2a‐like* (426‐547 aa), *MdD6PKL2*, *MdPP2C*, *MdSTY13*, *MdAIRP2*, *MdRUB2*, *MdDESI1*, *MdATG8g‐1*, *MdATG8g‐2*, *MdATG8i‐1*, *MdATG8i‐2*, *MdATG8i‐3*, *MdATG8i‐4*, *MdATG8c‐1*, *MdATG8c‐2*, *MdATG8c‐3*, *MdATG8c‐4*, *MdATG8f‐1* or *MdATG8f‐2* was constructed into pGADT7 or pGBKT7 vector. The resulting constructs (MdMTA‐BD, MdDSK2a‐like‐AD, MdDSK2a‐like (1‐270 aa)‐AD, MdDSK2a‐like (271‐425 aa)‐AD, MdDSK2a‐like (426‐547 aa)‐AD, MdD6PKL2‐AD, MdPP2C‐AD, MdSTY13‐AD, MdAIRP2‐AD, MdRUB2‐AD, MdDESI1‐AD, MdATG8g‐1‐BD, MdATG8g‐2‐BD, MdATG8i‐1‐BD, MdATG8i‐2‐BD, MdATG8i‐3‐BD, MdATG8i‐4‐BD, MdATG8c‐1‐BD, MdATG8c‐2‐BD, MdATG8c‐3‐BD, MdATG8c‐4‐BD, MdATG8f‐1‐BD, MdATG8f‐2‐BD, and MdMTA‐AD) were transformed into Y2H competent cell, and grown on selection media (SD/‐Leu/‐Trp and SD/‐Leu/‐Trp/‐His/‐Ade). X‐α‐Gal was used to confirm the interaction. To detect the transformation efficiency, p53‐pGBKT7 and pLAM‐pGBKT7 served as positive and negative controls, respectively. Primers used are listed in Table S3 (Supporting Information).

### Protein Complex Prediction with AlphaFold2

Protein complex prediction was performed using AlphaFold2.^[^ ^105^ ^]^ Based on the Google Colaboratory, the prediction process was initiated via ColabFold v1.5.5 with the default settings.^[^ ^106^ ^]^ Template sequence searches against the UniRef30 with MMseqs2 were performed to construct the MSA for individual proteins.^[^ ^107^, ^108^ ^]^ Then AlphaFold2 was used to predict the 3D structures of the individual proteins and their interaction interface. The predicted structures were output in PDB format, accompanied by confidence scores, such as the predicted local distance difference test (pLDDT). The predicted protein complexes were visualized and analyzed using Pymol (Schrödinger).

### Split‐LUC Assay

The CDS of *MdMTA*, *MdDSK2a‐like*, or *MdATG8i‐1* was introduced into firefly luciferase complementation imaging vectors to obtain MdMTA‐cLUC, MdDSK2a‐like‐nLUC, MdATG8i‐1‐cLUC, and MdMTA‐nLUC, respectively. The resulting constructs were transformed into *Agrobacterium* strain C58C1. Different combinations were injected into *N. benthamiana* leaves and then cultured at 22 °C for 3 days. CCD camera (charge coupled device camera, Lumazone Pylon 2048B, USA) was used to capture the luciferase signal. Primers used are listed in Table S3 (Supporting Information).

### Co‐IP Assay

The CDS of *MdMTA*, *MdDSK2a‐like*, *MdAIRP2*, or *MdATG8i‐1* was introduced into pGWB411, pGWB418, PEG104, or PEG101 vector, respectively, to result in Flag‐MdMTA, MYC‐MdDSK2a‐like, GFP‐MdAIRP2, and GFP‐MdATG8i‐1. Tobacco transient expression experiments were performed according to the previous method.^[^ ^84^ ^]^ After tobacco was cultured at 22 °C for three days under a 16 h light/8 h dark period, proteins were extracted with extraction buffer (50 mm Tris‐HCl, pH 7.5, 150 mm NaCl, 2 mm ethylenediaminetetraacetic acid (EDTA), 10% glycerol, 1% NP‐40, 1 mm phenylmethylsulphonyl fluoride). The protein extracts were incubated with anti‐Flag or anti‐GFP (Abmart; 1:500 dilution) and Protein‐A/G Sepharose beads (Sigma, USA) for overnight at 4 °C. Beads were washed with protein extraction buffer three times and eluted by boiling in 1×SDS sample buffer. Western blots were analyzed by anti‐Flag, anti‐MYC, or anti‐GFP antibodies (1:5000 dilutions; Abmart, USA). Primers used are listed in Table S3 (Supporting Information).

### Protein Stability Analysis and Inhibitor Treatments

Two‐week‐old transgenic calli were rotated with MS liquid medium containing DMSO, 50 µm MG132, and 20 µm E64d, respectively, for 12 h at 24 and 0 °C. Then, proteins were extracted with extraction buffer (50 mm Tris‐HCl, pH 7.5, 150 mm NaCl, 2 mm EDTA, 10% glycerol, 1% NP‐40, 1 mm phenylmethylsulphonyl fluoride). Western blots were analyzed by anti‐Flag or anti‐Actin antibodies (1:5000 dilutions; Abmart, USA).

### Phylogenetic Analyses

From the plaza database, DSK2s across plants were identified using BLASTP search.^[^ ^71^, ^109^ ^]^ The sequences with a threshold of *E*‐value below 1×10^−10^ and length greater than 200 aa were retained. The curated sequences were aligned by MAFFT^[^ ^110^ ^]^ and trimmed gaps by trimAl with default parameters.^[^ ^111^ ^]^ After that, the maximum‐likelihood tree was created using IQ‐TREE 2 with “JTT+F+I+G4” model based on the trimmed multiple sequence alignment.^[^ ^112^ ^]^

Identification of conserved domains was performed using the Conserved Domain Database in NCBI.^[^ ^109^ ^]^ The pairwise percentage amino acid identity was computed in the trimmed alignments for the UBL (MdDSK2a‐like 22–90 aa) and the UBA domain (MdDSK2a‐like 377–413 aa). The phylogenetic tree and heatmap of pairwise percent amino acid identity were plotted as previous study.^[^ ^113^ ^]^

### Subcellular Localization Assay

The CDS of *MdDSK2a‐like* was cloned into the pGWB405 vector. pGWB405‐MdDSK2a‐like and pGWB455‐MdSUMO2A were transformed into the *Agrobacterium* strain C58C1, respectively, and mixed with *35S:p19* at a 1:1:1 ratio for tobacco injection. After tobacco was cultured at 22 °C for 3 days under a 16 h: 8 h, light‐dark period, Leica TCS SP8 SR, and LAX software were used to observe the fluorescence signal of the samples under 40x water immersion lens. Primers used are listed in Table S3 (Supporting Information).

### Measurement of the Content of H_2_O_2_, O_2_^−^, Cellulose, and Hemicellulose, Along with the Activities of POD, CAT, and MDA

Reagent kits from Conmin Biotechnology Company (Suzhou, China) were used to measure H_2_O_2_ (#H_2_O_2_‐1‐Y), O_2_^−^ (#SA‐2‐G), cellulose (#CLL‐1‐Y), and hemicellulose (#BXW‐1‐G) content. POD activity, CAT activity, and MDA content were detected with reference to Niu et al.^[^ ^114^ ^]^

### Tissue Staining and Microscopy

Two‐three mm plant leaf tissues were fixed in formaldehyde‐acetic acid‐alcohol solution. Tissues were embedded in paraffin, dried, and stained with toluidine blue. The BX53 fluorescence microscope (Olympus, Tokyo, Japan) was used for the histological observation. Cell wall thickness was measured by the software of ImageJ.

## Conflict of Interest

The authors declare no conflict of interest.

## Author Contributions

J.H. and C.B. are co‐first authors. N.H., J.H., and C.B. contributed equally to this work. Q.G. and N.H. designed the project. N.H., C.B., X.S., F.Z., C.L., T.F., X.Y., B.C., G.Q., M.M., and X.L. performed the experiments. J.H., Y.L., Z.L., C.M., B.T., J.F., and F.M. analyzed the data. N.H. and Q.G. wrote the manuscript.

## Supporting information



Supporting Information

Supplemental Table 1

## Data Availability

The RNA‐seq and m6A‐seq data have been deposited to the NCBI with the dataset identifier PRJNA1019695 and PRJNA1019696, respectively.
