# Supplementary material for: MdDSK2a‐Like‐MdMTA Module Functions in Apple Cold Response via Regulating ROS Detoxification and Cell Wall Deposition
Source: Adv Sci (Weinh). 2025 Apr 25;12(26):2504405. doi: 10.1002/advs.202504405 (PMC12245062; doi:10.1002/advs.202504405)
Supplement: Supplementary file 1 — Supporting Information [file ADVS-12-2504405-s001.docx]

**Supplemental Figures**


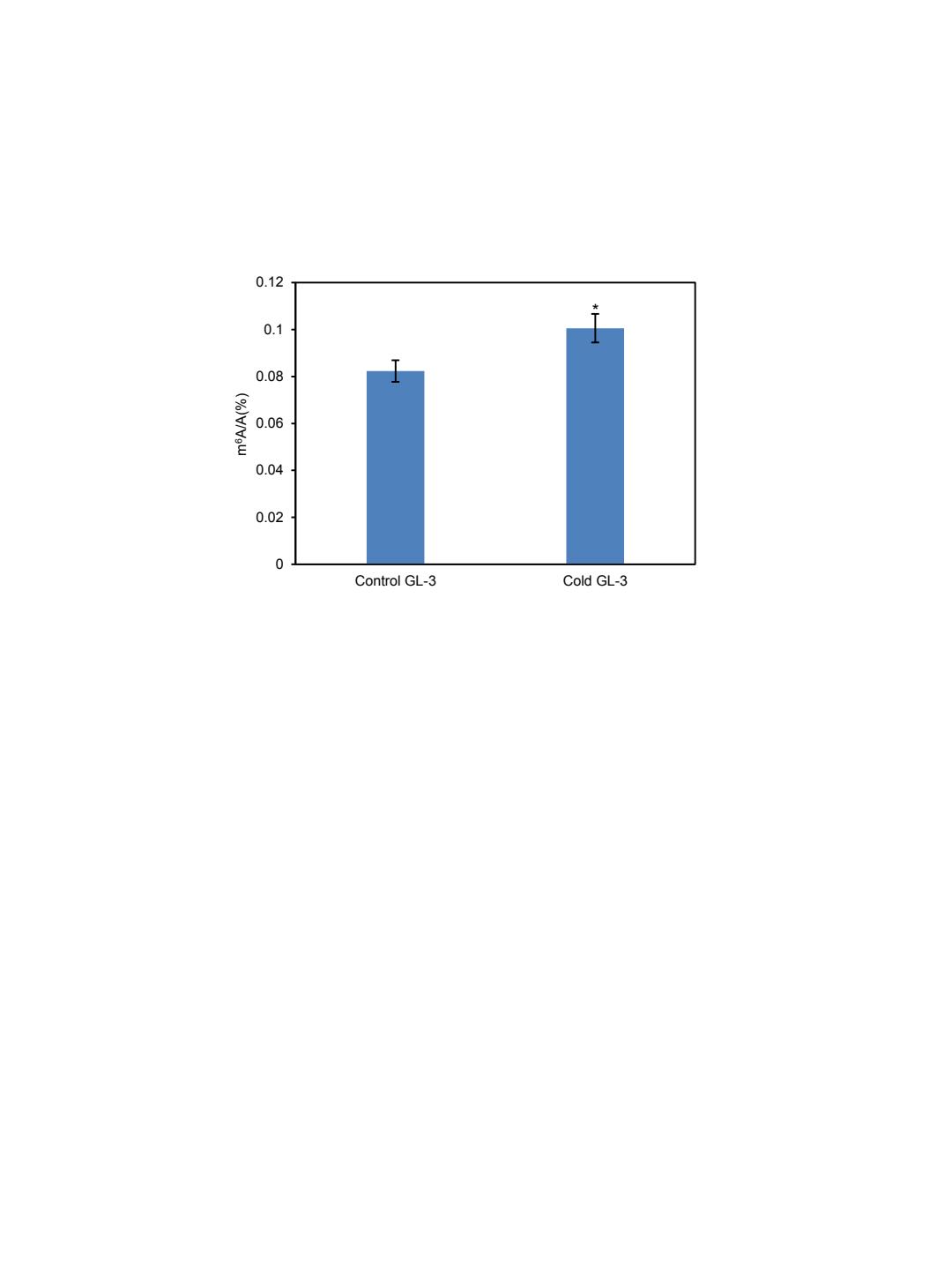


**Supplemental Figure 1.** LC-MS/MS detection of m^6^A levels in GL-3 plants under control and cold conditions. Control GL-3, two-month-old GL-3 plants grown at 22℃. Cold GL-3, two-month-old GL-3 plants were treated at 0℃ for 10 h. The asterisks indicate significant differences between the control GL-3 and cold GL-3 based on Tukey’s test (**P* < 0.05). The error bars indicate standard deviations (n = 3).


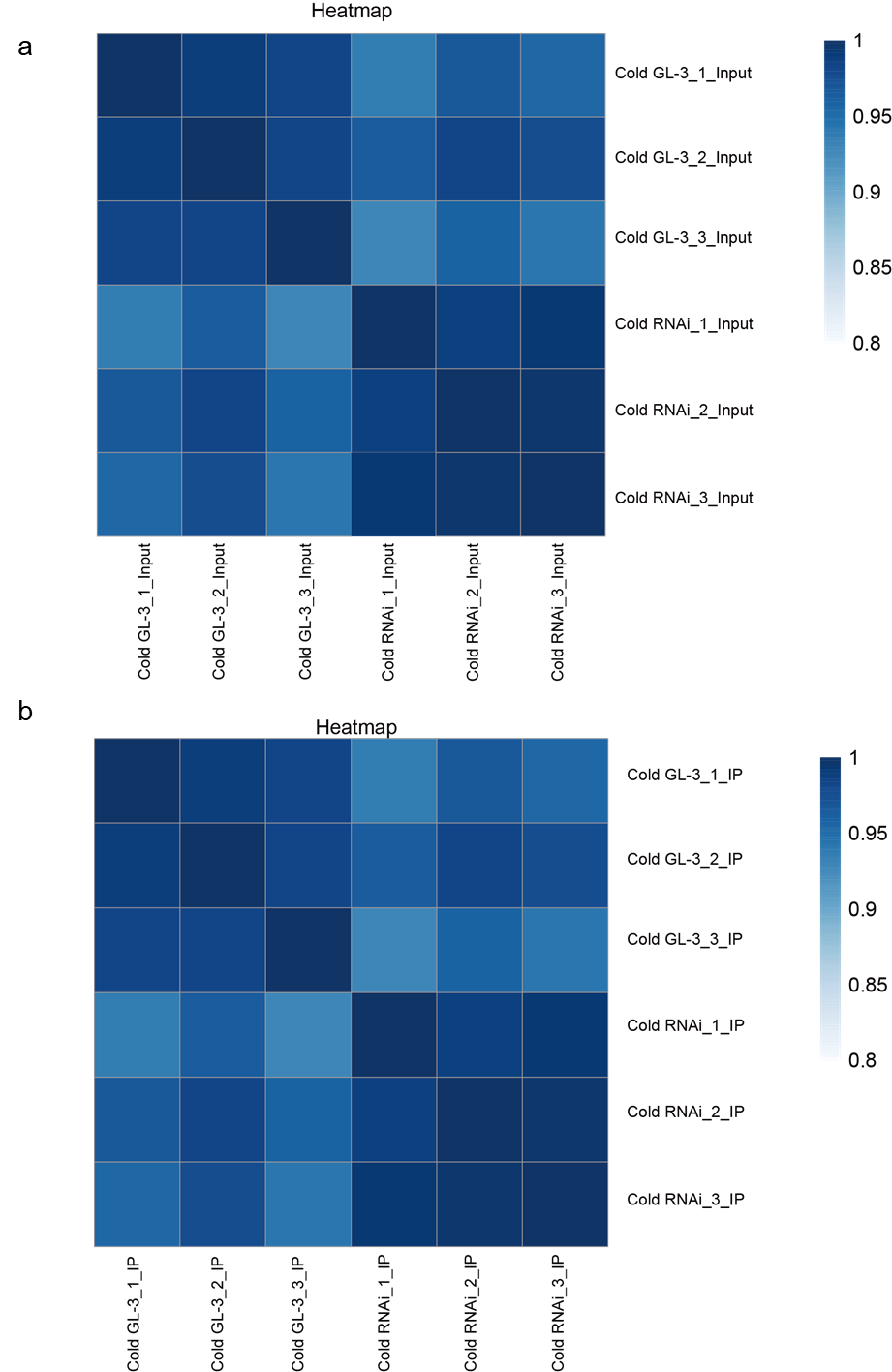


**Supplemental Figure 2.** Pearson correlation analysis of (a) input and (b) immunoprecipitation (IP) reads from the m^6^A peak regions identified by m^6^A-seq in GL-3 and *MdMTA* transgenic plants under cold conditions. Cold GL-3, two-month-old GL-3 plants were treated at 0℃ for 10 h. Cold RNAi, two-month-old *MdMTA* RNAi plants were treated at 0℃ for 10 h.


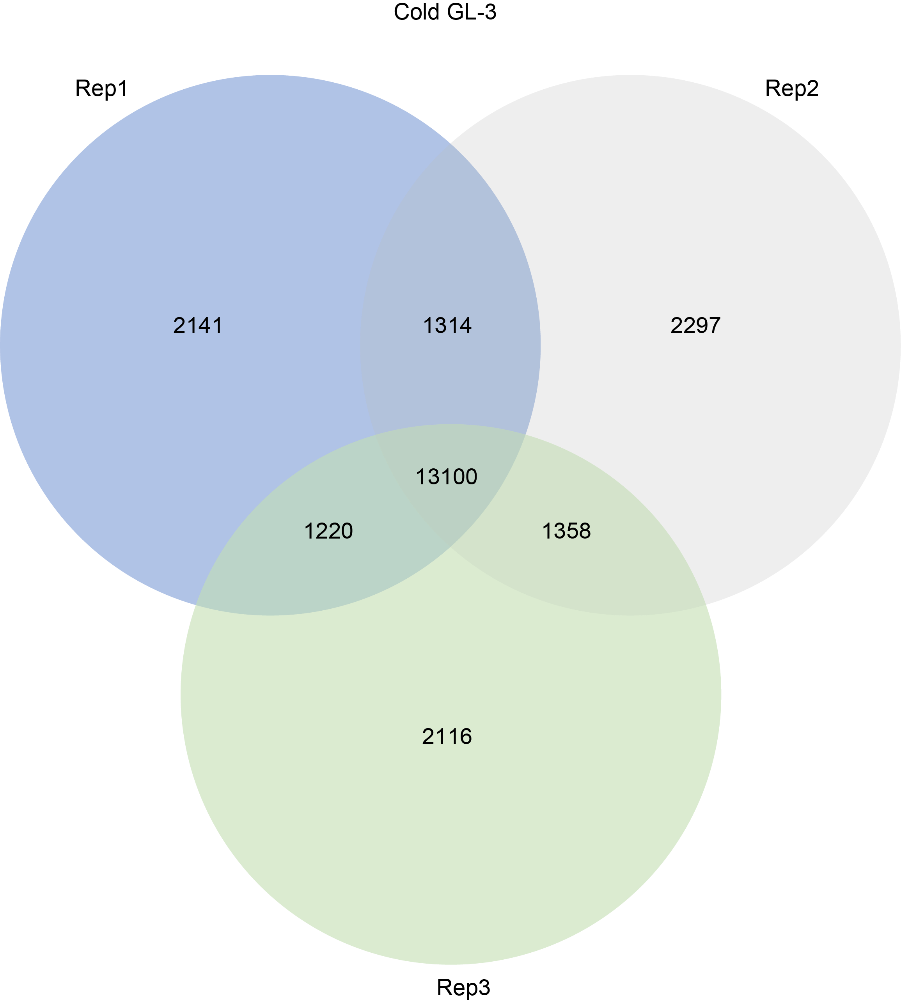


**Supplemental Figure 3.** Venn diagram showing the overlap of three independent m^6^A-seq experiments on GL-3 cold treatment groups. Rep, replicate. Cold GL-3, two-month-old GL-3 plants were treated at 0℃ for 10 h.


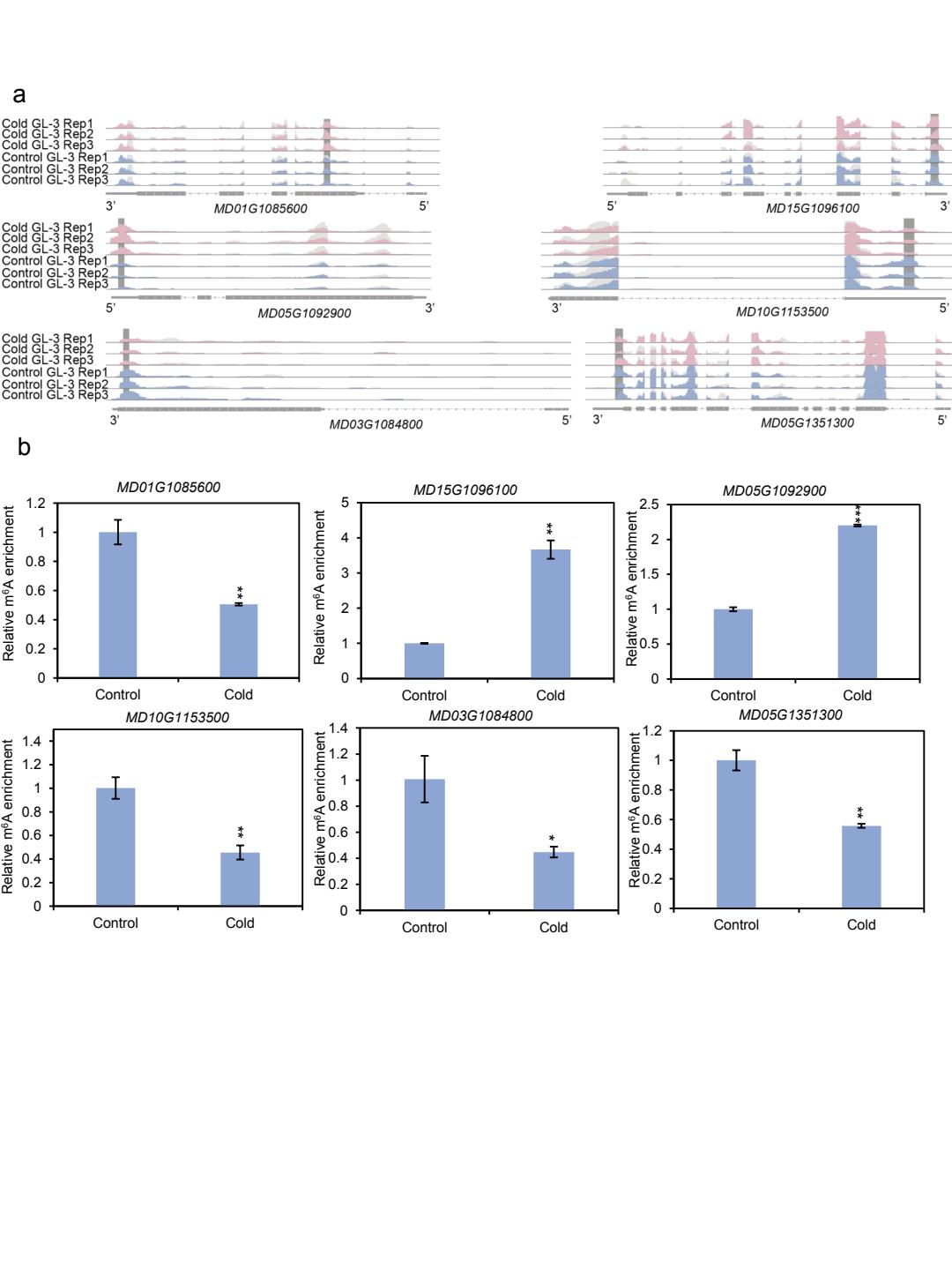


**Supplemental Figure 4.** m^6^A levels of transcripts in GL-3 plants under control and cold conditions. (a) Integrated Genome Viewer displaying the distribution of m^6^A reads in m^6^A-peak-containing transcripts under control and cold conditions in GL-3 plants. The gray rectangles indicate the positions where m^6^A peaks significantly changed in GL-3 plants after cold treatment compared to control conditions. (b) m^6^A-IP-qPCR results of transcripts under control and cold conditions in GL-3 plants. Control, two-month-old GL-3 plants were grown at 22℃. Cold, two-month-old GL-3 plants were treated at 0℃ for 10 h. The asterisks indicate significant differences between the GL-3 and transgenic lines based on Tukey’s test (**P* < 0.05; ***P* < 0.01; ****P* < 0.001). The error bars indicate standard deviations (n = 3 in b).


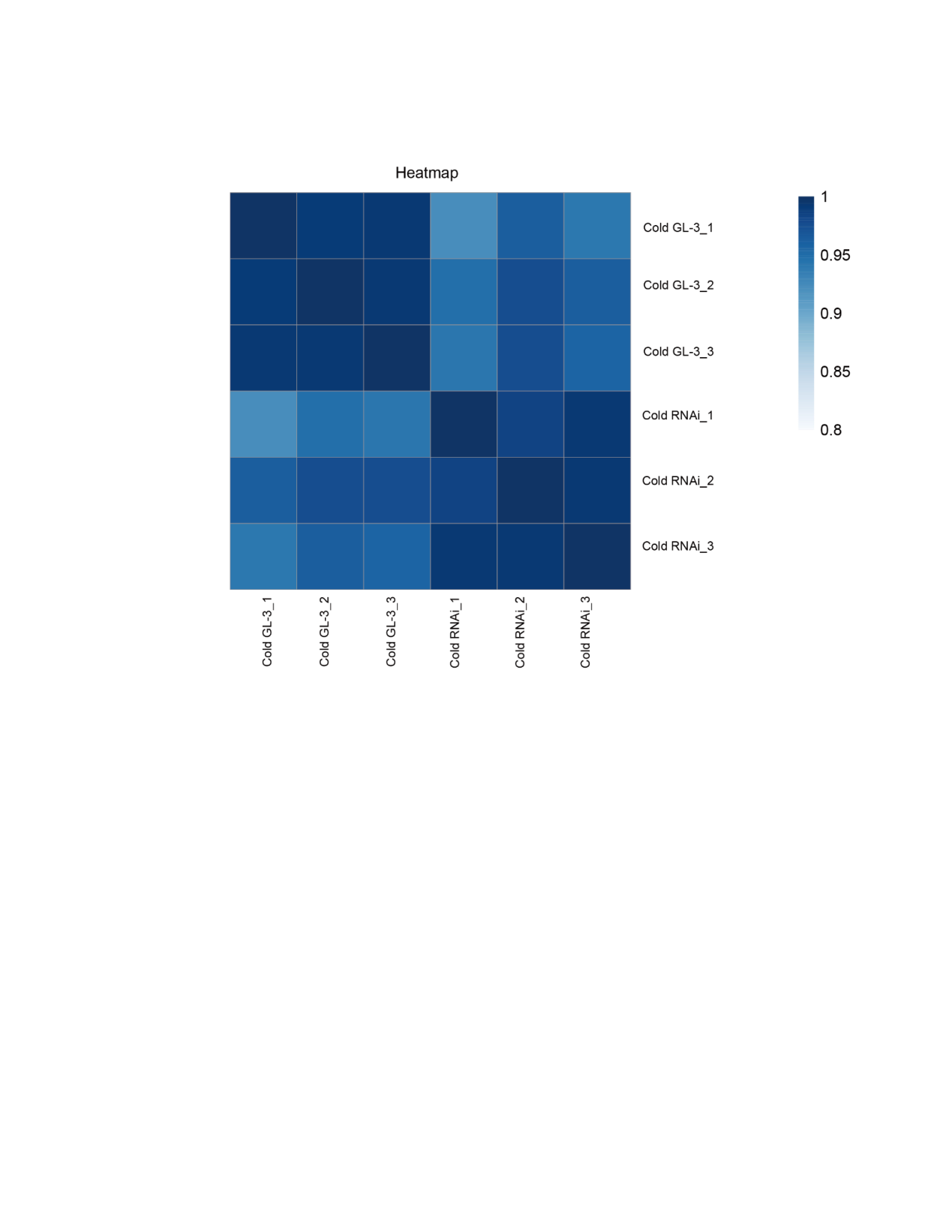


**Supplemental Figure 5.** Pearson correlation analysis of transcripts identified from RNA-seq in GL-3 and *MdMTA* RNAi transgenic plants under cold conditions. Cold GL-3, two-month-old GL-3 plants were treated at 0℃ for 10 h. Cold RNAi, two-month-old *MdMTA* RNAi plants were treated at 0℃ for 10 h.


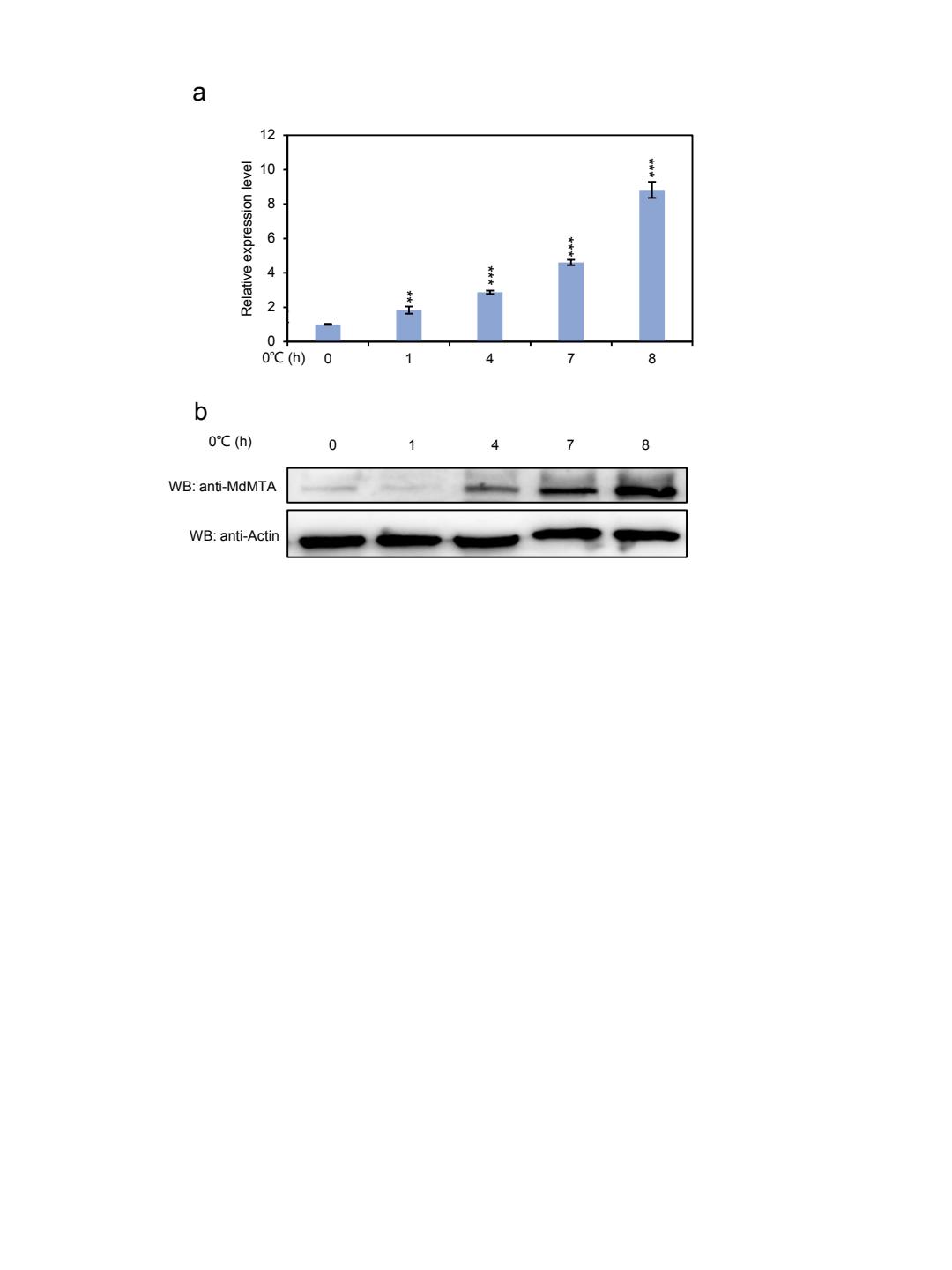


**Supplemental Figure 6.** MdMTA responds to cold stress in apple. (a) The expression level of *MdMTA* in GL-3 plants under cold conditions. *MdMDH* was used as an internal control. (b) The protein level of MdMTA in GL-3 plants under cold treatment. Two-month-old GL-3 plants were treated at 0℃ for different durations, then total proteins were analyzed by western blot with anti-MdMTA antibody. Actin served as the loading control. The asterisks indicate significant differences between the 0 h and other treatment durations based on Tukey’s test (***P* < 0.01; ****P* < 0.001). The error bars indicate standard deviations (n = 3 in a).


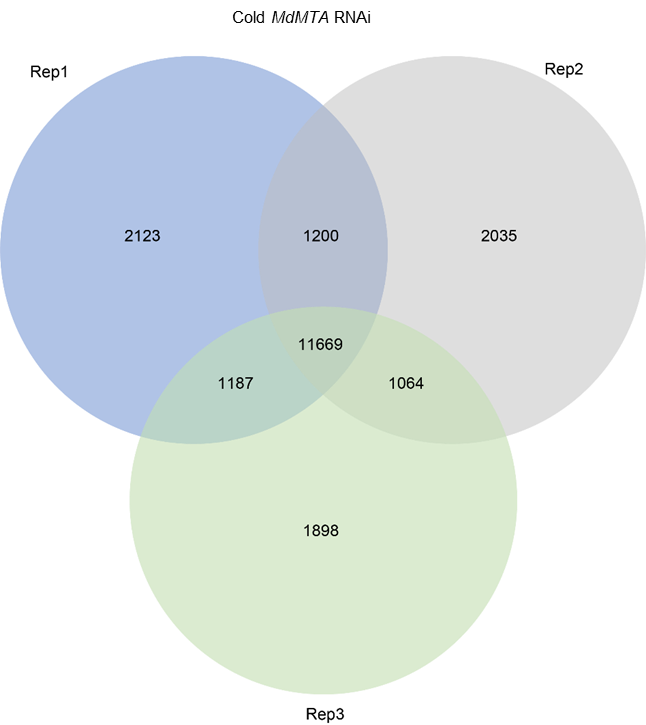


**Supplemental Figure 7.** Venn diagram showing the overlap of three independent m^6^A-seq experiments on *MdMTA* RNAi cold treatment groups. Rep, replicate. Cold *MdMTA* RNAi, two-month-old *MdMTA* RNAi plants were treated at 0℃ for 10 h.


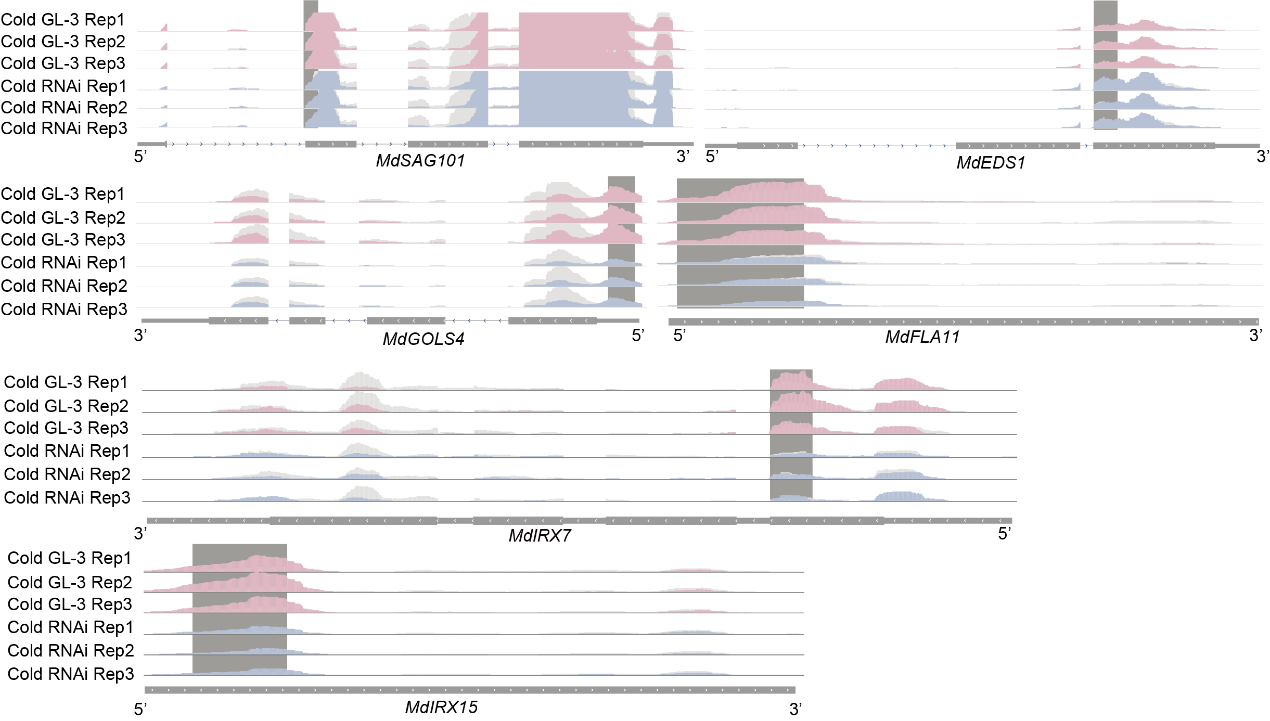


**Supplemental Figure 8.** Integrated Genome Viewer displaying the m^6^A-seq read distributions in *MdSAG101, MdEDS1, MdGOLS4, MdFLA11, MdIRX7,* and *MdIRX15* in GL-3 and *MdMTA* RNAi plants under cold conditions. The gray rectangles indicate the positions where m^6^A peaks significantly changed in *MdMTA* RNAi plants compared to GL-3 plants under cold conditions. Two-month-old plants treated at 0℃ for 10 h were used for m^6^A-seq.


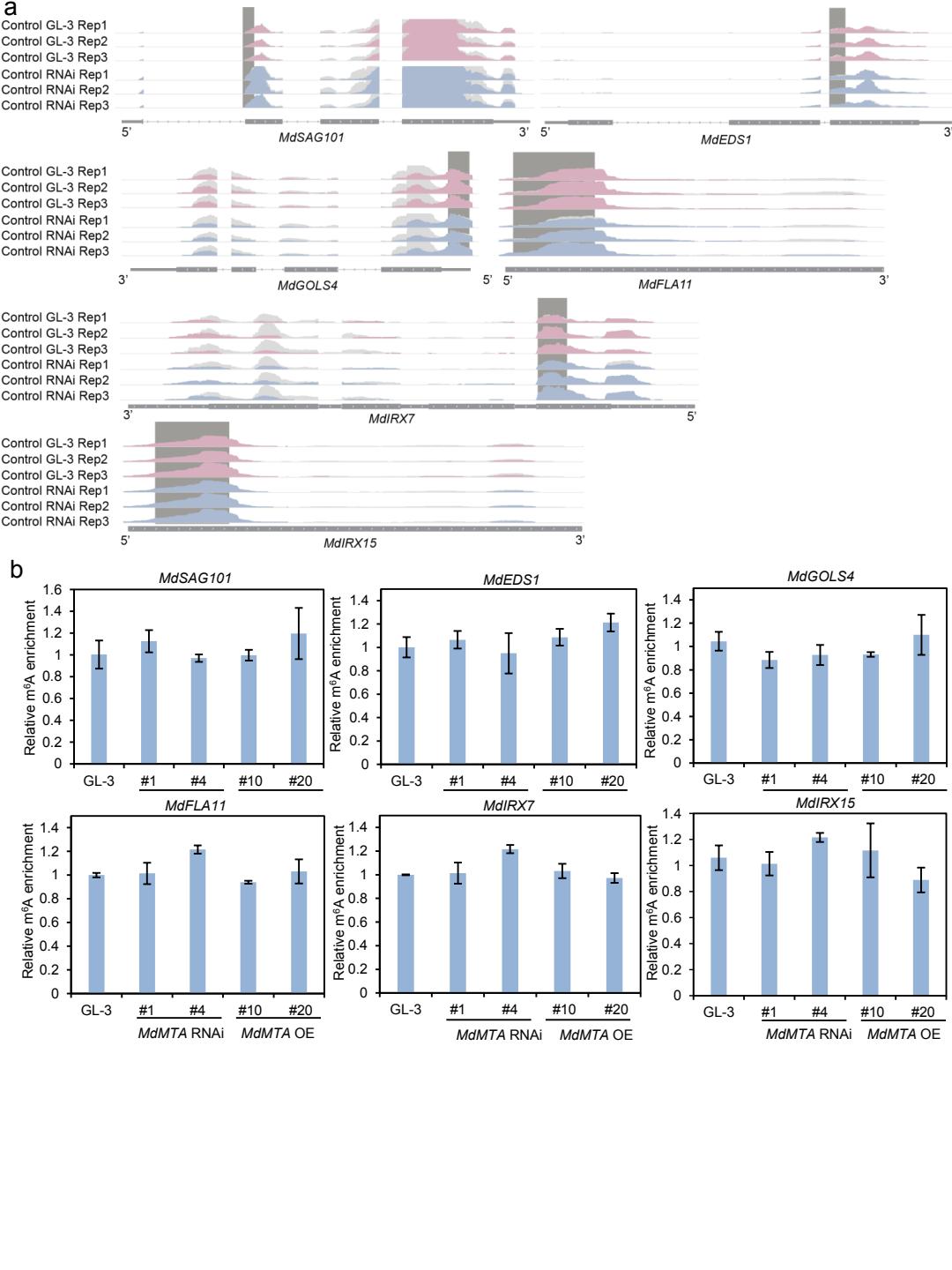


**Supplemental Figure 9.** MdMTA did not mediate m^6^A enrichment of transcripts involved in ROS scavenging and the deposition of cellulose and hemicellulose under control conditions. (a) Integrated Genome Viewer displaying the m^6^A-seq read distributions in *MdSAG101, MdEDS1, MdGOLS4, MdFLA11, MdIRX7,* and *MdIRX15* in GL-3 and *MdMTA* RNAi plants under control conditions. The gray rectangles indicate the positions of m^6^A peaks in *MdMTA* RNAi plants and GL-3 plants under control conditions. (b) Validation of the m^6^A enrichment of *MdSAG101, MdEDS1, MdGOLS4, MdFLA11, MdIRX7,* and *MdIRX15* in GL-3 and *MdMTA* RNAi plants under control conditions. Two-month-old plants grown at 22℃ were used for m^6^A level detection. The error bars indicate standard deviations (n = 3).


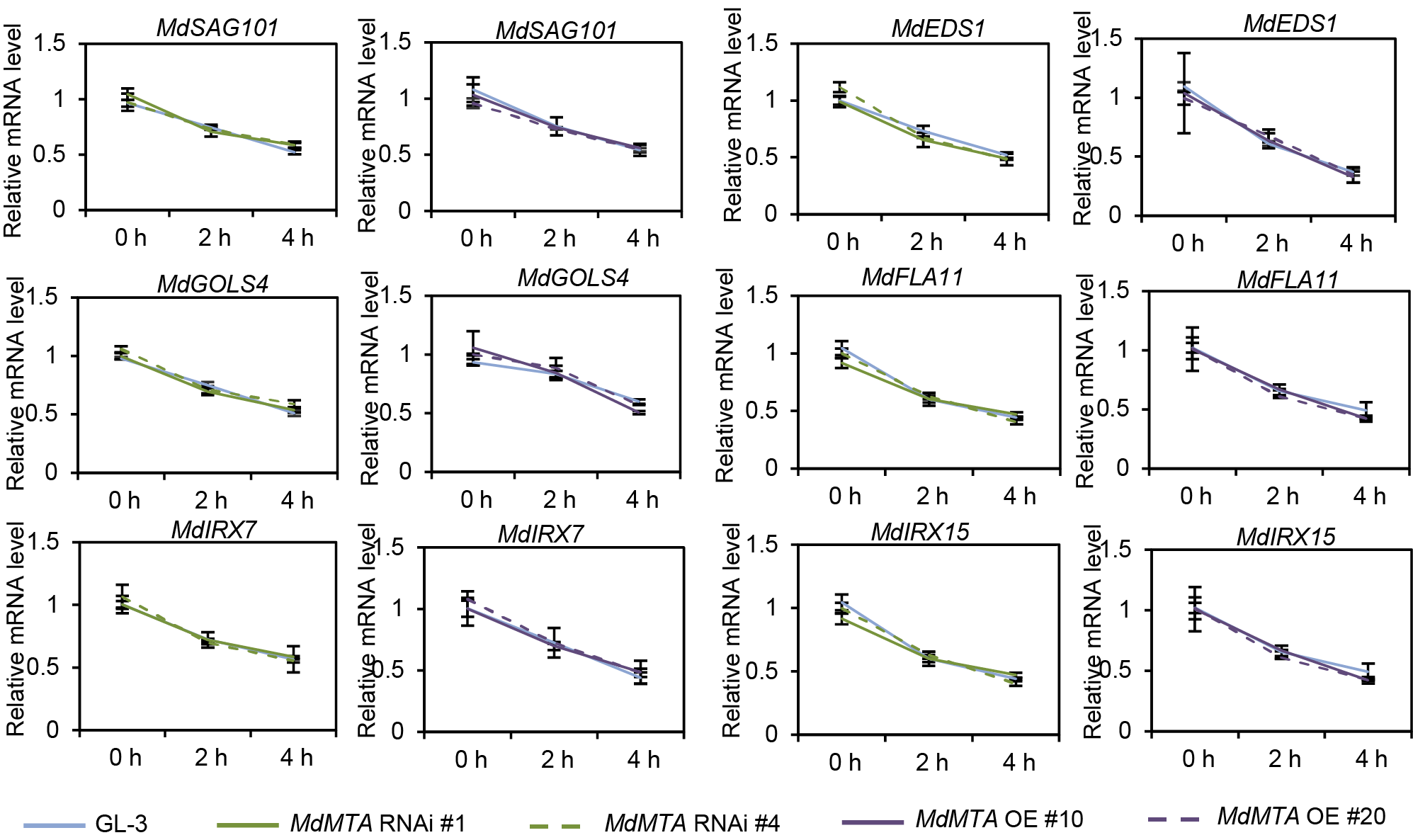


**Supplemental Figure 10.** mRNA stability of the transcripts involved in ROS detoxification and deposition of cellulose and hemicellulose in GL-3 and *MdMTA* transgenic plants under control conditions. Two-month-old plants were treated with 10 μM DMSO for 0, 2, and 4 h at 22℃. Samples at different times were collected for qRT-PCR. The error bars indicate standard deviations (n = 3).


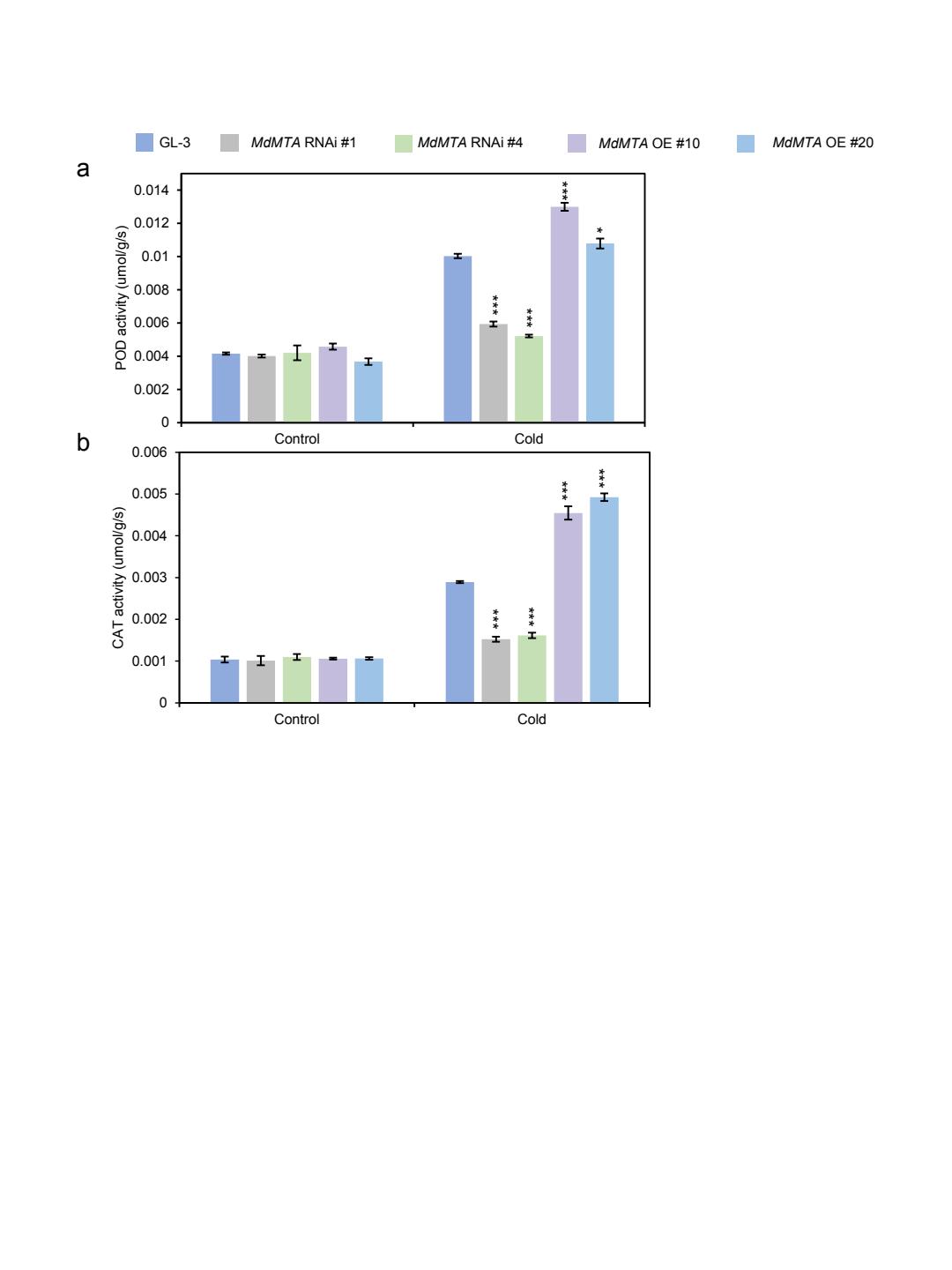


**Supplemental Figure 11.** Enzyme activities in GL-3 and *MdMTA* transgenic plants. (a) POD and (b) CAT enzyme activities in GL-3 and *MdMTA* transgenic plants before and after cold treatment. Two-month-old plants treated at 0℃ for 10 h were used as cold group. The asterisks indicate significant differences between the GL-3 and transgenic plants based on Tukey’s test (**P* < 0.05; ****P* < 0.001). The error bars indicate standard deviations (n = 3 in a and b).

**
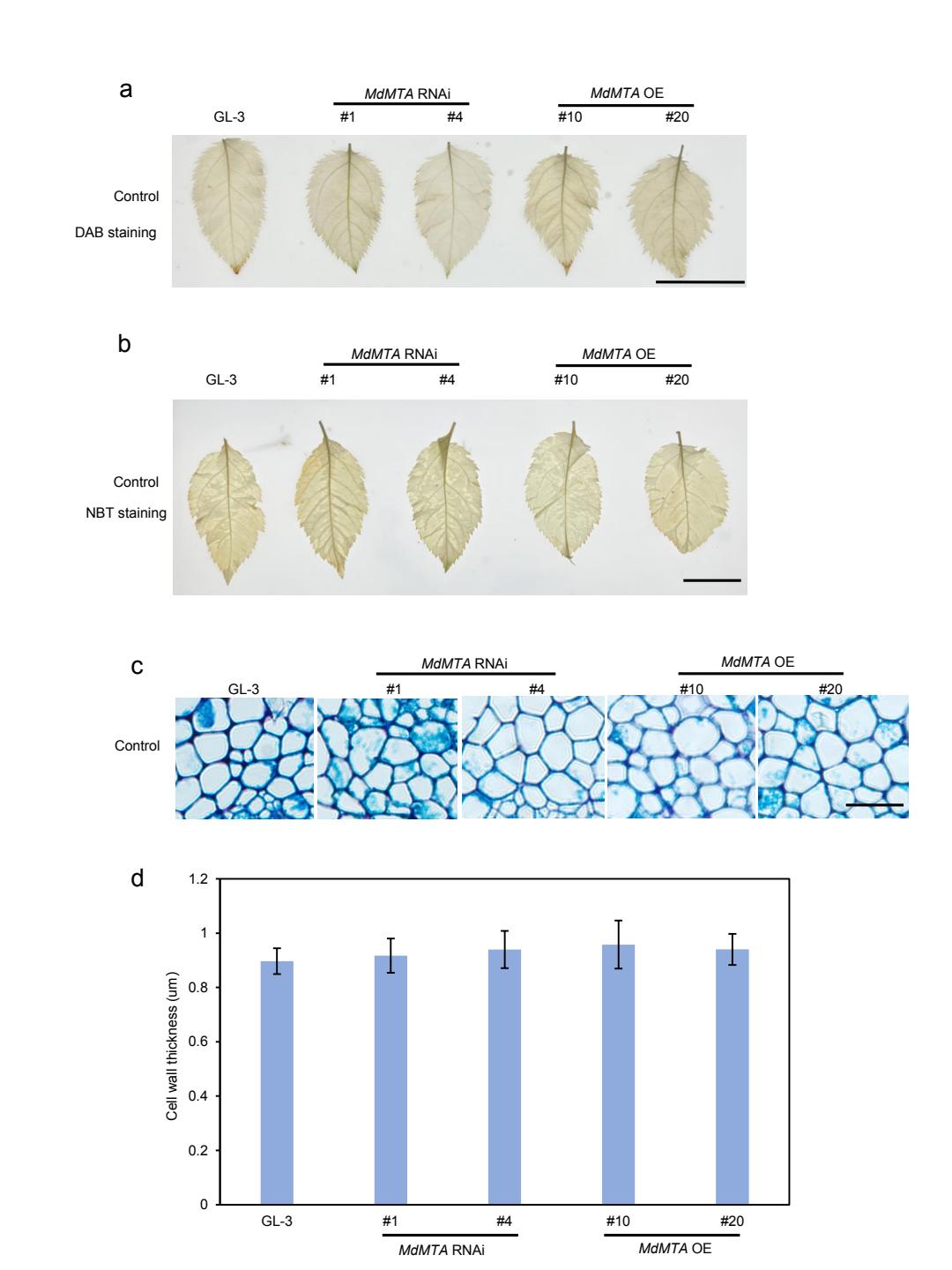
**

**Supplemental Figure 12.** MdMTA did not regulate the ROS scavenging and cell wall deposition under control conditions. (a) DAB and (b) NBT staining of GL-3 and *MdMTA* transgenic plants under control conditions. Bars = 3 cm. (c) Toluidine blue staining of GL-3 and *MdMTA* transgenic plants grown at 22℃. Bar = 20 μm. (d) Cell wall thickness of apple leaves under control conditions. Cell wall thickness was measured using ImageJ software. Two-month-old plants grown at 22℃ were used for DAB, NBT, and toluidine blue staining. The error bars indicate standard deviations (n = 7 in d). DAB, 3, 3’-diaminobenzidine; NBT, nitroblue tetrazolium.


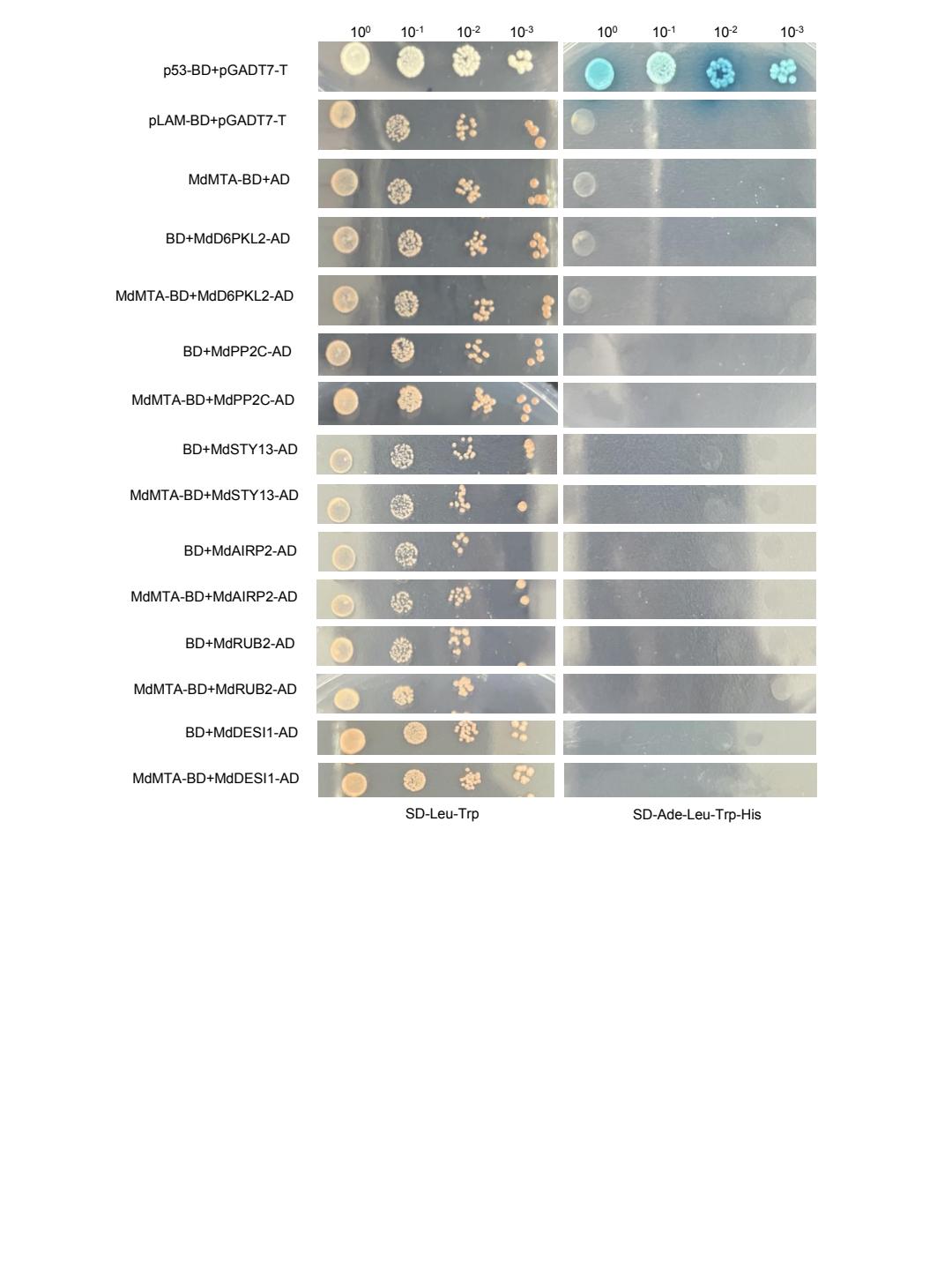


**Supplemental Figure 13.** Yeast two-hybrid results between MdMTA and proteins screened by Y2H library screening. Accession numbers are as follows: MdD69KL2 (XM_029099890), MdPP2C (XM_008356768), MdSTY13 (XM_008376236.3), MdAIRP2 (XM_008386591), MdRUB2 (XM_029092068), and MdDESI1 (XM_029102524).


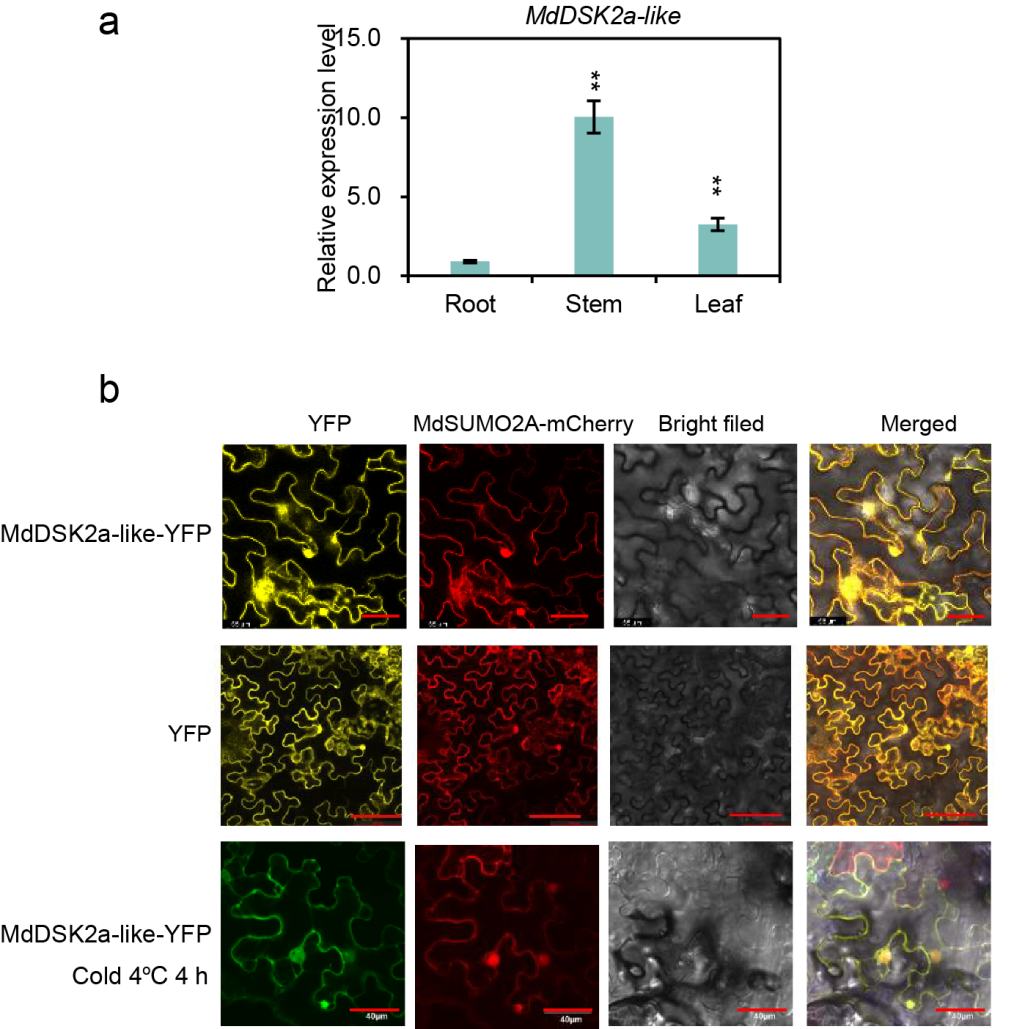


**Supplemental Figure 14.** MdDSK2a-like expression and protein localization. (a) Tissue-specific expression level of *MdDSK2a-like*. The asterisks indicate significant differences between the root and other tissues based on Tukey’s test (***P* < 0.01). (b) Subcellular localization of MdDSK2a-like under control and cold conditions. Bars = 50 μm. The error bars indicate standard deviations (n = 3 in a).


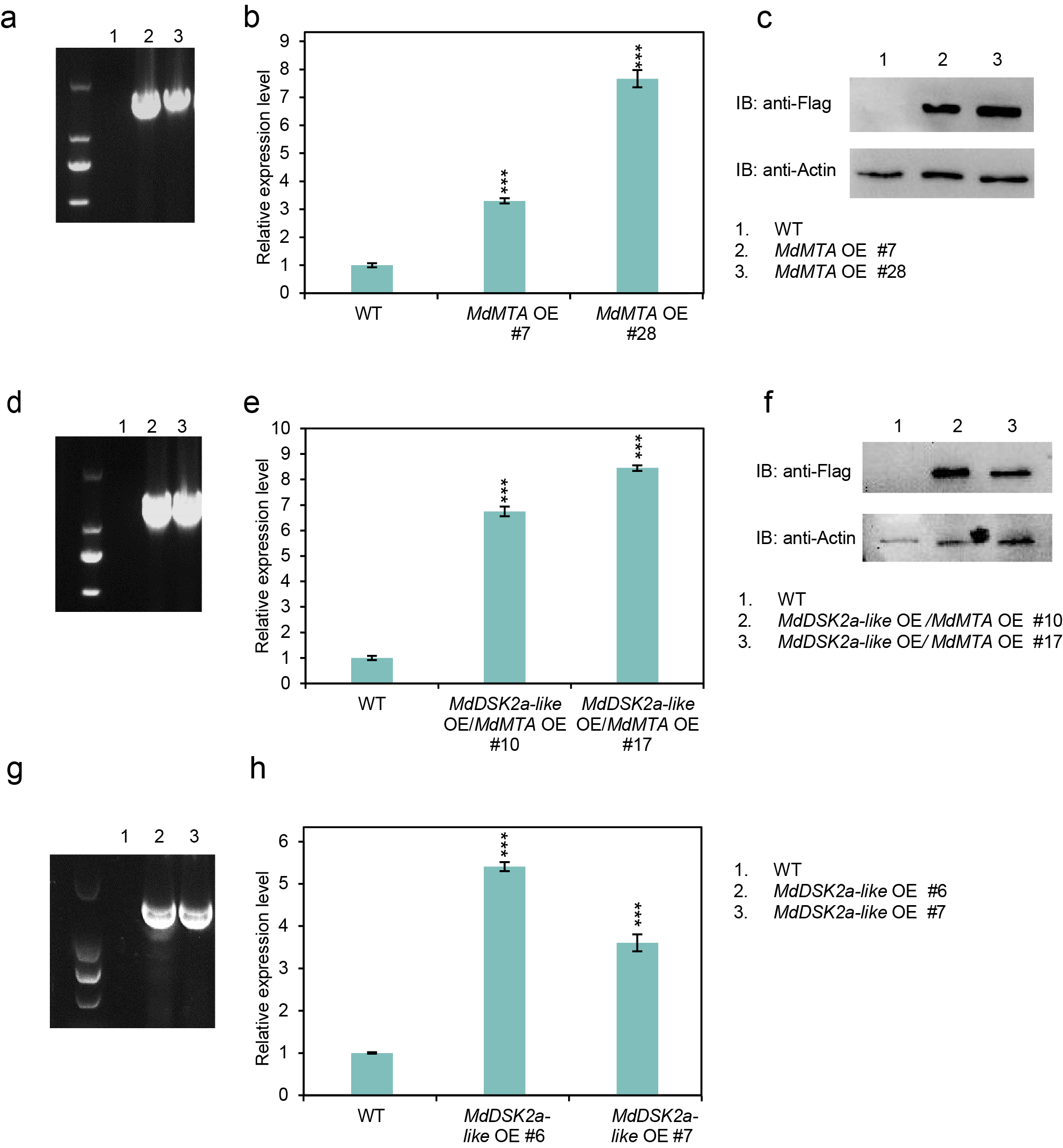


**Supplemental Figure 15.** Identification of the transgenic calli. (a) DNA, (b) RNA, and (c) protein level detection of *MdMTA* OE calli. (d) DNA, (e) RNA, and (f) protein level detection of *MdDSK2a-like* OE/*MdMTA* OE calli. (g) DNA and (h) RNA level detection of *MdDSK2a-like* OE calli. The asterisks indicate significant differences between the WT and transgenic plants based on Tukey’s test (****P* < 0.001). The error bars indicate standard deviations (n = 3 in b, e, and h). WT, wild type.


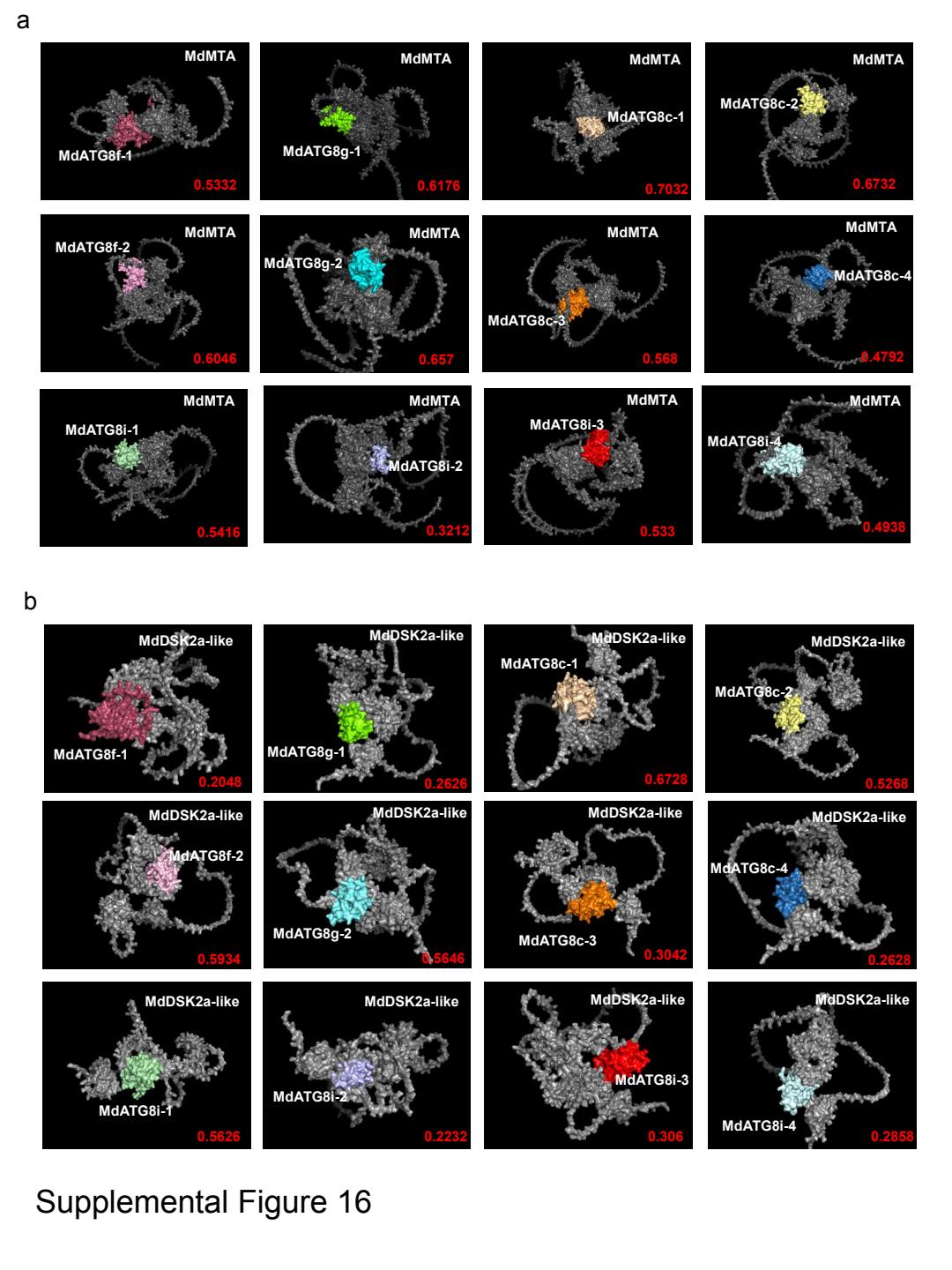


**Supplemental Figure 16.** Best structures predicted by AlphaFold of MdMTA/MdDSK2a-like and MdATG8s. (a) AlphaFold-predicted models of MdMTA and MdATG8s. MdMTA is shown in gray. MdATG8s are shown in different colors. (b) AlphaFold-predicted models of MdDSK2a-like and MdATG8s. MdDSK2a-like is shown in gray. The red numbers in the lower right corner represent values of 0.8 ipTM + 0.2 pTM, which is greater than 0.75 indicating that the two proteins are likely to interact. Accession numbers are as follows: MdATG8f-1 (MD00G1114800), MdATG8f-2 (MD06G1061100), MdATG8g-1 (MD02G1210100), MdATG8g-2 (MD07G1116100), MdATG8c-1 (MD11G1216100), MdATG8c-2 (MD05G1347700), MdATG8c-3 (MD10G1322100), MdATG8c-4 (MD03G1199200), MdATG8i-1 (MD16G1138400), MdATG8i-2 (MD13G1142000),

MdATG8i-3 (MD13G1141800) and MdATG8i-4 (MD09G1293100).


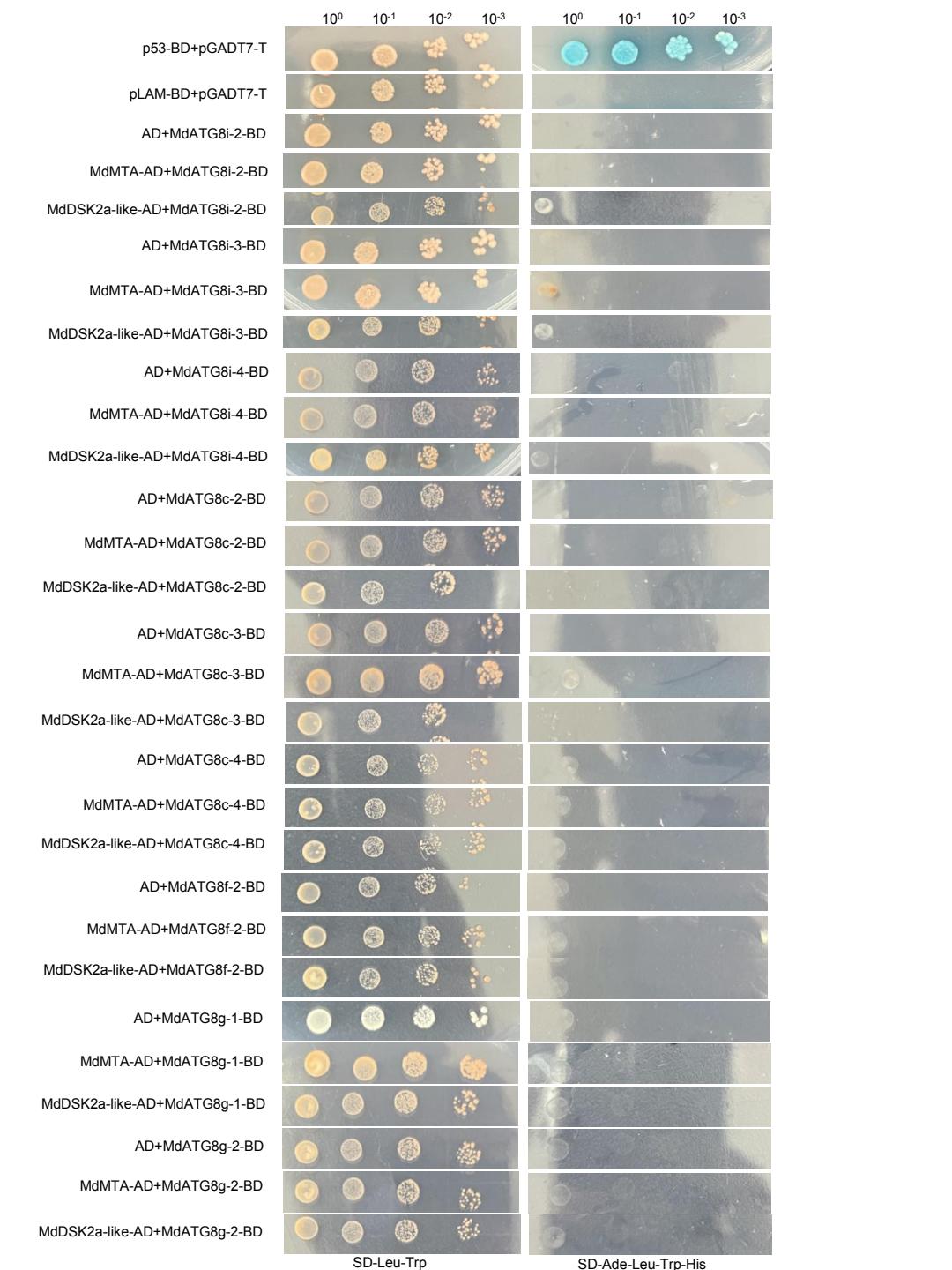


**Supplemental Figure 17.** Yeast two-hybrid results between MdMTA or MdDSK2a-like with MdATG8s.


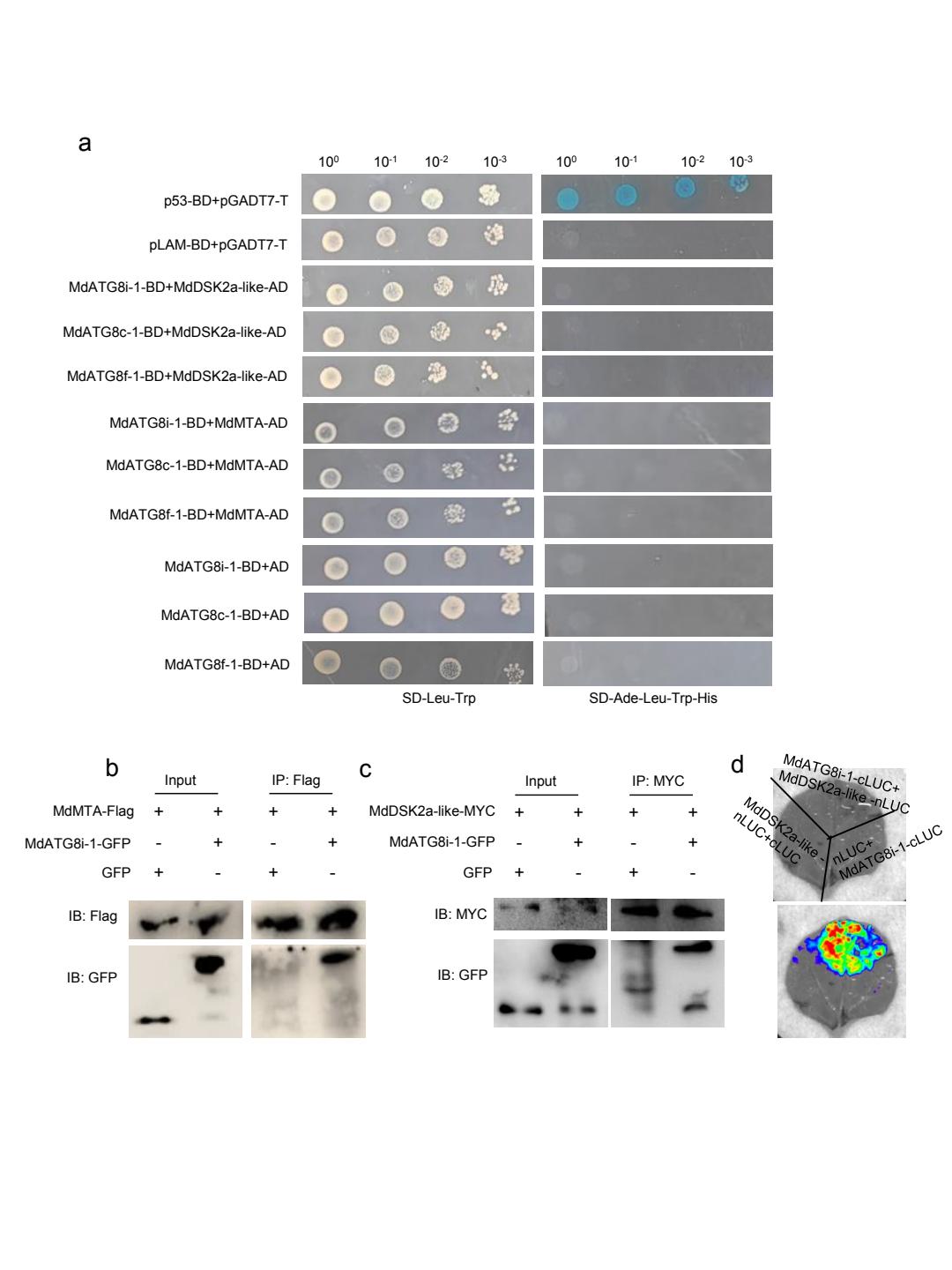


**Supplemental Figure 18.** The interaction between MdATG8i-1 and MdMTA or MdDSK2a-like. (a) Interaction of MdMTA or MdDSK2a-like with MdATG8i-1, MdATG8c-1, or MdATG8f-1 determined by yeast two-hybrid. (b) Co-immunoprecipitation in *N. benthamiana* showing the association of MdMTA with MdATG8i-1. Total proteins were extracted and immunoprecipitation was performed with anti-Flag overnight. Western blot was performed with anti-Flag or anti-GFP antibodies. (c) Co-immunoprecipitation in *N. benthamiana* showing the association of MdDSK2a-like with MdATG8i-1. Total proteins were extracted and immunoprecipitation was performed with anti-MYC overnight. Western blot was performed with anti-MYC or anti-GFP antibodies. (d) Split-LUC analysis showing the interaction of MdDSK2a-like and MdATG8i-1.


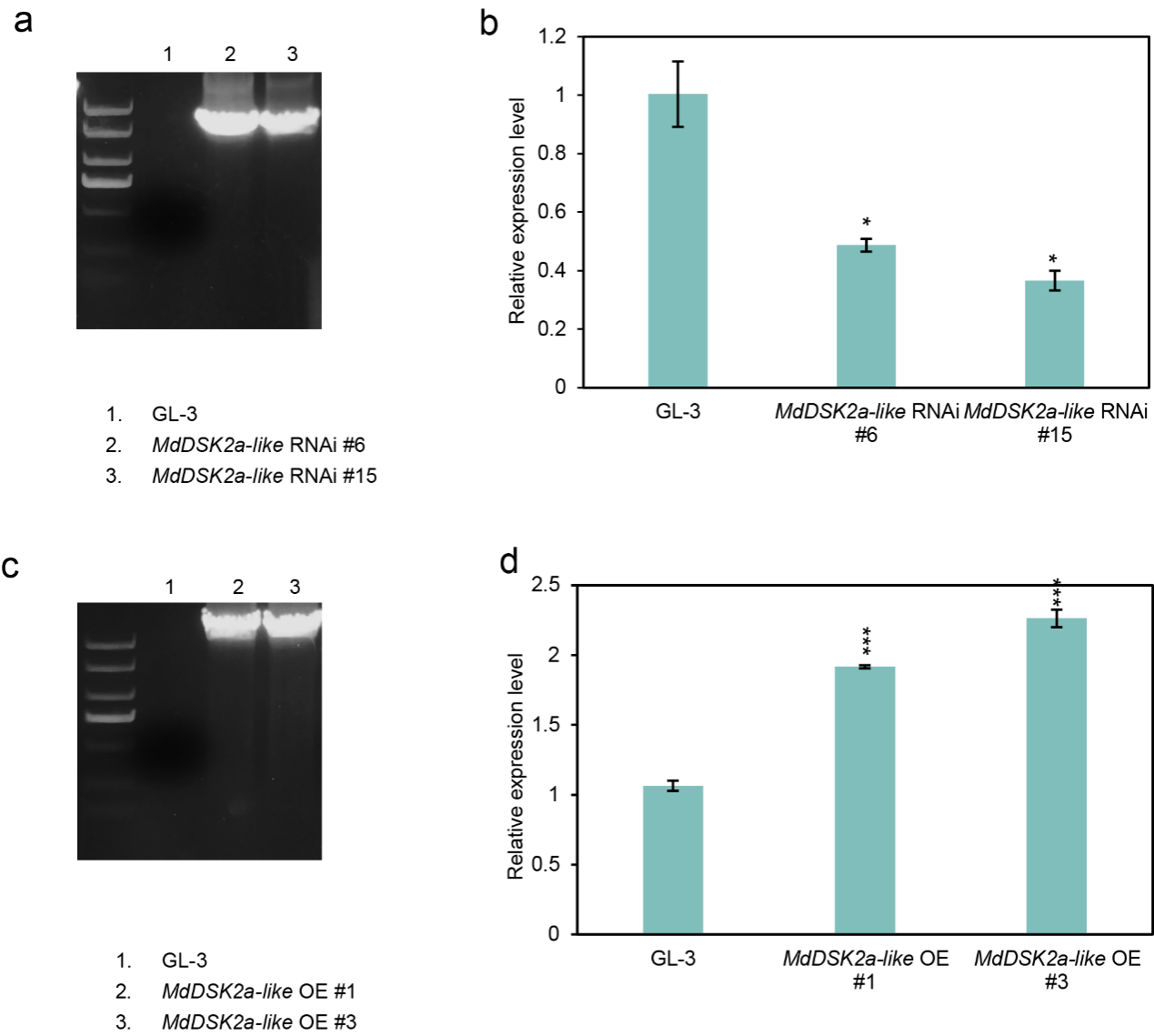


**Supplemental Figure 19.** Identification of *MdDSK2a-like* transgenic plants. (a) DNA and (b) RNA level detection of *MdDSK2a-like* RNAi plants. (c) DNA and (d) RNA level detection of *MdDSK2a-like* OE plants. The asterisks indicate significant differences between the GL-3 and transgenic plants based on Tukey’s test (**P* < 0.05; ****P* < 0.001). The error bars indicate standard deviations (n = 3 in b and d).


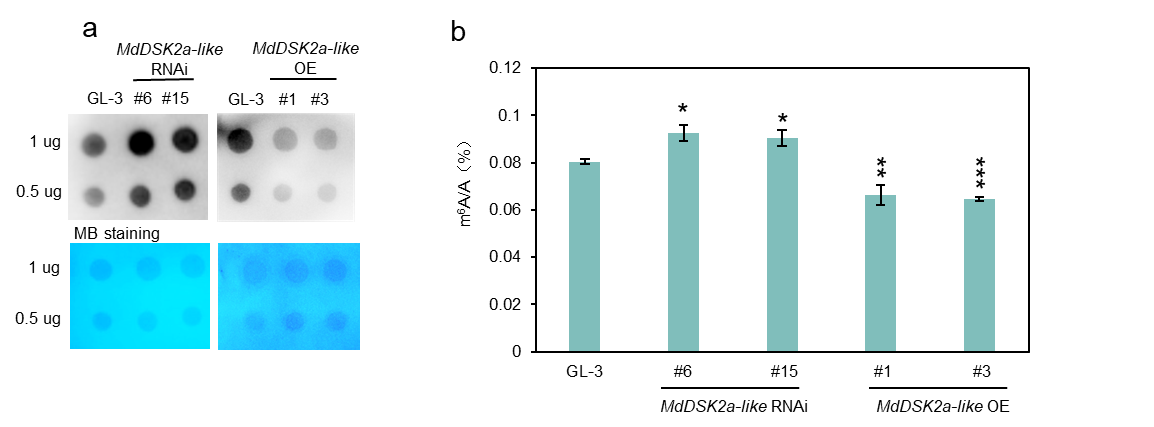


**Supplemental Figure 20.** m^6^A levels in *MdDSK2a-like* transgenic plants under control conditions. (a) m^6^A signal in GL-3 and *MdDSK2a-like* transgenic plants under control conditions. MB staining: Methylene blue staining of RNA. (b) LC-MS/MS detection of m^6^A levels in GL-3 and *MdDSK2a-like* transgenic plants under control conditions. Two-month-old plants grown at 22℃ were used for dot-blot and LC-MS/MS. The asterisks indicate significant differences between the GL-3 and *MdDSK2a-like* transgenic plants based on Tukey’s test (**P* < 0.05; ***P* < 0.01; ****P* < 0.001). The error bars indicate standard deviations (n = 3 in b).


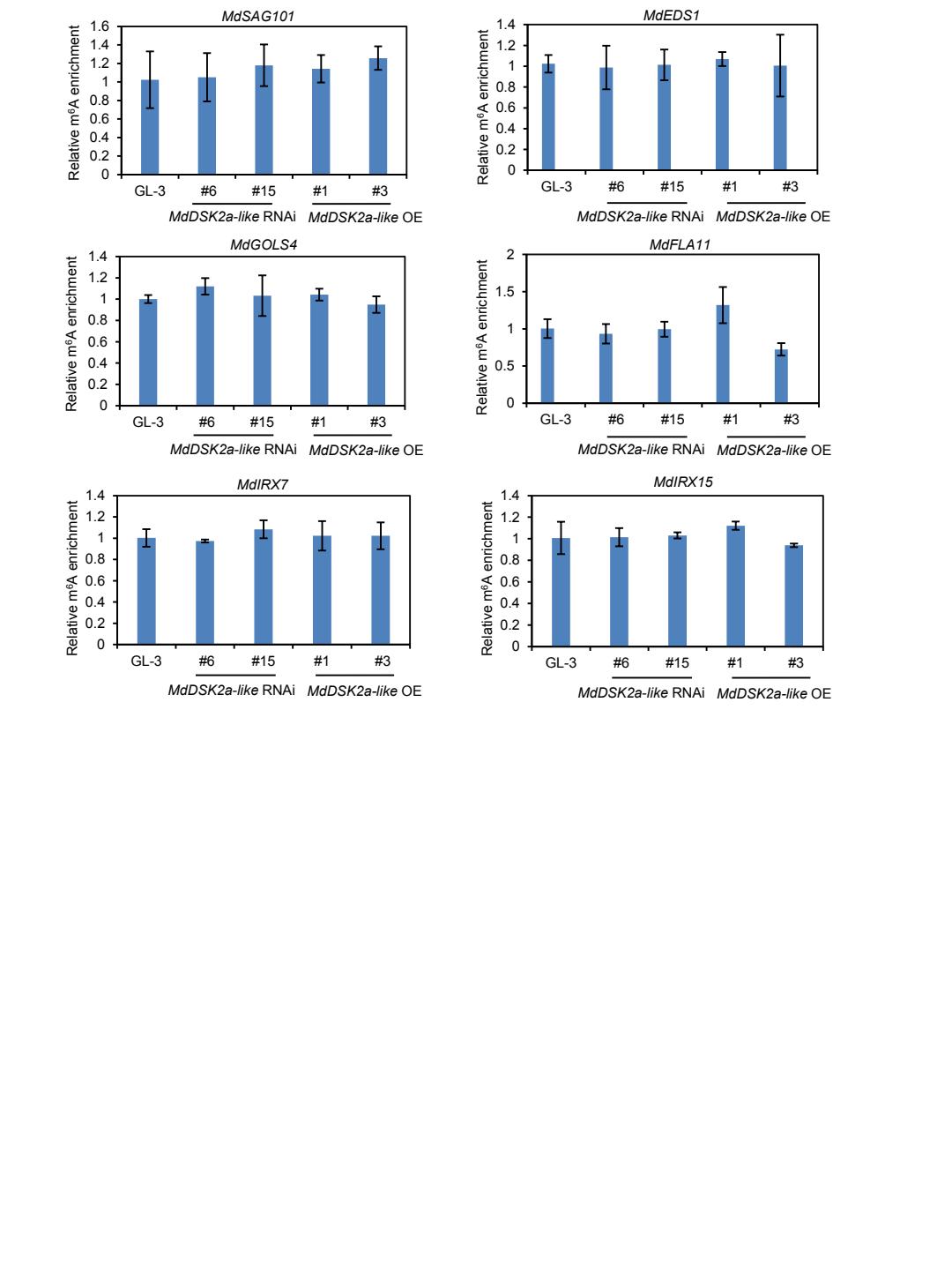


**Supplemental Figure 21.** Validation of the m^6^A enrichment of *MdSAG101, MdEDS1, MdGOLS4, MdFLA11, MdIRX7,* and *MdIRX15* in GL-3 and *MdDSK2a-like* transgenic plants under control conditions. Two-month-old plants grown at 22℃ were used for m^6^A-IP-qPCR. The error bars indicate standard deviations (n = 3).


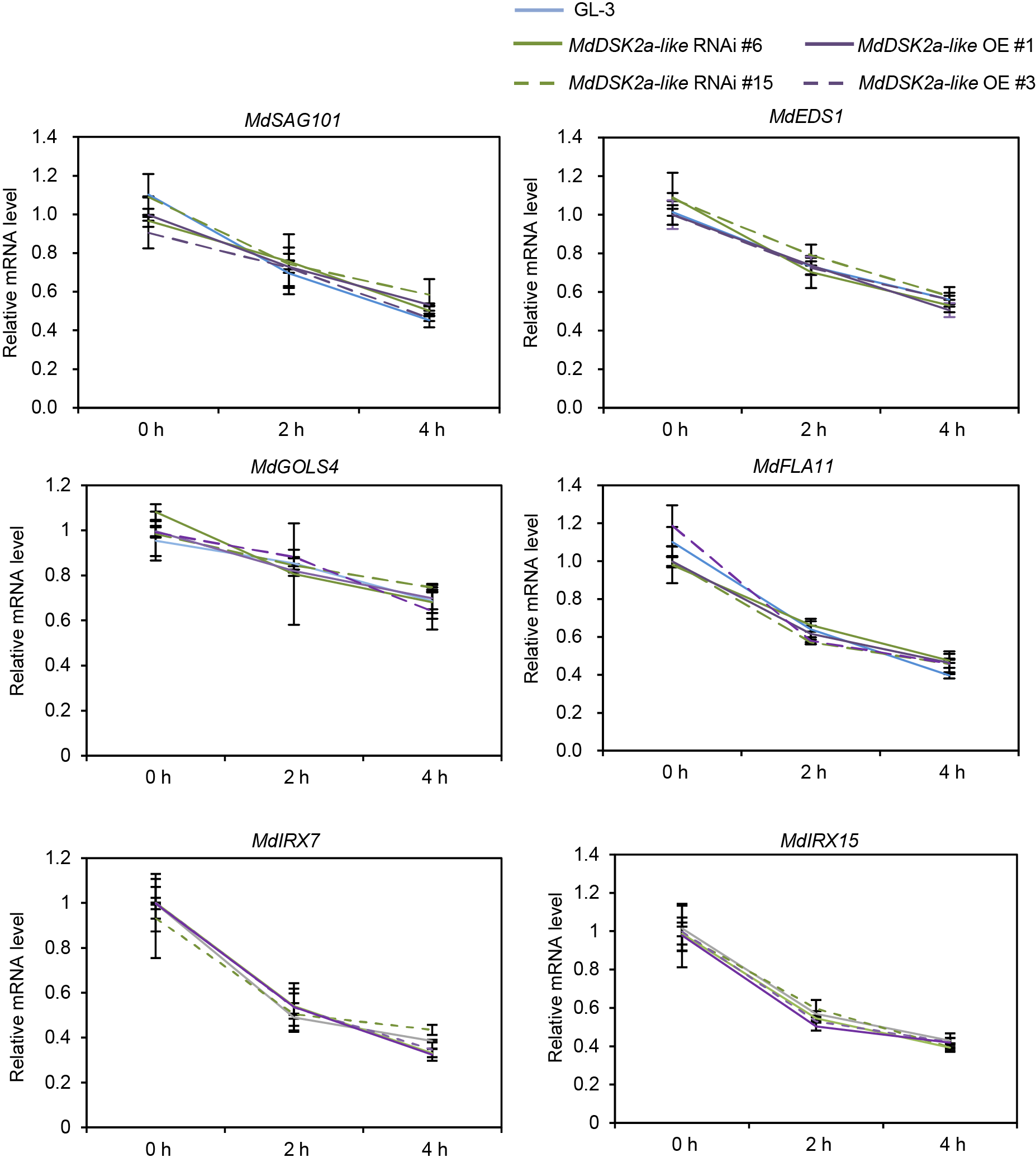


**Supplemental Figure 22.** mRNA stability of the transcripts involved in ROS scavenging and the deposition of cellulose and hemicellulose in GL-3 and *MdDSK2a-like* transgenic plants under control conditions. Two-month-old plants were treated with 10 μM DMSO for 0, 2, and 4 h at 22℃. Samples at different times were collected for qRT-PCR. The error bars indicate standard deviations (n = 3).


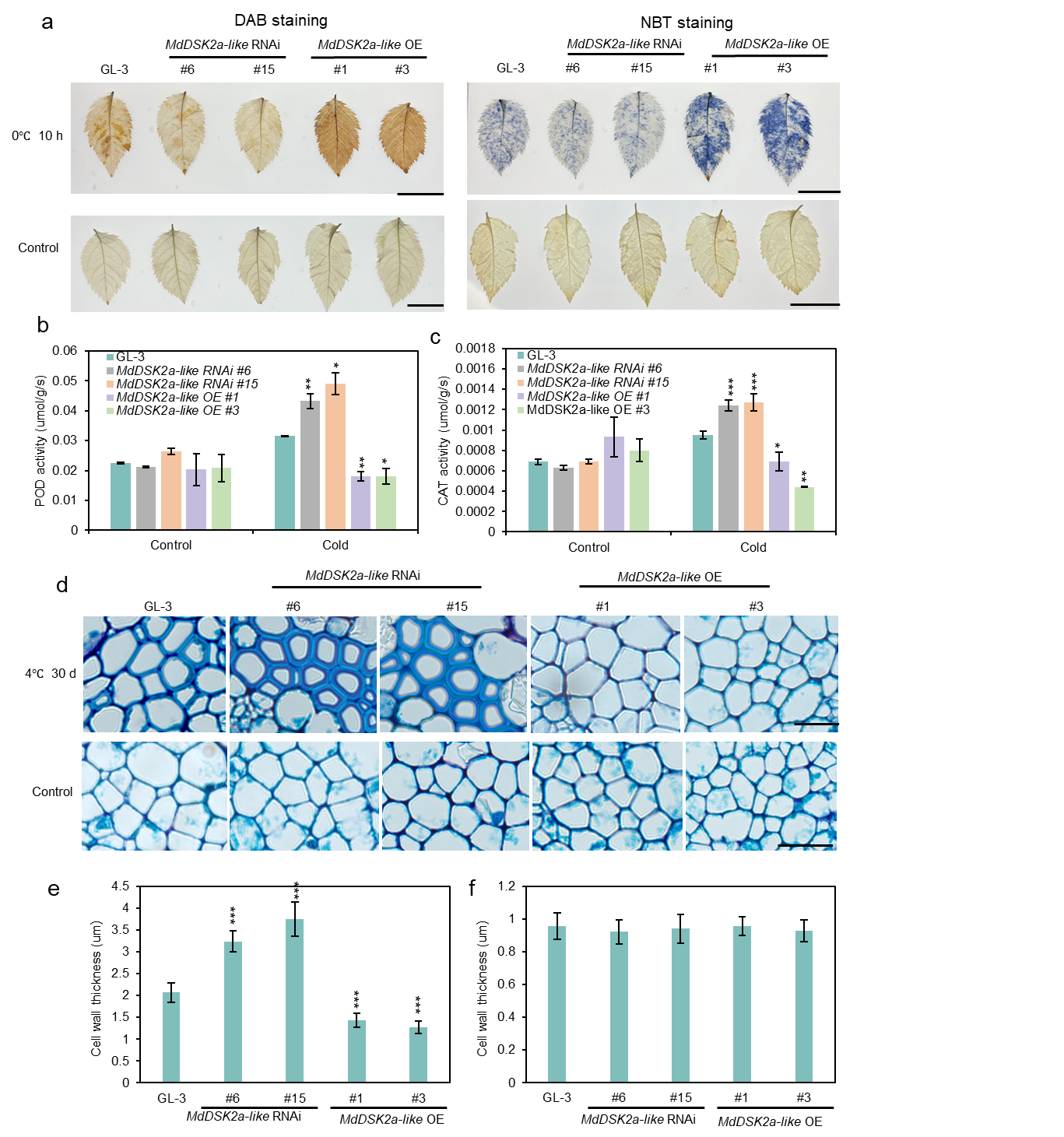


**Supplemental Figure 23.** MdDSK2a-like negatively regulated ROS scavenging and cell wall deposition under cold conditions. (a) DAB and NBT staining in GL-3 and *MdDSK2a-like* transgenic plants under control and cold conditions. Two-month-old plants were treated at 0℃ for 10 h. DAB, 3, 3’-diaminobenzidine; NBT, nitroblue tetrazolium. Bars = 3 cm. (b) POD and (c) CAT enzyme activities in GL-3 and *MdDSK2a-like* transgenic plants before and after cold treatment. Two-month-old plants were treated at 0℃ for 10 h. (d) Toluidine blue staining of GL-3 and *MdDSK2a-like* transgenic plants. Two-month-old plants were treated at 4℃ for one month or grown at 22℃. Bars = 20 μm. Cell wall thickness of apple leaves under (e) cold and (f) control conditions. Cell wall thickness was measured using ImageJ software. The asterisks indicate significant differences between the GL-3 and transgenic lines based on Tukey’s test (**P* < 0.05; ***P* < 0.01; ****P* < 0.001). The error bars indicate standard deviations (n = 3 in b, c; 7 in e, f).


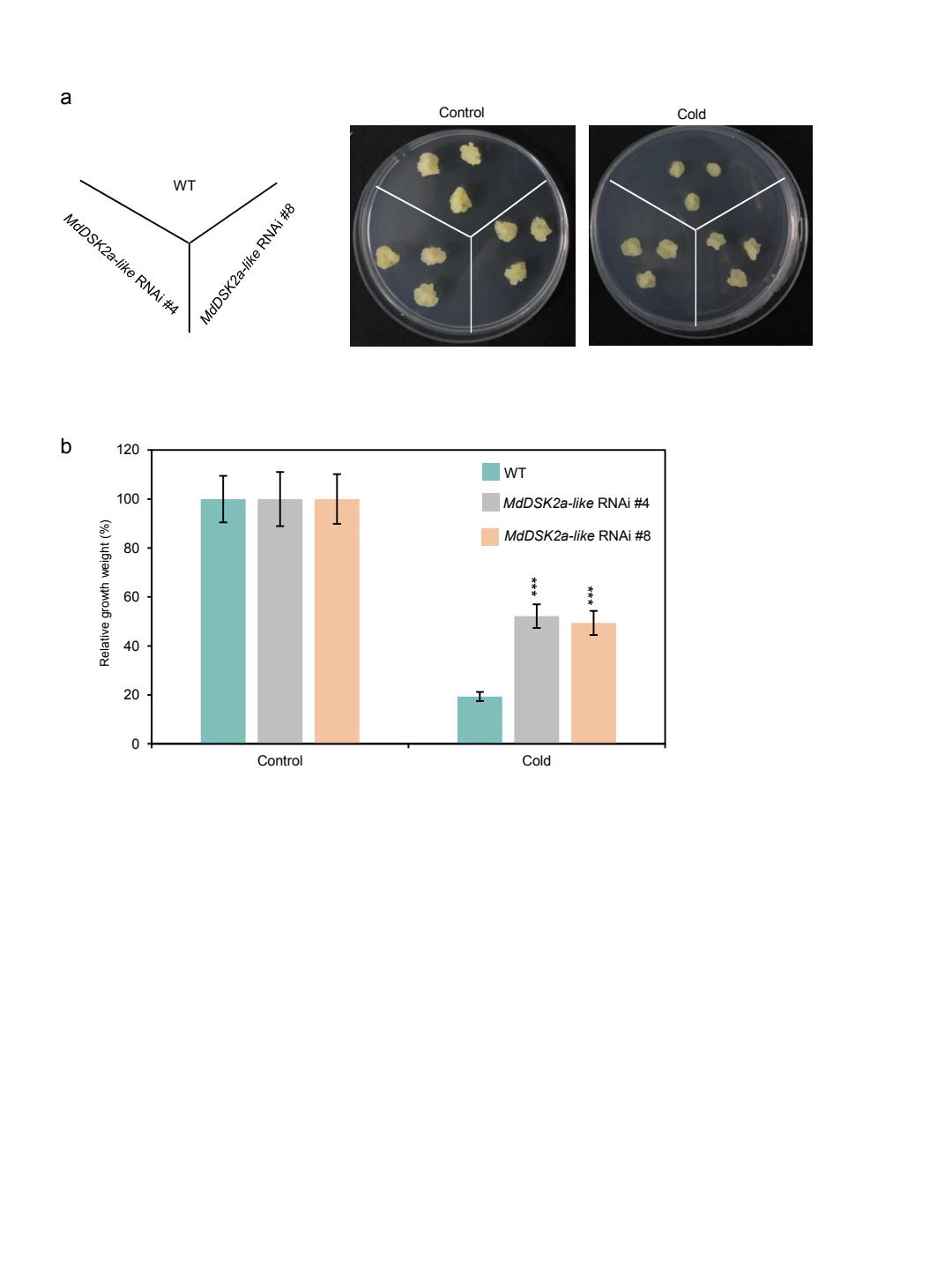


**Supplemental Figure 24.** MdDSK2a-like negatively regulated cold tolerance in calli. (a) Phenotype of the wild type and *MdDSK2a-like* RNAi transgenic calli under control or cold conditions. Control conditions, transgenic calli were cultured at 22℃ for two weeks. Cold conditions, transgenic calli were cultured at 22℃ for one week and then transferred to 4℃ for additional one week. (b) Relative growth weight of wild type and *MdDSK2a-like* RNAi transgenic calli under control and cold conditions. The asterisks indicate significant differences between the WT and transgenic plants based on Tukey’s test (****P* < 0.001). The error bars indicate standard deviations (n = 6 in b). WT, wild type.


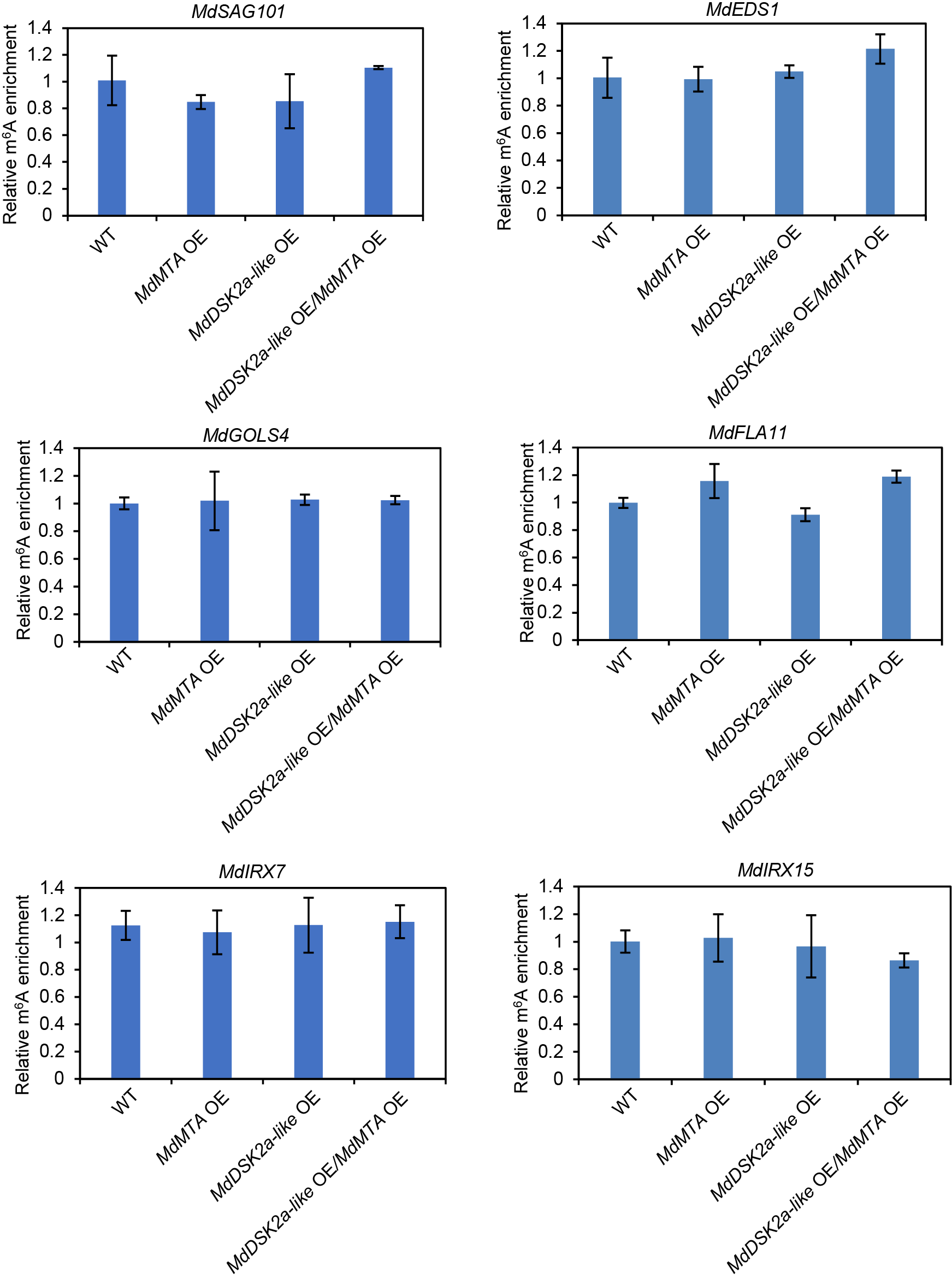


**Supplemental Figure 25.** Validation of the m^6^A enrichment of *MdSAG101, MdEDS1, MdGOLS4, MdFLA11, MdIRX7,* and *MdIRX15* in transgenic calli under control conditions. Two-week-old WT and transgenic calli were used for m^6^A-IP-qPCR. WT, wild type.

**
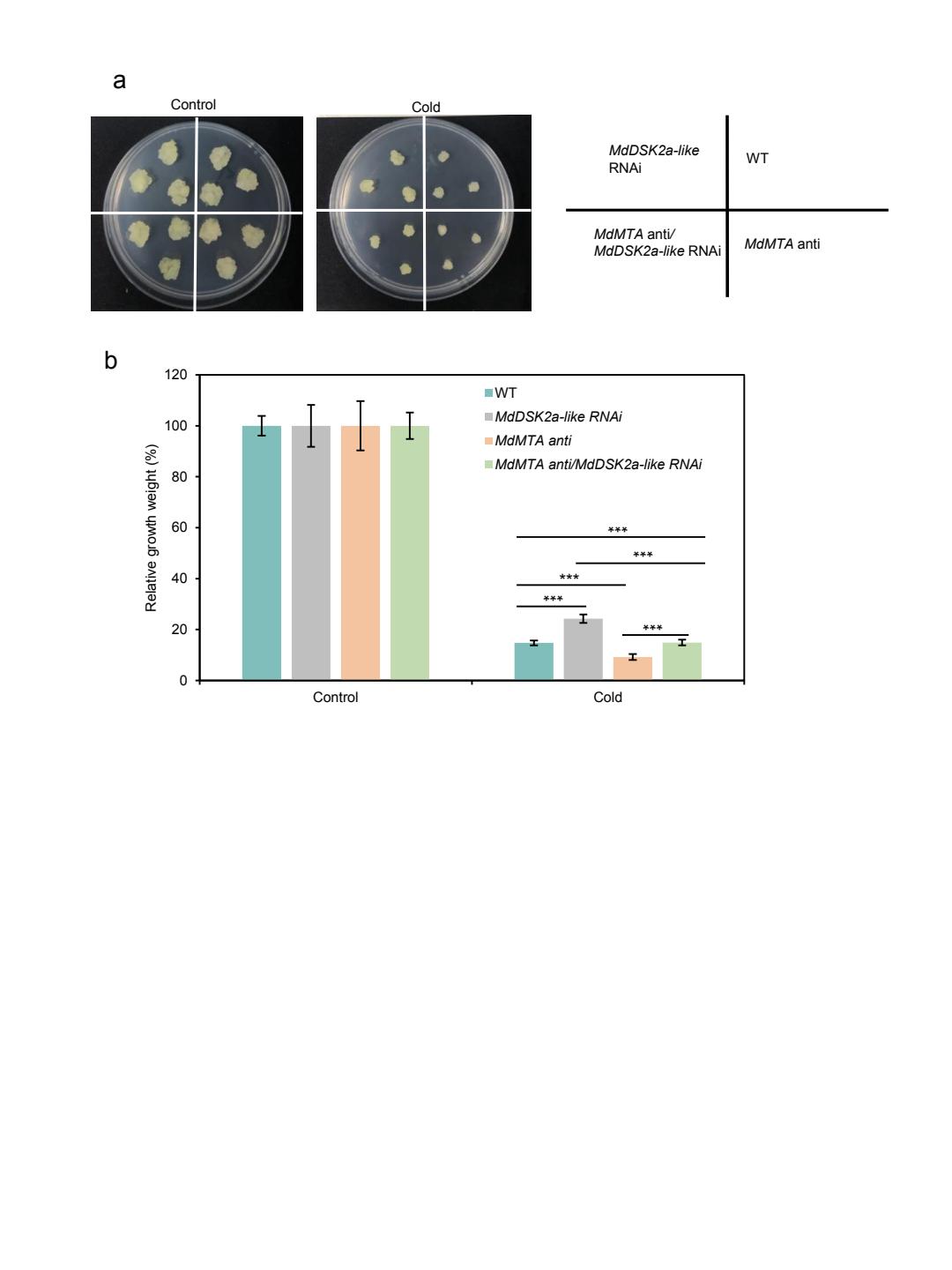
**

**Supplemental Figure 26.** Genetic interaction of MdMTA and MdDSK2a-like. (a) Phenotype of the wild type, *MdDSK2a-like* RNAi, *MdMTA* anti, and *MdMTA* anti/*MdDSK2a-like* RNAi transgenic calli under control or cold conditions. Control conditions, transgenic calli were cultured at 22℃ for two weeks. Cold conditions, transgenic calli were cultured at 22℃ for one week and then transferred to 4℃ for additional one week. (b) Relative growth weight of wild type, *MdDSK2a-like* RNAi, *MdMTA* anti, and *MdMTA* anti/*MdDSK2a-like* RNAi transgenic calli under control or cold conditions. The asterisks indicate significant differences between the WT and transgenic plants based on Tukey’s test (****P* < 0.001). The error bars indicate standard deviations (n = 6 in b). WT, wild type.


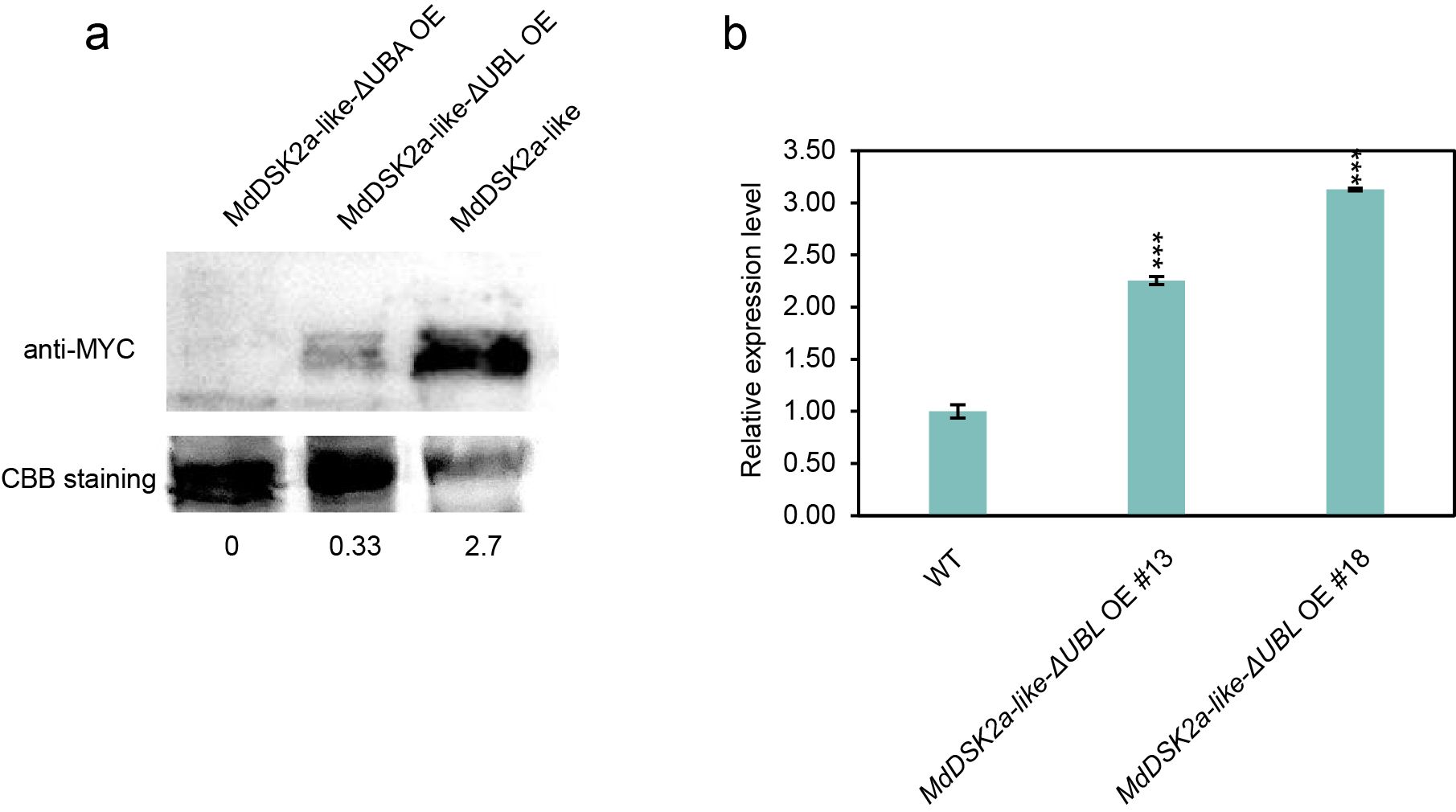


**Supplemental Figure 27.** Effects of UBA and UBL domains on the stability of MdDSK2a-like protein. (a) Protein level detection in *MdDSK2a-like-∆UBL* OE, *MdDSK2a-like-∆UBA* OE, and *MdDSK2a-like* OE calli. (b) RNA level detection of *MdDSK2a-like-∆UBL* OE calli. The asterisks indicate significant differences between the WT and transgenic calli based on Tukey’s test (****P* < 0.001). The error bars indicate standard deviations (n = 3 in c). WT, wild type.

**Table S2. Y2H library screening of MdMTA**

| **NCBI number** | **Annotation** |
| --- | --- |
| **XM_029099890** | **Malus domestica serine/threonine-protein kinase D6PKL2** |
| **XM_029088775.1** | **Malus domestica nudix hydrolase 26, chloroplastic** |
| **XM_008386582** | **Malus domestica FGGY carbohydrate kinase domain-containing protein** |
| **XM_008356768** | **Malus domestica probable protein phosphatase 2C 25** |
| **XM_029106180.1** | **Malus domestica N6-adenosine-methyltransferase non-catalytic subunit MTB** |
| **XM_008376236.3** | **Malus domestica serine/threonine-protein kinase STY13-like** |
| **XM_008375944** | **Malus domestica signal recognition particle subunit SRP68** |
| **NM_001293994** | **Malus domestica RPM1-interacting protein 4 (RIN4-1)** |
| **XM_008392276.3** | **Malus domestica S-adenosylmethionine synthase 2** |
| **XM_008350129** | **Malus domestica transcription factor PIF4-like** |
| **XM_008350991** | **Malus domestica pathogenesis-related genes transcriptional activator PTI5-like** |
| **XM_029104745** | **Malus domestica NAD-dependent malic enzyme 59 kDa isoform, mitochondrial-like** |
| **XM_017332469** | **Malus domestica protein TSS** |
| [**XM_008384205.3**](https://www.ncbi.nlm.nih.gov/nuccore/XM_008384205.3) | **ubiquitin domain-containing protein DSK2a** |
| **XM_008355065** | **Malus domestica 1,2-dihydroxy-3-keto-5-methylthiopentene dioxygenase 1** |
| **XM_008340575.3** | **Malus domestica probable glycerol-3-phosphate acyltransferase 3** |
| **XM_008386591** | **Malus domestica E3 ubiquitin-protein ligase AIRP2** |
| **XM_029092068** | **Malus domestica ubiquitin-NEDD8-like protein RUB2** |
| **XM_008349802** | **Malus domestica phosphoglucan phosphatase LSF1** |
| **XM_008365951** | **Malus domestica uncharacterized** |
| **XM_029108416** | **Malus domestica chaperone protein dnaJ A6, chloroplastic-like** |
| **XM_029102524** | **desumoylating isopeptidase 1 isoform X1 [Malus domestica]** |

**Table. S3 Primer used in this study**

| **Primer for Y2H assay** | |
| --- | --- |
| **MdMTA-BD-F** | **TCAGAGGAGGACCTGCATATGATGGAGACCCAATCGGAAGGC** |
| **MdMTA-BD-R** | **TCGACGGATCCCCGGGAATTCTCAACCGGCCATTTCAACATCC** |
| **MdMTB-AD-F** | **GTACCAGATTACGCTCATATGATGGGACAGAAAGGTTCCATGC** |
| **MdMTB-AD-R** | **ATGCCCACCCGGGTGGAATTCTTACAACAAATTCATATGTCTG** |
| **MdDSK2a-like-AD-F** | **GTACCAGATTACGCTCATATGATGGGTGGCGAGGGCGATTC** |
| **MdDSK2a-like-AD-R** | **ATGCCCACCCGGGTGGAATTCTTACACCCCAGGATTCCCTA** |
| **MdDSK2a-like-AD (1-270 aa)-R** | **ATGCCCACCCGGGTGGAATTCCAAAAATGGCTCCTGAACGTT** |
| **MdDSK2a-like-AD (271-425 aa)-F** | **GTACCAGATTACGCTCATATGAATGCTACAACCGGAAATGCT** |
| **MdDSK2a-like-AD (271-425 aa)-R** | **ATGCCCACCCGGGTGGAATTCTTGCATCATTTCCCTAAGTTG** |
| **MdDSK2a-like-AD (426-547 aa)-F** | **GTACCAGATTACGCTCATATGAATCCAGAGCTACTAAGGCAG** |
| **MdATG8i-1-BD-F** | **TCAGAGGAGGACCTGCATATGATGGGGAAGATCCAATCTTTC** |
| **MdATG8i-1-BD-R** | **TCGACGGATCCCCGGGAATTCTTAGCCAAAGGTTTTCTCGCT** |
| **MdATG8i-2-BD-F** | **AAGCTGATCTCAGAGGAGGACCTGCATATGATGCCTGTCGAGCAGTTTATC** |
| **MdATG8i-2-BD-R** | **TTATGCTAGTTATGCGGCCGCTGCAGTTAAGTTTCATAGATGGAATCC** |
| **MdATG8i-3-BD-F** | **AAGCTGATCTCAGAGGAGGACCTGCATATGATGGGGAAGATCCAGTCCTTC** |
| **MdATG8i-3-BD-R** | **TTATGCTAGTTATGCGGCCGCTGCAGTTAACCAAAGGTTTTCTCGCTG** |
| **MdATG8i-4-BD-F** | **AAGCTGATCTCAGAGGAGGACCTGCATATGATGGGTCGAACAAAAACATTC** |
| **MdATG8i-4-BD-R** | **TTATGCTAGTTATGCGGCCGCTGCAGTCATTGATGATGAAAGCCAAA** |
| **MdATG8c-1-BD-F** | **TCAGAGGAGGACCTGCATATGATGGCCAAAAGCTCCTTCAAG** |
| **MdATG8c-1-BD-R** | **TCGACGGATCCCCGGGAATTCTTAACCGAATGTGTTTTCACC** |
| **MdATG8c-2-BD-F** | **AAGCTGATCTCAGAGGAGGACCTGCATATGATGGCCAAAAGCTCGTTCAAG** |
| **MdATG8c-2-BD-R** | **TTATGCTAGTTATGCGGCCGCTGCAGTTACAACCATTCTTGCTCTTC** |
| **MdATG8c-3-BD-F** | **AAGCTGATCTCAGAGGAGGACCTGCATATGATGGCCAAAAGCTCGTTCAAG** |
| **MdATG8c-3-BD-R** | **TTATGCTAGTTATGCGGCCGCTGCAGTTAAAAGGAACCGAAGACATTC** |
| **MdATG8c-4-BD-F** | **AAGCTGATCTCAGAGGAGGACCTGCATATGATGCCATGTGTTATTGTGGAG** |
| **MdATG8c-4-BD-R** | **TTATGCTAGTTATGCGGCCGCTGCAGTTAACCGAATGTGTTTTCACC** |
| **MdATG8f-1-BD-F** | **TCAGAGGAGGACCTGCATATGATGGCAAAGAGTTACTTCAAG** |
| **MdATG8f-1-BD-R** | **TCGACGGATCCCCGGGAATTCCAGTGGAATCTGATACCCAAATG** |
| **MdATG8f-2-BD-F** | **AAGCTGATCTCAGAGGAGGACCTGCATATGATGATCTGGAAGAGACGGGC** |
| **MdATG8f-2-BD-R** | **TTATGCTAGTTATGCGGCCGCTGCAGTCAGGGGCTACACAGTGGA** |
| **MdATG8g-1-BD-F** | **AAGCTGATCTCAGAGGAGGACCTGCATATGATGAGTTTGAGACGTGCTGAG** |
| **MdATG8g-1-BD-R** | **TTATGCTAGTTATGCGGCCGCTGCAGCTATCCAAATGTGTTCTCGC** |
| **MdATG8g-2-BD-F** | **AAGCTGATCTCAGAGGAGGACCTGCATATGATGACCAAAAGCAGCTTCAAG** |
| **MdATG8g-2-BD-R** | **TTATGCTAGTTATGCGGCCGCTGCAGCTACCCAAATGTGTTCTCGCC** |
| **MdD6PKL2-AD-F** | **GTACCAGATTACGCTCATATGATGGAGCCGTGGCTTGACGACT** |
| **MdD6PKL2-AD-R** | **ACGATTCATCTGCAGCTCGAGCTTTGATCAGATACAACTCGTC** |
| **MdPP2C-AD-F** | **GTACCAGATTACGCTCATATGATGTCGTGCTCCGTCGCACTG** |
| **MdPP2C-AD-R** | **ACGATTCATCTGCAGCTCGAGTCATAAGTAGCGTCTCAATTGGA** |
| **MdSTY13-AD-F** | **GTACCAGATTACGCTCATATGATGAAGGAAAGCTCAGATGGG** |
| **MdSTY13-AD-R** | **ACGATTCATCTGCAGCTCGAGTCACGGTCCTCTTTTCTTTCG** |
| **MdAIRP2-AD-F** | **GTACCAGATTACGCTCATATGATGGGAAAACCGTTCAGGGAC** |
| **MdAIRP2-AD-R** | **ACGATTCATCTGCAGCTCGAGTCACCGCACATAAGAATCATAG** |
| **MdRUB2-AD-F** | **GTACCAGATTACGCTCATATGATGCAGATTTTTGTGAAAACC** |
| **MdRUB2-AD-R** | **ACGATTCATCTGCAGCTCGAGTTATAGGCTACCACCTCGTAGT** |
| **MdDESI1-AD-F** | **GTACCAGATTACGCTCATATGATGGCAGAGGAGAGTCACAAG** |
| **MdDESI1-AD-R** | **ACGATTCATCTGCAGCTCGAGTTAACTCTGTGACATCGTGGAA** |
| **Primer for Split-Luc assay and Subcellular localization assay** | |
| **MdMTAF-attb1** | **GGGGACAAGTTTGTACAAAAAAGCAGGCTGCATGGAGACCCAATCGGAAGGC** |
| **MdMTAR-attb2** | **GGGGACCACTTTGTACAAGAAAGCTGGGTCACCGGCCATTTCAACATCCACTG** |
| **MdATG8i- attb1** | **GGGGACAAGTTTGTACAAAAAAGCAGGCTGCATGGGGAAGATCCAATCTTTC** |
| **MdATG8i- attb2** | **GGGGACCACTTTGTACAAGAAAGCTGGGTCTTAGCCAAAGGTTTTCTCGCT** |
| **MdDSK2a-like-attb1** | **GGGGACAAGTTTGTACAAAAAAGCAGGCTGCATGGGTGGCGAGGGCGATTCG** |
| **MdDSK2a-like-attb2** | **GGGGACCACTTTGTACAAGAAAGCTGGGTC TTACACCCCAGGATTCCCTAAAAG** |
| **MdAIRP2-attb1** | **GGGGACAAGTTTGTACAAAAAAGCAGGCTGCATGGGAAAACCGTTCAGGGAC** |
| **MdAIRP2-attb2** | **GGGGACCACTTTGTACAAGAAAGCTGGGTC TCACCGCACATAAGAATCATAG** |
| **Primer for coimmunoprecipitation assay** | |
| **MdMTA-F** | **CACGCGTTGTACAGAGCTCGGTACCATGGAGACCCAATCGGAAGGCG** |
| **MdMTA-R** | **GGGGACCACTTTGTACAAGAAAGCTGGGTCACCGGCCATTTCAACATCCACTG** |
| **MdDSK2a-like -F** | **GGGGACAAGTTTGTACAAAAAAGCAGGCTGCATGGGTGGCGAGGGCGATTC** |
| **MdDSK2a-like -R** | **GGGGACCACTTTGTACAAGAAAGCTGGGTC TTACACCCCAGGATTCCC** |
| **MdATG8i-F** | **GGGGACAAGTTTGTACAAAAAAGCAGGCTGCATGGGGAAGATCCAATCTTTC** |
| **MdATG8i-R** | **GGGGACCACTTTGTACAAGAAAGCTGGGTCGCCAAAGGTTTTCTCGCTGCT** |
| **MdAIRP2-F** | **GGGGACAAGTTTGTACAAAAAAGCAGGCTGCATGGGAAAACCGTTCAGGGAC** |
| **MdAIRP2-R** | **GGGGACCACTTTGTACAAGAAAGCTGGGTC CCGCACATAAGAATCATAGGG** |
| **Primer for transgenic plants or calli** | |
| **MdDSK2a-like -attb1** | **GGGGACAAGTTTGTACAAAAAAGCAGGCTGCATGGGTGGCGAGGGCGATTC** |
| **MdDSK2a-like -attb2** | **GGGGACCACTTTGTACAAGAAAGCTGGGTCTTACACCCCAGGATTCCC** |
| **MdDSK2a-like RNAi-attb1** | **GGGGACAAGTTTGTACAAAAAAGCAGGCTGCCAACTGACTCAGAACCCGAACA** |
| **MdDSK2a-like RNAi-attb2** | **GGGGACCACTTTGTACAAGAAAGCTGGGTCCTGTCAGTGTTTCGCATCATCT** |
| **MdMTA -F** | **CACGCGTTGTACAGAGCTCGGTACCATGGAGACCCAATCGGAAGGCG** |
| **MdMTA -R** | **TTGTAGTCAAGCTTGCATGCCTGCAGTCAACCGGCCATTTCAACATCC** |
| **MdMTA anti -F** | **CACGCGTTGTACAGAGCTCGGTACCAGTTTTGGCTCAGGAGACGG** |
| **MdMTA anti -R** | **TTGTAGTCAAGCTTGCATGCCTGCAGACCATAGCCGGCATCAGAAC** |
| **Primer for qRT-PCR and mRNA stability assay** | |
| **MdDSK2a-like -F** | **CTCAGGATCCAACTCTGACCA** |
| **MdDSK2a-like -R** | **CCATTTCTCGTAGCTGCCTCA** |
| **MdMTA -F** | **ACCATAGCCGGCATCAGAAC** |
| **MdMTA -R** | **GGTGGGGACGATTTGGGATT** |
| **MdSAG101-F** | **GCGATGGTTTCGTTTACTCC** |
| **MdSAG101-R** | **CCAAATATCGAGGTTTGTGAGGT** |
| **MdEDS1-F** | **TCCCATGCCGTTAGGAGAGA** |
| **MdEDS1-R** | **TTGAATCGACGAGAGCGGA** |
| **MdGOLS4-F** | **GACAAGGTTTCATGGCCTGC** |
| **MdGOLS4-R** | **TGTTCAAGAAATCAACTTGCAGTGT** |
| **MdFLA11-F** | **ACCTTCAGGGTCCTCGGATA** |
| **MdFLA11-R** | **GCTATCATCCCCACCGATGC** |
| **MdIRX7-F** | **ACGCCTCGTGGACTATTGGA** |
| **MdIRX7-R** | **CGCATGTAAGATTCGTGGGTC** |
| **MdIRX15-F** | **GTAAGCACTAAGCGTGGGTGA** |
| **MdIRX15-R** | **TTACACTTGTGCTTGCATTTCC** |
| **Primer for m6A-IP-qPCR assay** | |
| **MdSAG101-F** | **TCGAACCGCTTCACATTGCT** |
| **Md SAG101-R** | **TGTCGAGGCCTTTTCTCTTTG** |
| **MdEDS1-F** | \| **GGAGCATTTGGCCTACGAGA** \|  \| \| --- \| --- \| |
| **MdEDS1-R** | **ACCATCTGAGGCTAAGGGGA** |
| **MdGOLS4-F** | **ACTTTCCCCACACTCCTCATTT** |
| **MdGOLS4-R** | **GGGAAGATGTTGGGGTGATTTG** |
| **MdFLA11-F** | **TCGTTTTGCTAGTCTTCTTCCTCT** |
| **MdFLA11-R** | **TTCTCAAGGACAGCAGTCACG** |
| **MdIRX7-F** | **TGTGGCTCTCTCTCTCCCTC** |
| **MdIRX7-R** | **AGGCGAAGGTGGAGAAATGG** |
| **MdIRX15-F** | **CGTACTCATCCACCAGCACC** |
| **MdIRX15-R** | **CGGCATTTTGAACGTGTCGT** |
| **MD01G1085600-F** | **AATGAGGTGTCGCAGGAACT** |
| **MD01G1085600-R** | **GCTTTCCTTACCAGGCTACCA** |
| **MD05G1092900-F** | **TTACAGCCCCTCACTACCTTCT** |
| **MD05G1092900-R** | **TAGATTTTGGAGTGGCGAGG** |
| **MD15G1096100-F** | **TGTTTGAATGTTGCTTCCTACCAC** |
| **MD15G1096100-R** | **GGTTCATGTGCTCGTCTCCT** |
| **MD03G1084800-F** | **GCCTGCCGTAAGCTCTAGTC** |
| **MD03G1084800-R** | **TGATGTTCTGAATCTGCAGTGTA** |
| **MD05G1351300-F** | **TCGATTCAAGAGTTGGAAGCC** |
| **MD05G1351300-R** | **AGTTGACAGGTTGGTTGGGG** |
| **MD10G1153500-F** | **CATGCGTGGGGTAAAACGAAC** |
| **MD10G1153500-R** | **TGGTGAAAAACGGGGCAGA** |

**Reference**

[1] K. P. Lee, K. Liu, E. Y. Kim, L. Medina-Puche, H. Dong, M. Di, R. M. Singh, M. Li, S. Qi, Z. Meng, J. Cho, H. Zhang, R. Lozano-Duran, C. Kim, *Plant Cell* **2024**, *36*, 746.

[2] L. Zhang, J. Liu, J. Chen, Y. Zhang, C. Qin, X. Lyu, Z. Li, R. Ji, B. Liu, H. Li, T. Zhao, *Adv. Sci.* **2024**, e2410334.

[3] X. Wu, T. Su, S. Zhang, Y. Zhang, C. E. Wong, J. Ma, Y. Shao, C. Hua, L. Shen, H. Yu, *Nat. plants* **2024**, *10*, 469.

[4] T. Sekiguchi, T. Sasaki, M. Funakoshi, T. Ishii, Y. Saitoh, S. Kaneko, H. Kobayashi, *Biochem. Biophys. Res. Commun.* **2011**, *411*, 555.

[5] E. D. Lowe, N. Hasan, J. F. Trempe, L. Fonso, M. E. M. Noble, J. A. Endicott, L. N. Johnson, N. R. Brown, *Acta Crystallogr. D. Biol. Crystallogr.* **2006**, *62*, 177.

[6] Y. Du, G. Hou, H. Zhang, J. Dou, J. He, Y. Guo, L. Li, R. Chen, Y. Wang, R. Deng, J. Huang, B. Jiang, M. Xu, J. Cheng, G. Q. Chen, X. Zhao, J. Yu, *Nucleic Acids Res* **2018**, *46*, 5195.

[7] G. Hou, X. Zhao, L. Li, Q. Yang, X. Liu, C. Huang, R. Lu, R. Chen, Y. Wang, B. Jiang, J. Yu, *Nucleic Acids Res* **2021**, *49*, 2859.

[8] F. Yu, J. Wei, X. Cui, C. Yu, W. Ni, J. Bungert, L. Wu, C. He, Z. Qian, *Nucleic Acids Res* **2021**, *49*, 5779.

[9] Y. Li, X. He, X. Lu, Z. Gong, Q. Li, L. Zhang, R. Yang, C. Wu, J. Huang, J. Ding, Y. He, W. Liu, C. Chen, B. Cao, D. Zhou, Y. Shi, J. Chen, C. Wang, S. Zhang, J. Zhang, J. Ye, H. You, *Nat. Commun.* **2022**, *13*, 6350.

[10] X.-L. Zhang, X.-H. Chen, B. Xu, M. Chen, S. Zhu, N. Meng, J.-Z. Wang, H. Zhu, D. Chen, J.-B. Liu, G.-R. Yan, *Nat. Commun.* **2023**, *14*, 3815.

[11] F. N. Ritonga, S. Chen, *Plants* **2020**, *9*.

[12] C. Yang, H. Yang, Q. Xu, Y. Wang, Z. Sang, H. Yuan, *Phytochemistry* **2020**, *174*, 112346.

[13] J. Browse, Z. Xin, *Curr Opin Plant Biol* **2001**, *4*, 241.

[14] C. Waszczak, M. Carmody, J. Kangasjärvi, *Annu. Rev. Plant Biol.* **2018**, *69*, 209.

[15] S. S. Gill, N. Tuteja, *Plant Physiol Biochem* **2010**, *48*, 909.

[16] N. N. Rudenko, D. V Vetoshkina, T. V Marenkova, M. M. Borisova-Mubarakshina, *Antioxidants* **2023**, *12*, 11.

[17] P. E. Panter, J. R. Panter, H. Knight, Annual Plant Reviews online **2020**, *3*.

[18] L. Bashline, L. Lei, S. Li, Y. Gu, *Mol. Plant* **2014**, *7*, 586.

[19] L. Braidwood, C. Breuer, K. Sugimoto, *New Phytol* **2014**, *201*, 388.

[20] Q. Yin, W. Qin, Z. Zhou, A.-M. Wu, W. Deng, Z. Li, W. Shan, J.-Y. Chen, J.-F. Kuang, W.-J. Lu, *Plant Biotechnol. J.* **2024**, *22*, 413.

[21] A. Bilska-Kos, P. Panek, A. Szulc-Głaz, P. Ochodzki, A. Cisło, J. Zebrowski, *J. Plant Physiol.* **2018**, *228*, 178.

[22] Y. Ding, Y. Shi, S. Yang, *Mol Plant* **2020**, *13*, 544.

[23] Y. Ding, Y. Shi, S. Yang, *New Phytol* **2019**, *222*, 1690.

[24] S. Wilkinson, A. L. Clephan, W. J. Davies, *Plant Physiol.* **2001**, *126*, 1566.

[25] N. Hou, C. Li, J. He, Y. Liu, S. Yu, M. Malnoy, M. Mobeen Tahir, L. Xu, F. Ma, Q. Guan, *New Phytol* **2022**, *234*, 1294.

[26] R. S. Marshall, F. Li, D. C. Gemperline, A. J. Book, R. D. Vierstra, *Mol. Cell* **2015**, *58*, 1053.
